# Identification of *CaPs* locus involving in purple stripe formation on unripe fruit, reveals allelic variation and alternative splicing of R2R3-MYB transcription factor in pepper (*Capsicum annuum L.*)

**DOI:** 10.3389/fpls.2023.1140851

**Published:** 2023-03-28

**Authors:** Ning Li, Yabo Liu, Yanxu Yin, Shenghua Gao, Fangyuan Wu, Chuying Yu, Fei Wang, Byoung−Cheorl Kang, Kai Xu, Chunhai Jiao, Minghua Yao

**Affiliations:** ^1^ Hubei Key Laboratory of Vegetable Germplasm Innovation and Genetic Improvement, Cash Crops Research Institute, Hubei Academy of Agricultural Sciences, Wuhan, China; ^2^ College of Horticulture and Gardening, Yangtze University, Jingzhou, China; ^3^ Department of Agriculture, Forestry, and Bioresources, Plant Genomics Breeding Institute, College of Agriculture and Life Sciences, Research Institute of Agriculture and Life Sciences, Seoul National University, Seoul, Republic of Korea; ^4^ Hubei Hongshan Laboratory, Wuhan, China

**Keywords:** pepper, anthocyanin biosynthesis, fine mapping, purple stripe, transcriptional reputational

## Abstract

The purple color of unripe pepper fruit is attributed to the accumulation of anthocyanins. Only a few genes controlling the biosynthesis and regulation of anthocyanins have been cloned in *Capsicum*. In this study, we performed a bulked segregant analysis of the purple striped trait using an F^2^ population derived from a cross between the immature purple striped fruit line Chen12-4-1-1-1-1 and the normal green fruit line Zhongxian101-M-F^9^. We mapped the *CaPs* locus to an 841.39 kb region between markers M-CA690-Xba and MCA710-03 on chromosome 10. *CA10g11690* encodes an R2R3-MYB transcription factor that is involved in the biosynthesis of anthocyanins as the best candidate gene. Overexpression and silencing in transformed tobacco (*Nicotiana tabacum*) lines indicated that *CA10g11690* is involved in the formation of purple stripes in the exocarp. A comparison of parental sequences identified an insertion fragment of 1,926 bp in the second intron region of Chen12-4, and eight SNPs were detected between the two parents. Additionally, there were 49 single nucleotide polymorphic variations, two sequence deletions, and four sequence insertions in the promoter region. We found that *CA10g11690* undergoes alternative splicing and generates different transcripts. Thus, the functional transcript of *CA10g11690* appeared to be primarily involved in the development of purple phenotype in the exocarp. Our data provide new insight into the mechanism of anthocyanin biosynthesis and a theoretical basis for the future breeding of purple striped pepper varieties.

## Introduction

Fruit color is one of the most critical quality traits for the appearance of pepper (*Capsicum annum* L.) since it is the primary standard used by consumer perceptions to select and purchase these fruits (Jang et al., 2020). The fruit color of pepper is considerably diverse with such colors as green, sulfur-white, yellow, and purple for unripe fruit, and red, yellow, orange, and brown for fully mature fruit. The purple color of unripe pepper fruit is attributed to the accumulation of anthocyanins. Anthocyanins play multiple functional roles in biological processes, such as protecting plants against biotic and abiotic stresses and attracting pollinators (Chalker-Scott, 1999; Ahmed et al., 2015). Additionally, anthocyanins are natural antioxidant compounds that offer beneficial effects on human health, such as protecting against cardiovascular and neurodegenerative disease (Park et al., 2011; Mattioli et al., 2020). Therefore, breeding cultivars of horticultural crops with high anthocyanin contents has become an interesting target (Allan and Espley, 2018).

The biochemistry and enzymology of the anthocyanin biosynthetic pathway (ABP) have been extensively characterized in many plants (Park et al., 2011; Petroni and Tonelli, 2011; Allan and Espley, 2018). This pathway involves two types of genes. One group includes the anthocyanin biosynthetic structural genes, which can encode enzymes of the anthocyanin biosynthesis pathway. The other group is composed of regulatory genes that code for proteins control the transcription of structural genes (Jaakola et al., 2002; Albert et al., 2014; Zhang et al., 2014a). The structural genes are further classified into two groups. One group is early biosynthetic genes (EBGs), including chalcone synthase (CHS), chalcone isomerase (CHI), flavanone 3-hydroxylase (F3H) and flavanone 3,5-hydroxylase (F3’5’H), that produce dihydroflavonols. The other group is the late biosynthetic genes (LBGs), including dihydroflavonol 4-reductase (DFR), anthocyanidin synthase (ANS), flavonol-3-glucosyltransferase (3GT), flavonol-5-glucosyltransferase (5GT), rhamnosyl transferase (RT), and glutathione-S-transferase (GST), that catalyze the synthesis of anthocyanins from leucoanthocyanidins through anthocyanidins (Dubos et al., 2010; Petroni and Tonelli, 2011). The structural genes encode a series of enzymes that directly produced the anthocyanins. The activity of anthocyanin biosynthetic enzymes is mainly regulated at the transcriptional level by the evolutionarily conserved MYB-bHLH-WD repeat (MBW) ternary transcriptional complex, that consist of R2R3-MYB, basic helix-loop-helix (bHLH, or MYC), and tryptophan (W) aspartic (D)-repeat (WDR) proteins (Koes et al., 2005; Albert et al., 2014; Xu et al., 2015). Among the regulators, R2R3-MYB transcription factors are considered to be key components to direct activate the anthocyanin biosynthesis genes (ABGs) by forming MBW complex to promote anthocyanin biosynthesis. In contrast, the MBW complex normally activates the LBGs (Saitoh et al., 2004; Guo et al., 2014; Sun et al., 2018).

Substantial studies about anthocyanin biosynthesis have been explored in solanaceous vegetable crops, which include the identification of components, analysis of the patterns of expression of ABGs, and gene mapping (Schreiber et al., 2012; Kiferle et al., 2015; Meng et al., 2015; Jiang et al., 2016; Sun et al., 2020). For example, four highly homologous and adjacent R2R3-MYB transcription factors, including *SlAN2, SlAN2-like, SlANT1* and *SlANT1-like*, have been identified to activate abundant anthocyanins in tomato (*Solanum lycopersicon*) (Schreiber et al., 2012; Kiferle et al., 2015; Meng et al., 2015). *SmMYB1*, *SmMYB35* and *SmMYB113* positively regulate anthocyanin biosynthesis in eggplant (*S. melongena*) (Zhang et al., 2014b; Li et al., 2021; Yang et al., 2022). Even though several studies of anthocyanins have been carried out on the Solanaceae, current studies have not yet been clearly explained for the biosynthesis and regulation of anthocyanins in *Capsicum*. The best-known regulator is the *A* gene that encodes an R2R3-MYB transcription factor that controls the accumulation of anthocyanins in various parts of the pepper plants, including the foliage, flowers, and immature fruit. The *A* gene is homologous to the *PhAn2* gene of Petunia (*Petunia hybrida*) and *SlAN2* of tomato. Therefore, these genes were also designated *MybA*, *CaMYB113*, *CaMYB* and *CaAN2* (Borovsky et al., 2004; Jung et al., 2019; Filyushin et al., 2020; Wang et al., 2022). The reason for the accumulate of anthocyanin in purple-fruited peppers is a non-LTR retrotransposon (Ca-nLTR-A) insertion in the promoter region of *CaAN2* (Jung et al., 2019; Ohno et al., 2020). Additionally, a *Ca3GT* gene that encodes UDP-glycosyltransferase was fine-mapped on chromosome 10 (Liu et al., 2020). It controls anthocyanin biosynthesis in mature unripe fruit in pepper. More recently, a novel R2R3-MYB transcription factor, *CaAN3*, was also identified as a fruit-specific activator of anthocyanin biosynthesis in pepper (Byun et al., 2022). However, there are different purple phenotypes in pepper cultivars, which suggests that there may be various mechanisms for the accumulation of anthocyanin. Therefore, the information on genetic resource mining, new locus identification, and the prediction of regulatory mechanisms are essential to deepen our understanding of anthocyanin biosynthesis in *Capsicum* species.

In this study, the purple striped spontaneous mutant ‘Chen12-4-1-1-1-1’ provides a tremendous opportunity to study the molecular mechanisms for underlying anthocyanin biosynthesis in pepper. We first carried out fine mapping of the *CaPs* locus involved in the formation of purple stripes on the unripe fruit of pepper using bulked-segregant analysis-seq (BSA-seq). The *CA10g11690* that encoded the R2R3-MYB transcription factor was considered as the candidate gene of *CaPs.* We verified the *CaPs* candidate gene using virus-induced gene silencing (VIGS) and genetic transformation approaches. The molecular marker that co-segregated with the *CaPs* locus was developed for pepper breeding. We also discovered that *CA10g11690* undergoes alternative splicing and generates different transcripts and thus, appeared to be primarily involved in the purple phenotype in the exocarp. Our results provide new insight into the anthocyanin biosynthetic molecular mechanism and more information for the future breeding of purple striped pepper varieties.

## Materials and methods

### Plant materials, mapping populations and phenotyping

**Figure 1:**
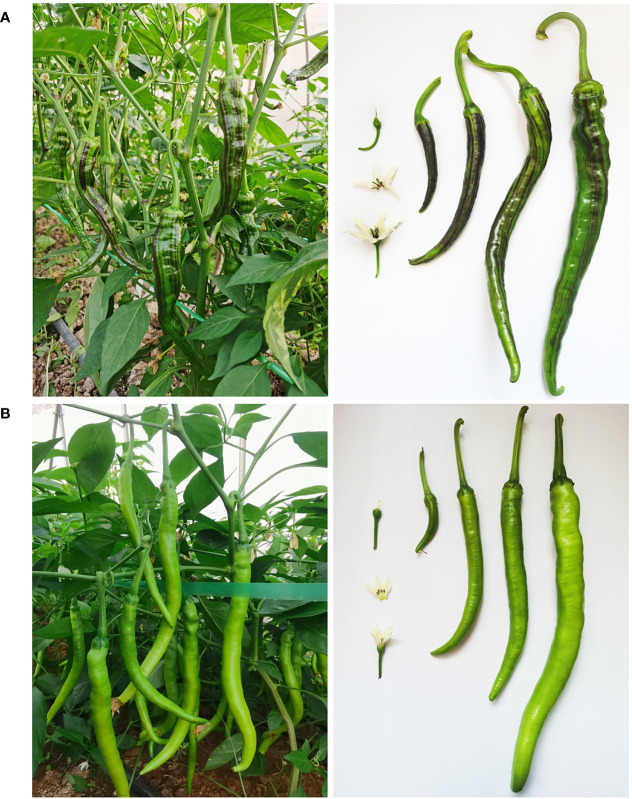
Phenotype of two parents. **(A)** The plants, fruits and flowers of Chen12-4. **(B)** The plants, fruits and flowers of Z101-M.

The purple striped spontaneous mutant Chen12-4-1-1-1-1 (Chen12-4 for short, **Figure 1A**) was derived from a hybrid combination designated XianjiaoF_1_-No.12 from a Chinese pepper breeder Gongrong Chen. The hybrid combination was first planted in the spring in 2017, and all the F_1_ individuals consistently produced unripe green fruit. In the F_2_ generation, several plants with purple striped unripe fruit were observed in the field. In addition, purple filaments and styles were observed in the purple striped individuals. This phenotype could be stably inherited in the F_3_ generation, and the mutant individuals were selected and subsequently self-crossed. After continuous self-crossing for five generations, the purple striped mutant inbred line Chen12-4-1-1-1-1 was obtained as the female parent. The *C. annuum* inbred line Zhongxian101-M-F_9_ (Z101-M, **Figure 1B**) with normal green fruit was used as another parental line to create the segregating population (CZ-F_2_ population). The F_2_ population of 252 individuals was grown in the autumn for genetic analysis and initial mapping. A larger F_2_ population of 741 individuals was planted in autumn for fine mapping. Another F_2_ population (CC-F_2_ population) was also developed, which was generated from the purple striped spontaneous mutant Chen12-4 and the green fruit inbred line CA1403-M-F_8_ to further verify the co-separation of genotype and phenotype. In addition, several pepper inbred lines, including Mexico38-M-F_9_ (M38, *CaAn2* genotype), Zifangjiao (ZFJ, *CaAn3* genotype), Chen12-3-5-1-1-1 (Chen12-3-5, a sister line of Chen12-4 with normal green fruit) and other lines, were used in this study. All plants were grown in a plastic greenhouse at the Vegetable Experimental Station of Cash Crops Research Institute at the Hubei Academy of Agricultural Sciences (Wuhan, China).

### Genetic analysis of the gene (s) that control the purple stripy trait

Genetic characterization of the gene (s) controlling the purple stripy of unripe fruit in Chen12-4 was analyzed based on the phenotype of F_1_ hybrid and F_2_ population derived from Chen12-4 × Z101-M. The exocarp color of unripe fruit in the pepper plants was monitored by visual observation. A chi-square test (*χ^2^*) was carried out to test the phenotypic data for its goodness-of-fit to Mendel’s inheritance segregation ratios.

### Parental resequencing and bulked segregant analysis analysis

An equal amount of genomic DNA (5 µg) from 30 purple striped and 30 green fruit plants in 252 individuals of CZ-F_2_ population were pooled to construct a purple striped pool (P-pool) and a green pool (G-pool) for BSA-seq. DNA libraries of two parents were also constructed for genomic resequencing. The sequencing depth of two parents and two descendant pools was about 20× sequencing depth using the MGISEQ-2000 platform (MGI Tech Co., Ltd., Shenzhen, China) at Frasergen Bioinformatics Co., Ltd. (Wuhan, China). The raw data were filtered using SOAPnuke software (v2.1.0) to remove adapter contamination and low-quality reads (Chen et al., 2018). The filtered high-quality clean reads were mapped to pepper CM334 reference genome (https://solgenomics.net/ftp/genomes/Capsicum_annuum/C.annuum_cvCM334/, version 1.55) using a Burrows-Wheeler alignment (BWA) tool (Li and Durbin, 2009; Langmead and Salzberg, 2012; Kim et al., 2014). The aligned files were converted to SAM/BAM files using SAM tools and then applied to SNP calling filter (Li et al., 2009; Abe et al., 2012). SNP index and the Δ (SNP index) values were used to identify candidate genomic regions associated with purple stripy trait. The average SNP indices of the SNPs located in each genomic region were calculated using a sliding window analysis with a 2-Mb window range and a 50-kb step size. The Δ (SNP index) was derived from the calculated SNP index for each pool, and this process was repeated 10,000 times for each read depth.

### Development of molecular markers and the prediction of candidate gene

Cleaved amplified polymorphic sequence (CAPS) markers were developed based on the polymorphism between genomic sequences of two parents in the candidate region (**Supplementary Table S1**). PCR amplification was carried out, and then the products were digested by restriction endonuclease enzymes. The digests were separated in 1%-2% agarose gel electrophoresis. After narrowing down the genomic region, the candidate genes were predicted using the *C. annuum* CM334 reference genome database on Genome GBrowse of the PepperHub (http://pepperhub.hzau.edu.cn/gbrowse2/index.php) (Liu et al., 2017) and their annotations were obtained with SGN BLAST (https://solgenomics.net/tools/blast/). Additionally, a SCAR marker set developed based on the sequence polymorphism of the candidate *CaPs* locus was used for molecular marker assisted selection.

### Analysis of gene expression

The expression of candidate genes and other key genes involved in anthocyanin biosynthesis was examined by real-time quantitative reverse transcription PCR (qRT-PCR). Primers used are showed in **Supplementary Table S1**. Total RNA was extracted using the TRIzol reagent (Invitrogen, Carlsbad, CA, USA) from different tissues of pepper or the leaves of transgenic tobacco (*Nicotiana tabacum*). The purified RNA (0.5 μg) was reverse transcribed into first-strand cDNA using a HiScript II 1st-Strand cDNA Synthesis kit (Vazyme Biotech, Nanjing, China). qRT-PCR was performed with the TransStart^®^ Green qPCR SuperMix Kit (TransGen Biotech) on a QuantStudio 5 Real-Time PCR System (Thermo Fisher Scientific). The *CaUBI3* (AY486137.1) and *NtActin* (AB158612.1) were used as the reference genes (Zhong et al., 2020), respectively. The relative expression of the target genes was calculated using the 2^−ΔΔCt^ method (Livak and Schmittgen, 2001; Wan et al., 2011). Each reaction was performed in triplicate.

### Cloning and sequencing analysis of candidate genes

Gene-specific primers of *CA10g11690* were designed to amplify the full lengths of genomic DNA (gDNA) and entire coding sequence (CDS) from two parents and several pepper inbred lines. An approximately 1,700 bp sequence of *CA10g11690* upstream ATG was also cloned to analyze the sequence variance of the promoter region. The primers were designed according to the *C. annuum* CM334 (UCD 10X) reference genome (https://solgenomics.net/ftp/genomes/Capsicum_annuum/C.annuum_UCD10X/, version 1.0) and *C. annuum* CM334 (http://peppergenome.snu.ac.kr/, version 2.0) (**Supplementary Table S1**). The PCR amplifications of gDNA and promoter region of *CA10g11690* were carried out according to the user manual with 2 × Phanta Max Master Mix (Vazyme). The PCR amplification of CDS of *CA10g11690* was conducted in a total volume of 25 μL that contained 2 μL (10 ng) of cDNA, 0.5 μL for each forward and reverse primer (10 pmol/μL), 13 μL 2 × SuperTaq PCR StarMix (Dye) (GenStar, Kangrun Biotech, Beijing, China). The amplicons were separated on a 1.5% agarose gel, cloned, and purified with an EasyPure^®^ Quick Gel Extraction Kit (TransGen) according to the manufacturer’s instructions. The purified products were then introduced into a TA/Blunt-Zero Cloning Kit (Vazyme) and transformed into *E. coli* DH5α chemically competent cells (AngYu Biotechnologies, Shanghai, China) according to the manufacturer’s instructions. Nucleotide and protein sequence alignment analyses were conducted by CLUSTALW (https://www.genome.jp/tools-bin/clustalw) and drawn using GeneDoc Software Version 2.70.

### Transformation of *Nicotiana tabacum* plants

The overexpression vectors of *CA10g11690* gene were generated by cloning into the *Kpn*I + *Xho*I digestion sites of the pMV2 vector driven by the cauliflower mosaic virus 35S promoter (Yang et al., 2011). Four different overexpression vectors were constructed. *CaMV35S::c690^Long-Z101^* included the long transcript with 1,087 bp from Z101-M. *CaMV35S::c690^Long-ZFJ^* included the long transcript with 1,015 bp from ZFJ. *CaMV35S-promoter::c690^Short-Chen12-4^* included the short transcript with 702 bp from Chen12-4, and *CaMV35S-promoter::c690^Short-ZFJ^* included the short transcript with 702 bp from ZFJ. The constructed vectors were transferred into *Agrobacterium* GV3101 using the freeze-thaw method and then transformed into *N. tabacum*. We transformed the construct into *N. tabacum* using the method described by Horsch et al. (1989). The primers used for overexpression vector construction in this study are showed in **Supplementary Table S1**.

### Virus−induced gene silencing of *CA10g11690*

To study the function of *CA10g11690*, the specific CDS of the *CA10g11690* gene were used to construct the VIGS vector. The fragment that was approximately 200 bp in the specific coding regions of the *CA10g11690* gene was inserted into the pTRV2-C2b vector at the *Sam*I site to construct the pTRV2-C2b-CA10g11690 vector. A mixture of cultures containing 1:1 volume ratio of pTRV1-C2b and pTRV2-C2b was used as tobacco rattle virus (TRV) control. *A. tumefaciens* cells that harbored pTRV1-C2b and pTRV2-C2b-CaPDS were used as reporters, and pTRV1-C2b and pTRV2-C2b-CA10g11690 were used to silence *CA10g11690.* The mixed induction medium was adjusted to OD_600_ = 0.004, and then infiltrated on the cotyledons of two-week-old Chen12-4 peppers. The effect of VIGS was determined according to Zhou et al. (2021).

### RNA-seq analysis

RNA samples taken from the exocarp of Chen12-4 and Chen12-4-VIGS690 (*TRV2-CA10g11690* silencing lines) at 15 days post-anthesis (DPA) were analyzed using RNA-seq. The RNA samples were sent to Frasergen Bioinformatics Co., Ltd. to generate libraries and then sequence using an MGISEQ-2000 platform (MGI Tech Co. Ltd.). After filtration by BBTools (https://jgi.doe.gov/data-and-tools/bbtools) to remove the low-quality reads, the clean reads were then aligned to the *C. annuum* CM334 genome (version 1.55) using the HISAT2 program (https://github.com/DaehwanKimLab/hisat2). The level of expression of mRNA was calculated by fragments per kilobase of transcript per million mapped reads (FPKM). DESeq software was used to standardize the number of mRNA counts in each sample. Finally, differentially expressed genes was detected according to the screening criteria of |log2 (foldchange)| ≥1 and FDR (False Discovery Rate) < 0.01.

### Quantification of total anthocyanins

Anthocyanins in the exocarp of pepper fruit and *N. tabacum* leaves were extracted and quantified through UV-visible spectroscopy with slight modifications (He et al., 2016). The freeze-ground samples (0.1 g) were extracted with 10 mL of 99% methanol and 1% HCl followed by vortexing and then incubated for 6 h at 25°C in the dark. The mixtures were subsequently centrifuged and measured by spectrophotometer (MAPADA, Shanghai, China) at 530 nm and 700 nm in 0.1 M potassium chloride buffer (pH 1.0) and 0.45 M sodium acetate buffer (pH 4.5), respectively. The diluted emulsions were equilibrated for 1.5 h at 37 °C in the dark. Total anthocyanin content was calculated as previously described (Giusti and Wrolstad, 2001). The value used for each sample was the mean of three independent biological replicates.

## Results

### Genetic analysis of purple stripy fruit phenotype of Chen12-4 with Z101-M

Twenty F_1_ individuals from the cross between Chen12-4 with Z101-M exhibited a purple striped fruit phenotype (**Figures 1A, B**; **Supplementary Figure 1**), indicating that the purple striped fruit trait was dominant. The 252 F_2_ individuals segregated into 193 purple striped unripe fruit individuals and 59 green unripe fruit individuals, which agreed well with the expected 3:1 Mendelian ratio (*χ* ^2^ = 0.065, P > 0.05) (**Table 1**). The results suggest that the purple striped trait in Chen12-4 × Z101-M populations was controlled by a single dominant locus, which was designated *CaPs* (Purple striped).

**Table 1:** Genetic analysis of the purple stripy trait in the segregation populations.

Generation	Total	Number of Plants		The expected segregation ratio	Chi-square value
		Purple stripy fruit	Green fruit		
Z101-M	20	0	20		
Chen12-4	20	20	0		
F_1_	20	20	0		
F_2_	252	193	59	3:1	0.065

### The *CaPs* candidate gene is mapped on an 841.39 kb region in pepper chromosome 10

**Figure 2:**
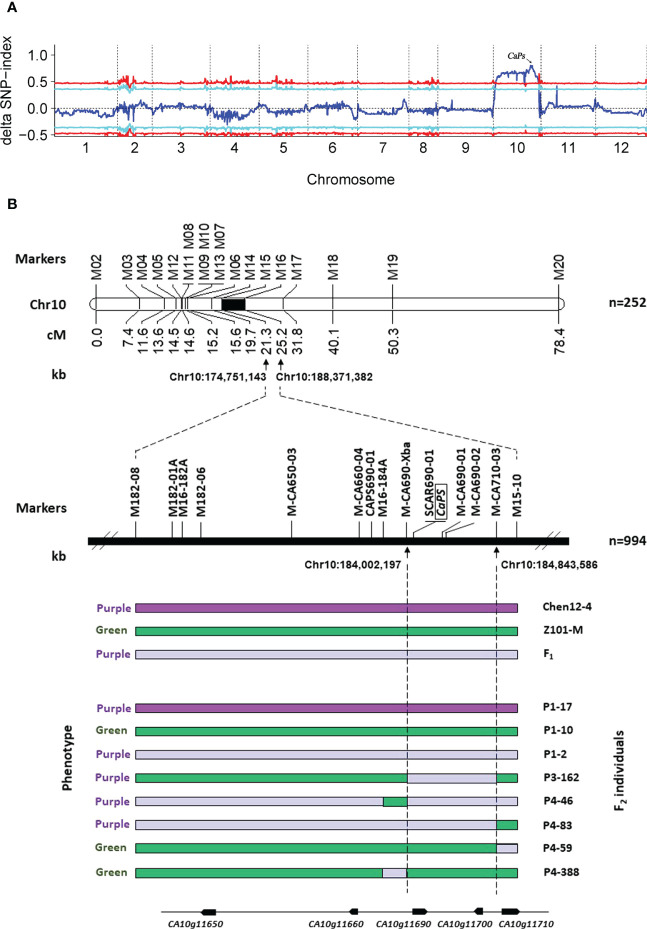
Genetic map, and identification of candidate *CaPs* locus in pepper. **(A)** Graphs of ΔSNP-index for SNP-index-based association analysis. The 12 chromosomes are represented along the x-axis, and the ΔSNP-index values are shown along the y-axis. **(B)** Genetic and physical maps of the *CaPs* gene and candidate gene analysis. *CaPs* was mapped to chromosome 10, between 184,002 kb and 186,844 kb in CM334 genome version 1.55. The genotypes of F_2_ plants homozygous for the green or purple *CaPs* allele or heterozygous are shown as green, dark purple, and light purple bars, respectively. Eight bars at the bottom represent the genotypes of informative individuals with recombination events.

BSA-seq was employed to preliminarily map the *CaPs* gene. Two pools, P-pool and G-pool, generated 67.7 Gb and 70.1 Gb of clean data after filtering, respectively. The Δ(SNP-index) graph was calculated based on the data of P-pool and G-pool against the reference genome CM334. As a result, an obvious peak in the ΔSNP values on chromosome 10 was observed, which indicated that one strong major locus governed the purple striped trait in this region (**Figure 2A**). To verify the candidate regions, linkage mapping was used to analyze the results based on the F_2_ individuals of Chen12-4 × Z101-M using 20 polymorphic CAPS markers that were distributed equidistantly on pepper chromosome 10. They were initially developed based on the whole-genome resequencing of the two parents. These markers were used to identify the genotypes of a subset of 252 CZ-F_2_ individuals (CZ-Population 1) to construct a primary genetic map. The results revealed that seven, seven, two, and 10 recombinants were identified in the four markers (M14, M15, M16, and M17), respectively. Therefore, the causal gene was initially mapped between the two markers, M15 and M16, within a region of 13.62 Mb. We then used an additional 10 markers and a larger mapping population of 994 CZ-F_2_ individuals to further narrow down the genomic region of *CaPs.* As a result, seven and 11 recombinants were identified between marker M-CA690-Xba and M-CA710-03, respectively, and the physical distance of two markers was approximately 841.39 kb (**Figure 2B**).

### *CA10g11690* is the candidate gene for *CaPs*

**Figure 3:**
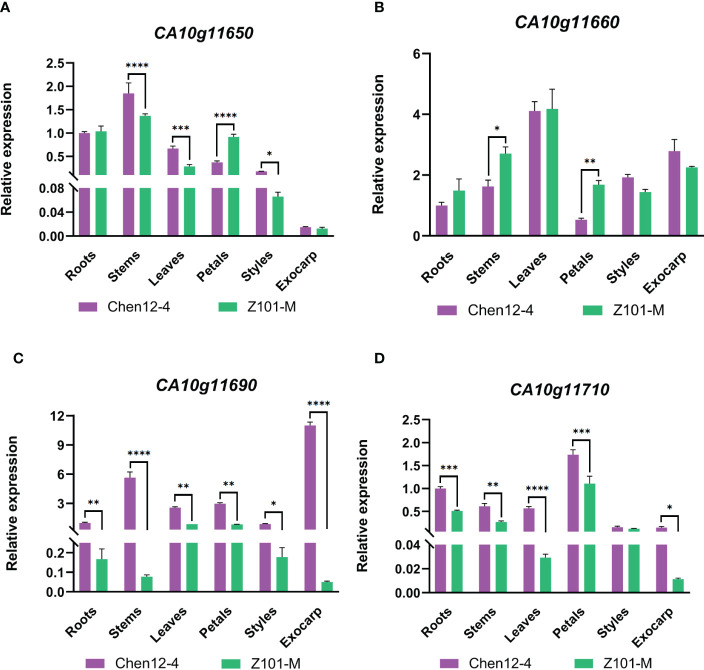
qRT-PCR analysis of the annotated candidate genes in stems, leaves, flowers, mature unripe fruit, and ripe fruit of the two parental lines. **(A)** The patterns of expression of *CA10g11650* in different tissues. **(B)** The patterns of expression of *CA10g11660* in different tissues. **(C)** The patterns of expression of *CA10g11690* in different tissues. **(D)** The patterns of expression of *CA10g11710* in different tissues. The significant differences were identified using T-test, asterisks indicate a significant difference. ****P<0.0001, ***P<0.001, **P<0.01, *P<0.05.

Genome GBrowse of the PepperHub was used to identify two genes, *CA10g11690* and *CA10g11700*, that were in this interval. The gene annotation revealed that *CA10g11690* encodes an R2R3-MYB transcription factor, which might be involved in anthocyanin regulation, while *CA10g11700* encodes a phosphatase. Thus, *CA10g11690* was a likely candidate for the target locus. Notably, four adjacent MYB transcription factors, *CA10g11650*, *CA10g11660*, *CA10g11690* and *CA10g11710*, were in the genomic region on chromosome 10 in pepper. Previous studies showed that the region on the distal part of the long arm of chromosome 10 that is related to the accumulation of anthocyanins was strongly conserved in the domesticated Solanaceae (Borovsky et al., 2004). Although *CA10g11690* was the best candidate gene in this interval, we still examined the expression of these four *MYB* genes in more detail using qRT-PCR. The expression of *CA10g11690* was significantly upregulated in the exocarp of Chen12-4 fruit compared with that of Z101-M (**Figure 3**). Additionally, the qRT-PCR analysis revealed that only *CA10g11690* was significantly differentially expressed among the four genes with a level of expression that increased approximately 9.95-fold in the purple zone compared with the green zone in the exocarp of Chen12-4 fruit (**Supplementary Figure 2**). Taken together, the results suggest that *CA10g11690* is the best candidate gene for the *CaPs* locus.

### Sequence analysis of the candidate gene and development of functional marker

**Figure 4:**
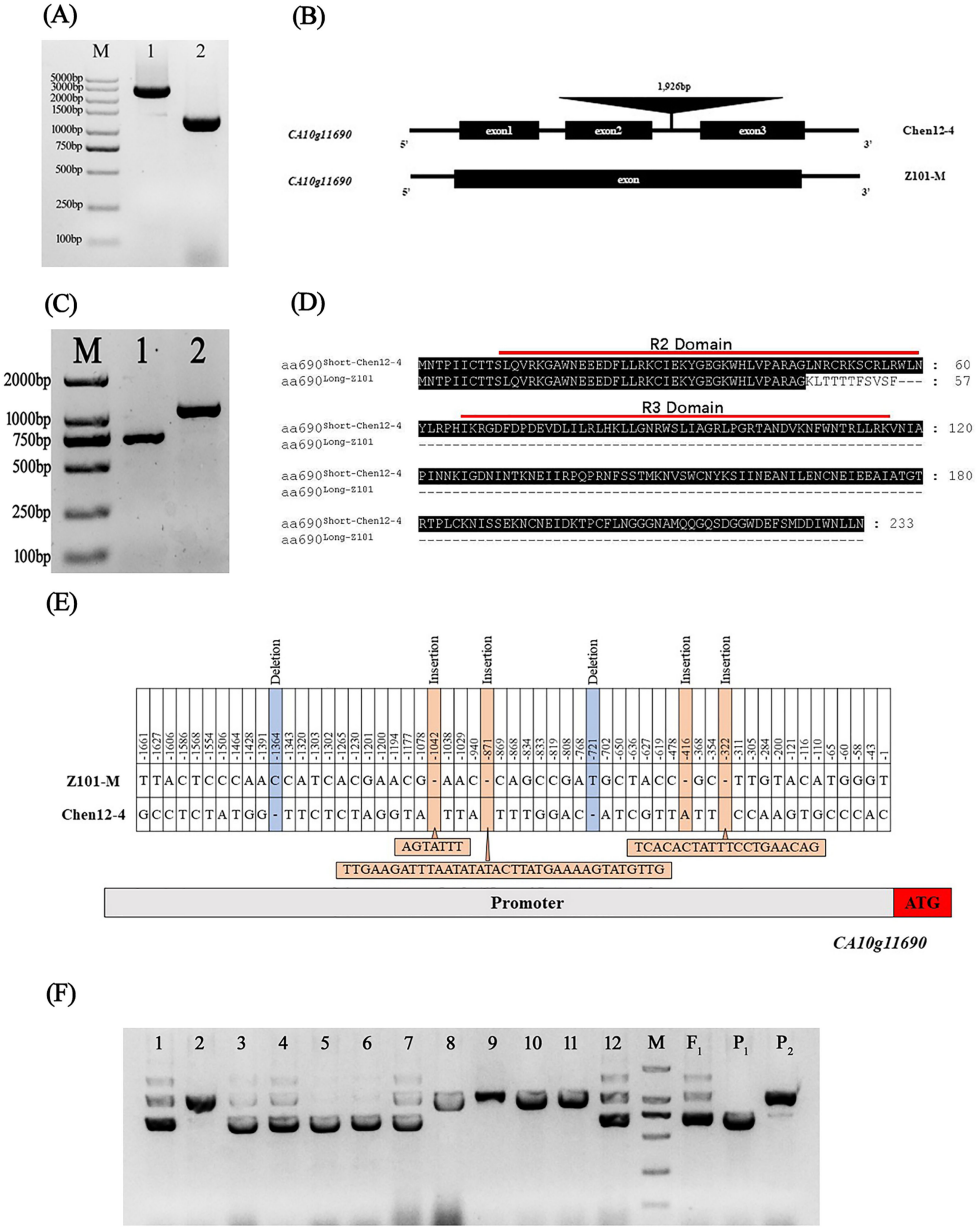
Sequence analysis of *CA10g11690* in two parents and development of a functional Marker. **(A)** PCR products for the gDNA of the *CA10g11690* in two parents, M: Marker DL5000, 1: ‘Chen12-4’, 2: ‘Z101-M’. **(B)** The gene structure of *CA10g11690* in two parents. **(C)** RT-PCR products for the CDS of the *CA10g11690* in two parents, M: Marker DL2000, 1: ‘Chen12-4’, 2: ‘Z101-M’. **(D)** Putative amino acid sequences of two different transcripts. **(E)** Sequence variation analysis in the promoter region of *CA10g11690* in two parents. **(F)** PCR fragments amplified using SCAR marker SCAR690-01 in CZ-F_2_ population. 1-12: F_2_ individuals, M: Marker DL2000, F_1_: Chen12-4 × Z101-M, P_1_: Chen12-4, P_2_: Z101-M.

Specific primers were designed to amplify the genomic sequences, CDSs, and promoter region (approximately 1.7 kb) of *CA10g11690* to analyze the difference between the two parents. A sequence analysis showed that the genomic sequences of *CA10g11690* were distinctly different in Chen12-4 and Z101-M. The genomic sequence of *CA10g11690* was 1,341 bp in Z101-M, whereas it increased to 3,267 bp in Chen12-4. An insertion fragment of 1,926 bp was identified in the second intron region of Chen12-4, and eight SNPs were detected in the genomic DNA of the *CA10g11690* sequence between the two parents (**Figures 4A, B**; **Supplementary File 1**). The amplification of CDS generated one transcript that was designated *c690^Long-Z101^* in Z101-M, while there was another spliced transcript *c690^Short-chen12-4^* in Chen12-4 (**Figure 4C**). Further sequencing showed that *c690^Long-101^* was 1,087 bp long. It contained an open reading frame (ORF) that encoded a deduced protein of only 57 amino acids owing to the premature stop codon TAA. *c690^Short-Chen12-4^* was 702 bp long, but it had an intact ORF that encoded a protein of 233 amino acids (**Supplementary File 2**). Thus, the *c690^Short-Chen12-4^* transcript could encode protein that contained the intact characteristic domain of R2R3-MYB transcription factors, while the *c690^Long-Z101^* transcript only encoded an incomplete R2 domain and harbored the absence of the R3 domain that contained the bHLH-binding signature (**Figure 4D**). It was known that the conservative [D/E]Lx2[R/K]x3Lx6Lx3R motif in the R3 domain was the central feature for proteins to interact with bHLH factors. The absence of the R3 domain in *c690^Long-Z101^* transcript might be fail to form MBW complex to activate the expression of anthocyanin biosynthetic genes. These results suggest that the generation of an alternative splicing variant of *CA10g11690* could affect the biosynthesis of anthocyanins in the green fruit parental line. We further analyzed the promoter region sequences of the two parents in detail. The BLASTed sequence in the promoter region of *CA10g11690* had 51 point-mutations in the purple stripes parent Chen12-4. In addition, 20 bp, 1 bp, 35 bp, and 7 bp sequence insertions and 1 bp sequence deletions were found at -322 bp, -416 bp, -871 bp, -1,042 bp, -721 bp, and -1,364 bp, respectively (**Figure 4E**).

Based on the 1,926 bp fragment insertion in Chen12-4, a codominant SCAR molecular marker, SCAR690-01 (**Table 2**), was designed and used for PCR-based testing of the purple striped phenotype of the F_2_ individuals. As shown in **Figure 4F**, SCAR690-01 amplified the large product (1,112 bp) in the nonpurple parent Z101-M, the small product (732 bp) in the purple striped parent Chen12-4, and three products (1,844 bp, 1,112 bp and 732 bp) in the heterozygous individuals. The polymorphic bands that were amplified with SCAR690-01 completely co-segregated with the purple striped phenotype in both the CZ-F_2_ and CC-F_2_ populations, which further indicated that *CA10g11690* was the gene that controlled the purple striped in Chen12-4. Moreover, the codominant marker SCAR690-01 was useful in the molecular marker-assisted selection of purple striped pepper.

**Table 2:** Primer sequences for the SCAR marker specific for *CaPs* gene.

Primer	Sequence (5’–3’)
SCAR690-01_F	GTGGCACCTTGTTCCTGCTA
SCAR690-01_R1	TAGAGCTTTTGACCCCGGCT
SCAR690-01_R2	TGCAGTTAGCCCAACTACTACAG

### Gene expression analysis of *CA10g11690* and genes involved in anthocyanin biosynthesis

**Figure 5:**
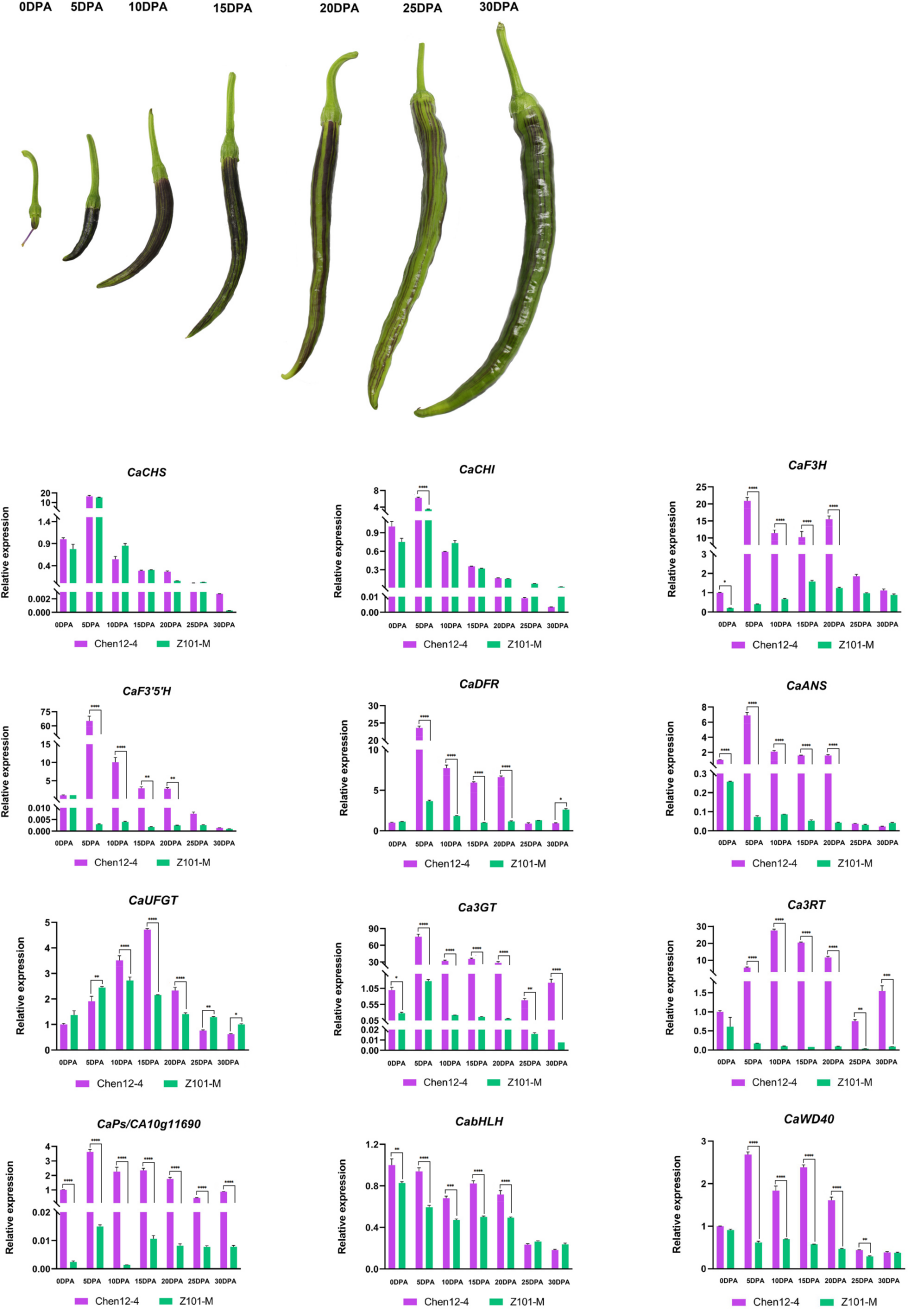
Expression analysis of *CA10g11690* and other anthocyanin biosynthesis genes in two parents at differernt development stages. 0DPA, ovary at anthesis; 5 DPA, young fruit at 5 days post-anthesis; 10 DPA, young fruit at 10 days post-anthesis; 15 DPA, young fruit at 15 days post-anthesis; 20 DPA, young fruit at 20 days post-anthesis mature; 25 DPA, young fruit at 25 days post-anthesis; 30 DPA, young fruit at 30 days post-anthesis. The significant differences were identified using T-test, asterisks indicate a significant difference. ****P<0.0001, ***P<0.001, **P<0.01, *P<0.05.

The patterns of expression of *CA10g11690* in the roots, stems, leaves, petals, styles and exocarps of mature unripe fruit of the two parental lines were examined by qRT-PCR. *CA10g11690* was highly expressed (5.04- to 217.48-fold) in the styles, stems, and exocarps of Chen12-4 than Z101-M (**Figure 3C**). During the ripening stages of pepper fruit in Chen12-4, *CaCHS*, *CaCHI* and *CaF3’5’H* were only highly expressed in young fruit at 5 DPA (**Figure 5**). The genes *CaF3H, CaDFR, CaANS, Ca3GT* and *CA10g11690* were highly expressed in the young fruit at 5, 10, 15 and 20 DPA with the highest expression at 5 DPA. *Ca3RT* was also highly expressed in the young fruit at 5, 10, 15 and 20 DPA with the highest expression at 10 DPA. Gene *CaWD40* was highly expressed in the young fruit at 5 DPA. *CaUFGT* had its highest level of expression at 15 DPA. *CabHLH* did not differ in its levels of transcripts. Compared with the green pepper parental line Z101-M, the EBGs were highly expressed during the whole process, but *CaCHS* and *CaCHI* were relatively highly expressed at 5 DPA in Z101-M. There was no significant difference in the levels of expression of *CaUFGT*, *CabHLH* and *CaWD40* between the green and purple parental lines.

### Silencing of *CA10g11690* leads to the disappearance of purple stripes and the downregulation of anthocyanin biosynthetic genes in Chen12-4

**Figure 6:**
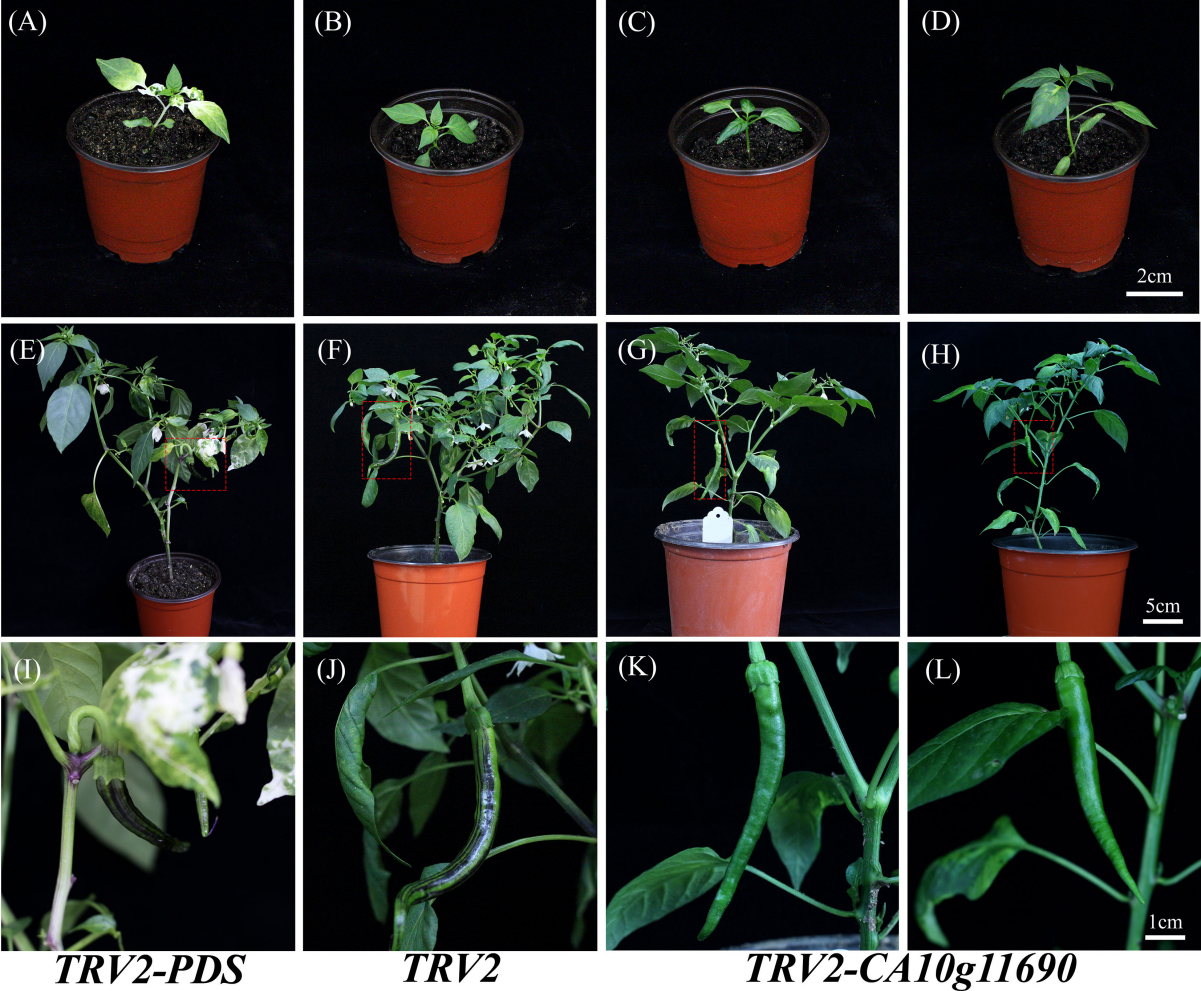
Silencing the candidate gene *CA10g11690* in Chen12-4 *via* mediated VIGS and gene expression patterns. **(A, E, I)** are PDS-silenced plants. **(B, F, J)** are TRV2-treated plants. **(C, D, G, H, K, L)** are *CA10g11690*-silenced plants; The red boxed regions in **(E–H)** are magnified in **(I–L)** for better visualization. Representative phenotypes of fruits from *TRV2-PDS*, *TRV2*, and *TRV2*-*CA10g11690* plants. *TRV2-PDS* and *TRV2* plants were used as negative and positive controls, respectively, and only produced purple fruits. *TRV2-CA10g11690* plants exhibited a complete loss of purple stripes on the surface.

**Figure 7:**
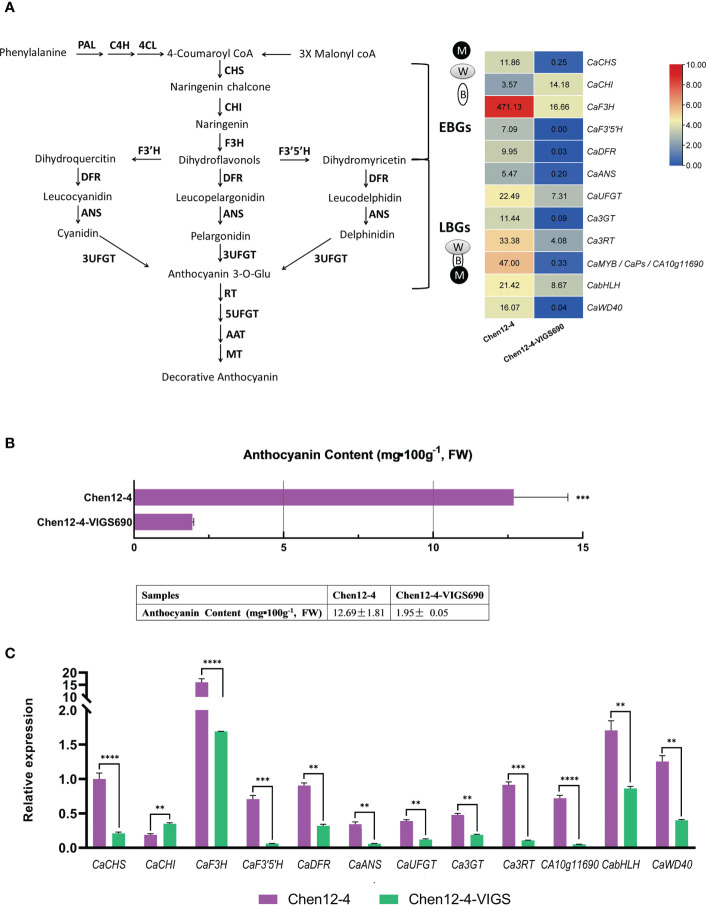
Gene expression patterns of the candidate gene *CA10g11690* in Chen12-4 and Chen12-4-VIGS lines. **(A)** Heatmap of the expression of genes related to the anthocyanin biosynthetic pathway *via* transcriptome analysis. **(B)** Anthocyanin content of Chen12-4 and its *CA1011690-*silenced plants. **(C)** The levels of expression of anthocyanin biosynthetic genes using qPCR analysis. The values of fragments per kilobase of exon per million fragments mapped (FPKM) were used to construct the heat map. The significant differences were identified using T-test, asterisks indicate a significant difference. ****P<0.0001, ***P<0.001, **P<0.01.

To further investigate the functional role of *CA10g11690*, we silenced this gene in Chen12-4 using a modified VIGS method. The purple stripes completely disappeared on the surface of Chen12-4 in unripe fruit that were subjected to *TRV2-CA10g11690* silencing (**Figures 6A–L**). Additionally, the expression of *CA10g11690* was significantly reduced in the respective silenced plants, illustrating the accuracy of VIGS results based on qRT-PCR (**Figure 7C**). The content of anthocyanin in the exocarps was determined in fruit treated with *TRV2-CA10g11690*. The contents of anthocyanin in Chen12-4-VIGS690 lines were 1.95 mg·100 g^-1^ fresh weight, while those of the control plants Chen12-4 were 12.69 mg·100 g^-1^ fresh weight at 15 DPA (**Figure 7B**). These results demonstrated that *CA10g11690* plays a distinct role in the formation of purple stripes of the exocarp in unripe fruit.

**Figure 8:**
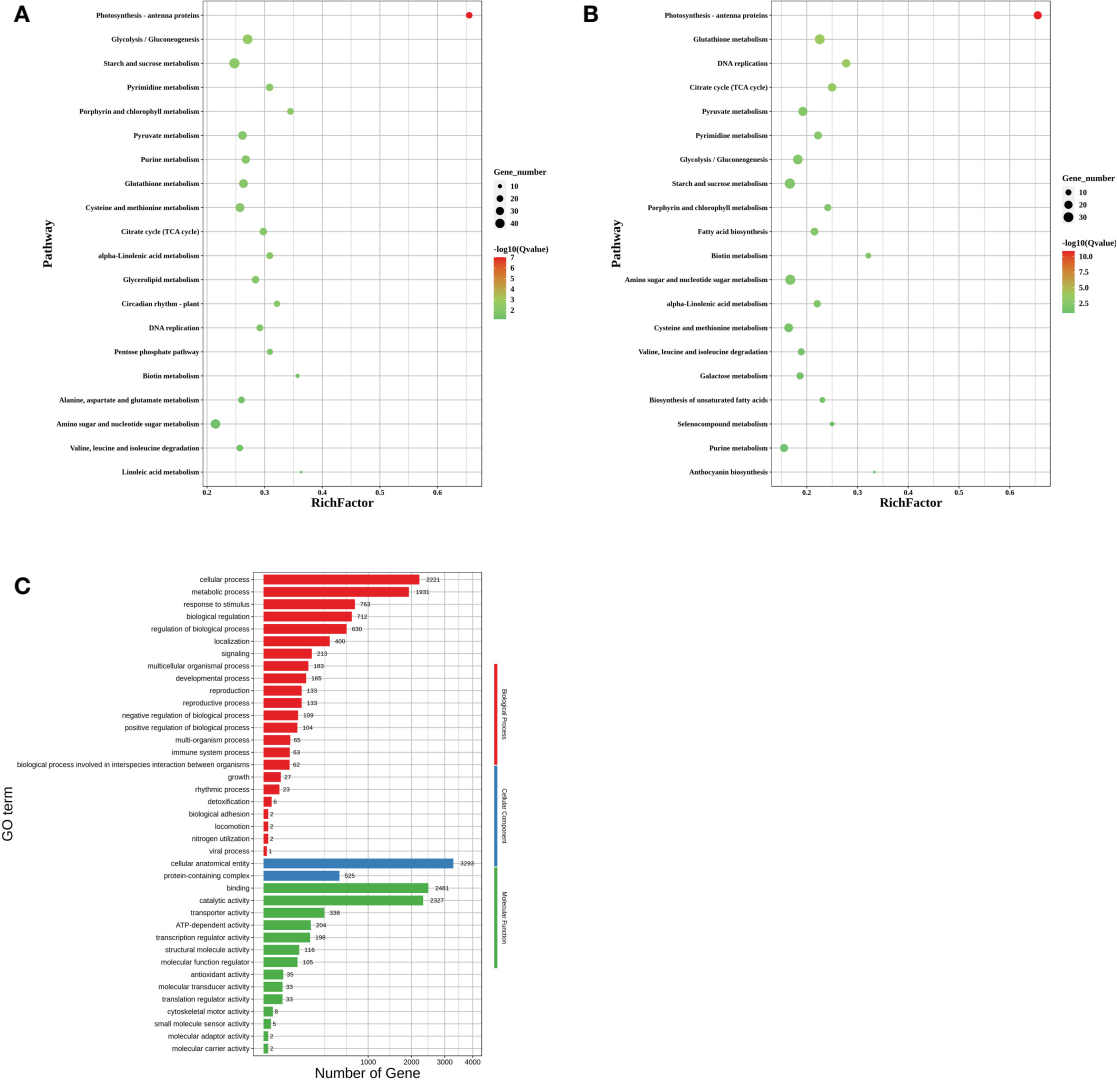
RNA-seq analysis of differentially expressed genes (DEGs) in *CA10g11690* silenced plants compared with the negative controls. **(A)** Kyoto Encyclopedia of Genes and Genomes (KEGG) pathway enrichment analysis of DEGs. **(B)** KEGG pathway enrichment analysis of downloaded DEGs. **(C)** Gene Ontology (GO) enrichment analysis of DEGs.

To further explore the effect of downregulation of *CA10g11690* on the expression of genes involved in anthocyanin biosynthetic metabolism and other biological processes, we performed a comparative transcriptome analysis in the exocarp of Chen12-4 and Chen12-4-VIGS690 lines at 15 DPA. A total of 5,979 differentially expressed genes (DEGs) were identified, and 3,055 upregulated and 2,924 downregulated genes in *CA10g11690*-silenced plants. The Kyoto Encyclopedia of Genes and Genomes (KEGG) pathway analysis was conducted to determine the effect of *CA10g11690* on the biological pathways of pepper. As a result, 126 pathways were found to be disturbed in *CA10g11690* silenced plants. As shown in **Figure 8A**, some important pathways were identified, which included pathways associated with photosynthesis-antenna proteins, carbohydrate metabolism and amino acid metabolism. We further focused on the 20 most significantly affected pathways for the Chen12-4 vs Chen12-4-VIGS690 downregulated DEGs. Among these pathways, the anthocyanin biosynthesis pathway was significantly enriched and four downregulated UDP-glycosyltransferase genes (*CA10g16530*, *CA10g16540*, *CA10g16550* and *CA10g16560*) was identified (**Figure 8B**), demonstrating that this term was evidently influenced by *CA10g11690*. DEGs were then annotated according to the Gene Ontology (GO) database and divided into three groups, including cellular component (CC), molecular function (MF), and biological process (BP). The 20 most significant GO terms, such as DNA packaging complex, nucleosome, protein-DNA complex, protein heterodimerization activity and chloroplast were enriched (**Figure 8C**), implying that these terms were influenced by *CA10g11690*.

We focused on the anthocyanin biosynthetic metabolism in *CA10g11690*-silenced lines. As shown in **Figure 7A**, the expression of the structural genes related to the anthocyanin biosynthetic pathway, including *CaCHS*, *CaF3H*, *CaF3’5’H*, *CaDFR*, *CaANS, CaUFGT, Ca3GT* and *Ca3RT*, in the purple striped exocarps of Chen12-4 was marked higher than that of the silencing induced Chen12-4-VIGS690 lines. However, the level of expression of the *CaCHI* gene was higher in the exocarp of Chen12-4-VIGS690 than in Chen12-4. The levels of expression of the anthocyanin regulatory genes *CA10g11690* (*CaMYB*), *CabHLH* and *CaWD40* were significantly downregulated in the Chen12-4-VIGS690 lines. The levels of expression of anthocyanin biosynthetic genes that were determined using a qRT-PCR analysis were consistent with the results of transcriptome (**Figure 7C**). The results indicated that *CA10g11690* participates in the formation of purple striped immature fruit in Chen12-4 by affecting the accumulation of anthocyanin in the exocarp.

### Overexpression of *CA10g11690* induces the accumulation of anthocyanin and upregulates the anthocyanin biosynthetic genes in *N. tabacum*

**Figure 9:**
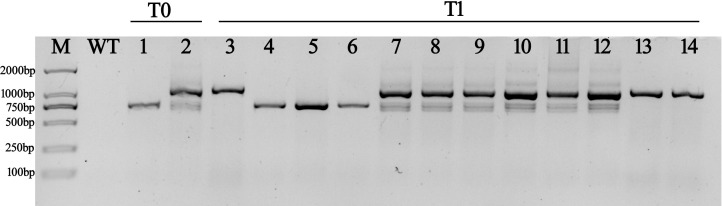
The RT-PCR products for the cDNA of the *CA10g11690* in transgenic tobacco lines. WT: control tobacco line, 1: *CaMV35S::c690^Short-Chen12-4^* T0-lines, 2: *CaMV35S::c690^Long-Z101^* T0-lines, 3: *CaMV35S::c690^Short-Chen12-4^* T1-lines with green phenotype, 4-6: *CaMV35S::c690^Short-Chen12-4^* T1-lines with purple phenotype, 7-12: *CaMV35S::c690 ^Long-Z101^* T1-lines with purple phenotype, 13-14: *CaMV35S::c690 ^Long-Z101^* T1-lines with green phenotype.

Transgenic *N. tabacum* lines that expressed pepper *c690^Long-Z101^* and *c690^Short-Chen12-4^* under the control of the CaMV35S promoter were generated using the pMV2 vector. As a result, both of the transgenic T0-lines, *CaMV35S::c690^Long-Z101^* and *CaMV35S::c690^Short-Chen12-4^* showed a deeper purple color (**Supplementary Figures 3A–H**). To further confirm the visual observation, we quantified the anthocyanin content of different transgenic tobacco lines. The anthocyanin contents of the *N. tabacum* transgenic overexpression lines were 18.93-23.94 mg·100 g^-1^ fresh weight, while those of the control plants were 1.39 mg·100 g^-1^ fresh weight (**Supplementary Figure 3I**). Thus, both *c690^Long-Z101^* and *c690^Short-Chen12-4^* with *CaMV35S* promoted the accumulation of anthocyanin and produced purple phenotypes. The *N. tabacum* overexpression transgenic lines with the best purple coloration were selected to generate T1 plants. We visually observed that the *CaMV35S::c690^Short-Chen12-4^* T1-lines were completely deep purple, while the *CaMV35S::c690^Long-Z101^* lines had a much lighter purple color (**Supplementary Figures 4A–F**). Quantitative of anthocyanin content showed that the *CaMV35S::c690^Short-Chen12-4^* lines were significantly higher than *CaMV35S::c690^Long-Z101^* lines (**Supplementary Figure 4G**). The transcription of *CA10g11690* was detected in more detail in several randomly selected transgenic lines (**Figure 9**). We detected the 702 bp amplicon in *CaMV35S::c690^Short-Chen12-4^*, and two amplicons with 702 bp and 1,087 bp in *CaMV35S::c690^Long-Z101^* in the T0 generation lines. Only the overexpressing *N. tabacum* transgenic lines with the same genotype as the T0 lines produced purple phenotypes in the T1 generation. It appeared that the transcript of *c690^Short-Chen12-4^* was more essential for the purple phenotype.

The patterns of expression of anthocyanin biosynthesis-related genes were explored in more detail in the leaves of transgenic tobacco T0-lines. The levels of expression of *CA10g11690* in the *CaMV35S*::*c690^Long-Z101^* lines and *CaMV35S*::*c690^Short-Chen12-4^* lines was 357.81- to 4,032.59-fold higher than those in the WT in the qRT-PCR analysis (**Supplementary Figure 5**). EBGs, including *NtCHS*, *NtCHI*, *NtF3H*, and *NtF3’5’H*, and LBGs, such as *NtDFR, NtANS, NtUFGT, Nt3GT*, and *Nt3RT*, were more highly expressed in the leaves of transgenic tobacco lines than in the WT. *NtbHLH* and *NtWD40* were also more highly expressed in the transgenic lines. The levels of expression of these EBGs, LBGs, and MBW transcription factors were correlated with the total anthocyanin content. The results further indicated that *CA10g11690* participates in the accumulation of anthocyanin, and the overexpression of *CA10g11690* led to upregulation of the genes involved in anthocyanin biosynthesis.

### *CaPs* is a novel allele variation of the *CaAN3* gene

A recent study reported a novel R2R3-MYB transcription factor, *CaAN3* (*Dem.v1.00043895*), that regulated fruit-specific anthocyanin accumulation in pepper (Byun et al., 2022). In this study, we identified the *CaPs* locus that was annotated as *CA10g11690*, *Dem.v1.00043895* in the Dempsey v1.0 reference genome. The action of *CaAN3* showed a phenotype of a fruit-specific and almost full purple color in fruit, such as the pepper inbred ZFJ in our study (**Supplementary Figure 6**). However, *CaPs* not only results in a purple striped phenotype in fruit but also purple anther filaments and styles in flowers (**Figure 1A**). Furthermore, we also identified many sequence variations of *CA10g11690* in the parental line Chen-14, including a large fragment insertion, indel and SNPs (**Figure 4**; **Supplementary Files 2**, **3**). Therefore, *CaPs* was the novel allele variation of the *CaAN3* gene.

### Promoter region variation in *CA10g11690* not affected its expression

To study the allele variation in more detail, we amplified and then sequenced the promoter region, genomic sequences and CDS of *CA10g11690* (*CaAN3*) in different pepper accessions (**Supplementary Figure 6**). The promoter regions of *CA10g11690* in M38, THIP, BZ, ZL, and S18 were completely identical to that of the green-fruit parent Z101-M, and a 1 bp insertion was found in ZFJ and Chen12-3 with the others (not include Chen12-4 and LJBN002). Interestingly, a substantial amount of sequence variation was found in the promoter region in LJBN002 with the *CaAN3* genotype. A total of 49 SNPs variations, two sequence deletions, and four sequence insertions were found between LJBN002 and Z101-M, and 53 SNP variations, two sequence deletions and three sequence insertions were found between LJBN002 and Chen12-4 (**Supplementary Figure 7A**; **Supplementary File 3**). In a previous study (Byun et al., 2022), the SCAR marker set was developed to analyze the relationship of structural variation of promoter region and expression of *CaAN3*. As expected, genotyping using the SCAR marker was completely consistent with the sequence of promoter region in our study. LJBN002 yielded a 524 bp amplicon, and Chen12-4 amplified 524 bp and 1,188 bp PCR products. Other lines showed a 1,188 bp amplicon (**Supplementary Figure 7B**). We further examined the expression of *CA10g11690* in these different accessions, and this gene was expressed in six purple peppers, including Chen12-4, ZFJ, LJBN002, THIP, BZ, and ZL (**Supplementary Figure 7C**). The phenotype of ZFJ, LJBN002 and ZL were fruit-specific anthocyanin accumulation, and confirmed to harbor *CaAN3* gene. While in THIP and BZ, the purple color was observed in various tissues, because of both *CaAN2* and *CaAN3* genotypes. Therefore, the sequence variation in promoter region was not affected the expression of *CA10g11690*, which was consistent the results of Byun et al. (2022).

### Alternative splicing affects the transcripts and expression of *CA10g11690* in pepper

**Figure 10:**
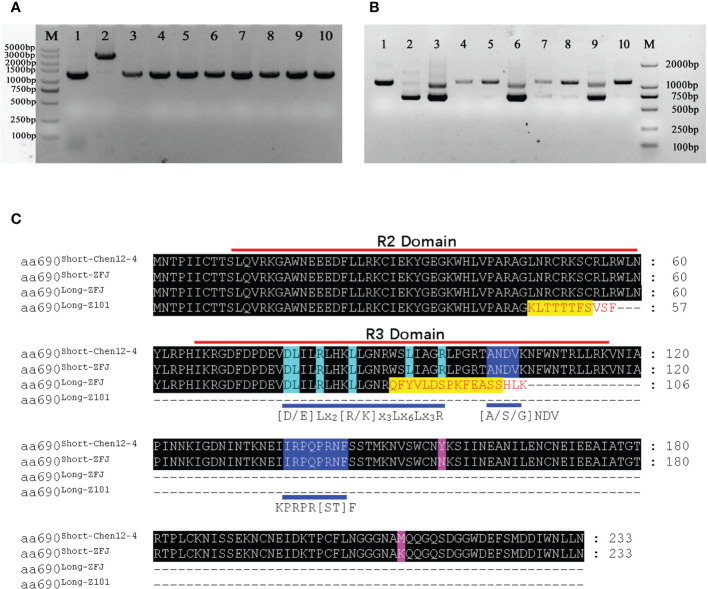
PCR amplified the gDNA and CDS from different pepper accessions and sequence alignment of different amino acid sequences. **(A)** PCR products for the gDNA of the *CA10g11690* in different pepper accessions. **(B)** RT-PCR products for the CDS of the *CA10g11690* in different pepper accessions. Lane 1-10: Z101-M, Chen12-4, ZFJ, Chen12-3, M38, LJBN002, THIP, BZ, ZL and S18. **(C)** Speculative amino acid sequences of transcripts in different pepper accessions.

We also amplified the gDNA and CDS regions of *CA10g11690* from these different pepper lines. The size of gDNA sequences was approximately 1,400 bp, and the insertion of large fragment was only found in the purple striped parental line Chen12-4 (**Figure 10A**). The amplification of CDSs showed alternative splicing in *CA10g11690* with different genotypes in pepper (**Figure 10B**). The green fruit pepper lines that did not harbor any functional genes involved in purple color and M38, the *CaAN2* genotype, could generate only one transcript *c690^Long-101^*. In contrast, five pepper lines with purple fruit, including ZFJ, LJBN002, THIP, BZ, and ZL, could generate two transcripts, *c690^Long^* and *c690^Short^*. Interestingly, the size of transcript *c690^Long^* from ZFJ, LJBN002 and ZL was shorter than the transcript for *c690^Long-Z101^*. These different transcripts were sequenced (**Supplementary File 2**). The spliced transcript *c690^Long^* in Chen12-3-5, M38, and THIP30 was identical to that of Z101-M, but there was a 72 bp deletion in ZFJ, LJBN002, and ZL. Thus, we designated the 1,015 bp transcript as *c690^Long-ZFJ^*. The length of all the *c690^Short^* transcripts was 702 bp, but there were two SNPs at the 466^th^ bp (T-A) and 632^th^ bp (T-A) sites in other accessions comparing with Chen12-4, that caused two amino acid substitutions. We designated the 702 bp transcript from ZFJ and other accessions as *c690^Short-ZFJ^.* We analyzed the ORFs that the different transcripts encoded in more detail. Transcript *c690^Long-ZFJ^* contained an ORF that encoded a deduced protein of 147 amino acids owing to the premature stop codon TAA. Thus, the *c690 ^Long-ZFJ^* transcript could encode a protein that contained an intact R2 domain but an incomplete R3 domain. In contrast, the *c690^Long-Z101^* transcript only encodes an incomplete R2 domain (**Figure 10C**). The *c690^Short^* transcript could encode the ORF that contained the conservative [D/E]Lx2[R/K]x3Lx6Lx3R motif in the R3 domain, which is a central feature for proteins that function by interacting with bHLH proteins (Zimmermann et al., 2004), and thus, participate in forming the MBW complexes to induce anthocyanin synthesis. We also found that the levels of expression of *CA10g11690* in these accessions revealed a correlation with transcript *c690^Short^*. Thus, alternative splicing affected the transcripts and expression of *CA10g11690* in pepper.

## Discussion

Anthocyanins are water-soluble specialised metabolites, which not only play diverse biological roles in plant life cycle, but also offer beneficial effects on human health. In solanaceous vegetables, such as tomato, a few genes that control anthocyanin accumulation have been cloned, and the transcriptional regulatory network for anthocyanin biosynthesis has been well-characterized (Cao et al., 2017; Colanero et al., 2018; Colanero et al., 2020; Sun et al., 2020; Yan et al., 2020). However, the alleles at *CaAn2* and *CaAn3* fail to fully explain all instances of purple pigmentation in pepper immature fruits. In this study, we identified a rare spontaneous mutant line, Chen12-4, with purple striped types of anthocyanins that accumulate in the exocarp of pepper fruit. The BSA-seq approach was utilized to perform gene mapping of the locus that controlled the formation of purple stripes in immature pepper fruit. It was demonstrated that *CaPs* was the novel allelic variation of the *CaAN3* gene responsible for the accumulation of anthocyanins in Chen12-4. Additionally, splicing mutations affect the transcripts and expression of *CaAN3* in pepper. Our study provides a useful reference for the future knowledge of anthocyanin biosynthesis in *Capsicum* species.

Anthocyanin biosynthetic pathway is mainly regulated by an MBW complex at the transcriptional level, and the R2R3-MYB transcription factors are the key element in determining activates or inhibits anthocyanin biosynthesis. The genes that encode the R2R3-MYB anthocyanin activators of solanaceous plants corresponds to the ortholog of petunia *PhAN2* (Payyavula et al., 2013; Docimo et al., 2015; Liu et al., 2018). In pepper, two main R2R3-MYB transcription factors, *CaAN2* and *CaAN3*, have been identified in this collinear region and are closely associated with the accumulation of anthocyanin (Borovsky et al., 2004; Jung et al., 2019; Byun et al., 2022). *CaAN2* regulates the anthocyanin biosynthesis in various tissues, including flowers, immature fruit, leaves, and stem, particularly when the plants are stressed by high light (Wang et al., 2022). Additionally, the *Fc* locus, the allelic gene of *CaAN2*, controls the purple anther filaments in pepper (Chaim et al., 2003). The latest reported *CaAN3*, is an activator of anthocyanin biosynthesis in immature pepper fruit, specifically (Byun et al., 2022). In this study, we identified a spontaneous mutant pepper line, Chen12-4, with the phenotype of purple stripes in unripe fruit and purple filaments and styles. However, *CaAN2* and *CaAN3* fail to fully explain this phenotype in Chen12-4. Therefore, we sought to identify genetic factors responsible for the anthocyanin biosynthetic gene from Chen12-4 using an F_2_ population obtained from the cross between lines Chen12-4 and Z101-M. The candidate region was delimited by the markers M-CA690-Xba and M-CA710-03 on chromosome 10, suggesting that *CA10g11690* is the best candidate gene for the *CaPs* locus. *CA10g11690* was expressed at a substantially higher level in purple striped mature unripe fruit than in green mature unripe fruit. We found that marker SCAR690-01, designed according to a 1,926 bp fragment insertion in Chen12-4, co-segregated with the purple striped phenotype in two F_2_ populations. In addition, we overexpressed it in *N. tabacum* leaves and VIGS in pepper to validate the role of *CA10g11690* as an activator of anthocyanin biosynthesis in pepper. This result confirmed that *CA10g11690* was the *CaPs* locus that controls the purple striped type of anthocyanin that accumulates in unripe pepper fruit.

The upregulated expression of MYBs has been considered to be a vital factor to produce anthocyanins in plants, and variation in the promoter region is the primary reason for different transcription levels of these MYBs (Albert et al., 2014; He et al., 2020a). For example, an insertion of Harbinger DNA transposon at the -373 bp of the promoter region of *Pr-D* (R2R3 MYB) activate the expression of anthocyanin related genes in the mutant purple cauliflower (*Brassica oleracea* var. *botrytis*) (Chiu et al., 2010). The multiple repeats in *MdMYB10* promoter segment results in transcriptional autoregulation and accumulation of anthocyanin that causes the red-fleshed and red-foliage phenotype in apple (*Malus domestica*) (Espley et al., 2007). Similarly, in pepper, a Ca-nLTR-A retrotransposon insertion in the upstream regulatory region of *CaAN2* is responsible for the purple phenotype in various tissues (Borovsky et al., 2004; Jung et al., 2019; Ohno et al., 2020). Thus, the promoter activity of *MYBs* could be the major force for the novel variation in purple traits, and potential differences could be distributed in the *cis*-regulatory regions or even epigenetic changes (Walker et al., 2007; Chiu et al., 2010; He et al., 2020a). However, the accumulation of fruit-specific anthocyanins is not caused by the variation in the upstream regulatory of *CaAN3*, because pepper accessions with green phenotype in immature fruit also harbor the functional *CaAN3* allele (Byun et al., 2022). In our study, the sequencing analysis of promoter variation of *CaAN3* in different pepper lines further supported this conclusion. Sequence variation in the promoter region does not affect the expression of *CA10g11690.*

As is well-known, the R2R3-MYB is the key regulators in the MBW complex that primarily determines the anthocyanin accumulation. The expression of R2R3-MYB over a certain threshold to be able to recruit enough bHLH and WDR partners to constitute the MBW complex to drive anthocyanin production (Colanero et al., 2020). In *Aft* tomato, the level of expression of *SlAN2like^Aft^* is high enough to activate the biosynthesis of anthocyanins when exposed to light. In WT tomato, the SlAN2like protein is non-functional, and another R2R3-MYB TF, *SlAN2*, cannot reach the threshold to recruit its bHLH partner SlAN1 to form the MBW complex. Overexpression of two MYB genes, *SlANT1* or *SlAN2*, resulted in the purple phenotype and an upregulation of the anthocyanin biosynthetic genes, including EBGs, LBGs, and bHLH-encoding gene *SlAN1* (Kiferle et al., 2015; Meng et al., 2015). In our study, the 35S promoter drives the overexpression of *CA10g11690*, including the *c690^Long-Z101^*, *c690^Long-ZFJ^*, *c690^Short-Chen12-4^* and *c690^Short-ZFJ^* transcripts, and induced significantly upregulation of the anthocyanin related genes and caused anthocyanin accumulation in *N. tabacum* transgenic lines (**Supplementary Figures 8**, **9**). Additionally, the high upregulation of *F3H, DFR, ANS, 3GT* and *3RT* in both transgenic tobacco lines and purple striped pepper strongly indicates that *CA10g11690* as an activator to drive the biosynthesis of anthocyanin in tobacco and pepper, respectively.

A large number of alternative splicing events has become identified in plants, and more than 60% of the intron-containing genes are estimated to undergo alternative splicing in *Arabidopsis* (Syed et al., 2012). Several studies have shown that the alternative splicing of anthocyanin related genes affects the accumulation of anthocyanin pigments in horticultural plants. For example, the splicing variant of *CmbHLH2* leads to a truncated protein that could not interact with *CmMYB6* and ultimately blocks the accumulation of colored pigments in white florets in *Chrysanthemum* (Lim et al., 2021). In tomato, splicing mutations of *SlAN2-like*, which is anthocyanin-promoting regulator, determine the production of a dysfunctional protein (Colanero et al., 2020). The alternative splicing of *CaAN3* was also found to affect anthocyanin biosynthesis in our study. *CaAN3* produced two different transcripts, *c690^Long-Z101^* and *c690^Short-Chen12-4^*, in two parents. A comparison of the structure of two different transcripts showed that the loss of most of the amino acid residues downstream of the R2 domain and the complete absence of the R3 domain in c690^Long-Z101^ prevents it from forming the MBW complex. In contrast, the *c690^Short-Chen12-4^* transcript from the purple striped parent, contains the conservative motif of [D/E]Lx2[R/K]x3Lx6Lx3R in the R3 domain, which is responsible for interaction with bHLH proteins (Zimmermann et al., 2004), and thus, participate in forming the MBW complexes to induce anthocyanin synthesis. Moreover, we detected another transcript, *c690^Long-ZFJ^*, in the purple pepper lines ZFJ, LJBN002 and ZL. We speculated that *c690^Long-ZFJ^* might not be a functional transcript involves the biosynthesis of anthocyanin in pepper. Although *c690^Long-ZFJ^* has an intact R2 domain, a rearrangement of [D/E]Lx2[R/K]x3Lx6Lx3R motif that contains the bHLH-binding site and the loss of [A/S/G]NDV aminoacidic signature of R2R3 MYBs that promotes anthocyanin, results in the inability to associate with the bHLH partners. We also hypothesized that *c690^Long-Z101^* might not be the functional transcript that affects the biosynthesis of anthocyanin in pepper. Because only one transcript, *c690^Long-Z101^*, could be amplified in pepper lines with unfunctional *CaAN3* allele. Moreover, we could not confirm the *c690^Long-ZFJ^* transcript as functional. Both *c690^Long-ZFJ^* and *c690^Short^* transcripts were amplified in pepper lines that carried the functional *CaAN3* allele, but purple-fruit peppers carrying unique *c690^Long-ZFJ^* transcript was not screened. However, the 702bp-*c690^Short^* must be functional because both the purple pepper lines (*CaAN3*-expressed) and purple *N. tabacum* transgenic lines can be amplified to produce the *c690^Short^* transcript in our study (**Figures 9**, **10B**). Additional yeast two-hybrid assays will be conducted to analyse the interaction of c690^Long-Z101^ and c690^Long-ZFJ^ with the CabHLH factor. Although the individual functions of *CA10g11690* involving anthocyanin biosynthesis have been characterized, the underlying transcriptional network is still unclear in pepper. We observed that the level of expression of *CA10g11690* was higher in the *CaMV35S* driven *c690^Long^* than in the *c690^Short^* transgenic tobacco lines, but the structural genes were lower in the *CaMV35S* driven *c690^Long^* transgenic tobacco lines. Since *c690^Short^* is the key functional transcript that affects anthocyanin synthesis with a deeper purple color, this could explain why the level of expression of structural genes is higher in the *c690^Short^* transgenic tobacco lines.

Introns regulate gene expression in plants and many other eukaryotes has been demonstrated in various ways, such as positive regulatory and inhibitory effects. For example, BrMYB2 promotes anthocyanin biosynthesis under the control of the short intron 1 of gBrMYB2, but represses with a long intron 1 (He et al., 2020b). The mutant rice (*Oryza sativa*) plants that harbor a T-DNA insertion in the first intron of chlorophyll a oxygenase (*OsCAO1*) cause the pale-green leaves (Lee et al., 2005). In our study, an insertion fragment of 1,926 bp in the second intron region of the purple striped parental line Chen12-4 was detected. The purple striped spontaneous mutant was derived from a hybrid combination designated XianjiaoF_1_-No.12 (named Chen12-F_1_). Therefore, the different inbred lines with green and purple striped phenotypes were selected from Chen12-F_1_. We found that the insertion fragment of 1,926 bp in the second intron region was specific in purple inbred lines. We hypothesized that the insertion in intron of *CA10g11690* in Chen12-4 could be the reason for the purple striped phenotype. However, the hypothesis merits additional verification by a transgenic complementation test.

## Conclusions

In this study, we identified the *CaPs* locus that is involved in the formation of purple stripes on the unripe fruit of pepper. The molecular marker that co-segregated with the *CaPs* locus was developed for molecular marker-assisted selection. This study provides a good insight into the mechanism underlying anthocyanin biosynthesis and facilitates the future breeding of purple striped varieties of pepper.

## Data availability statement

The original contributions presented in the study are publicly available. This data can be found here: https://www.ncbi.nlm.nih.gov/bioproject/PRJNA926138.

## Author contributions

NL and YL conceived and conducted the research, and wrote the manuscript with important contribution. YY and SG collaborated in the phenotyping of the accessions, and developed and evaluated plant material. FYW performed the genotype analysis of population. CY performed the transgenic tobacco. BK revised the manuscript. FW, CJ and MY conducted the project administration. All authors contributed to the article and approved the submitted version.
