# Supplementary material for: Identification of CaPs locus involving in purple stripe formation on unripe fruit, reveals allelic variation and alternative splicing of R2R3-MYB transcription factor in pepper (Capsicum annuum L.)
Source: Front Plant Sci. 2023 Mar 28;14:1140851. doi: 10.3389/fpls.2023.1140851 (PMC10089288; doi:10.3389/fpls.2023.1140851)
Supplement: Supplementary file 1 [file DataSheet_1.pdf]

Supplementary Material

Identification of *CaPs* locus involving in purple stripe formation on unripe fruit, reveals allelic variation and alternative splicing of R2R3-MYB transcription factor in pepper (*Capsicum annuum L.*)

Ning Li [1,†], Yabo Liu [1,2,†], Yanxu Yin [1], Shenghua Gao [1], Fangyuan Wu [1], Chuying Yu [1], Fei Wang [1*], Byoung-Cheorl Kang [3], Kai Xu [1], Chunhai Jiao [1], Minghua Yao [1,4*]

*** Correspondence:**

Fei Wang
20966954@qq.com

Minghua Yao
yaomh_2008@126.com

†These authors contributed equally to this work and share first authorship

This PDF file includes:

Supplementary Figure 1 to 9

Supplementary Table 1

Supplementary File 1 to 3

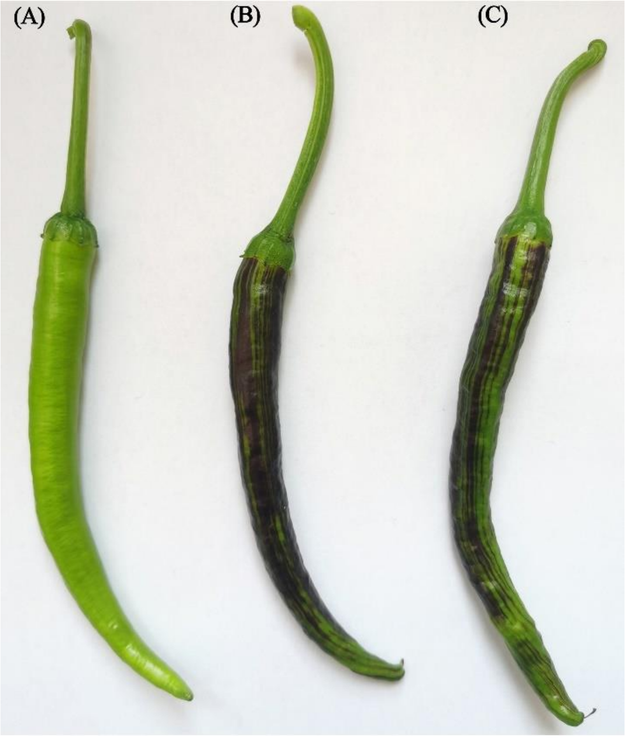

Supplementary Figure 1. Phenotypes of the fruits of two parents and F_1. **(A)** Fruit of Z101-M, **(B)** Fruit of Chen12-4, **(C)** Fruit of F_1.

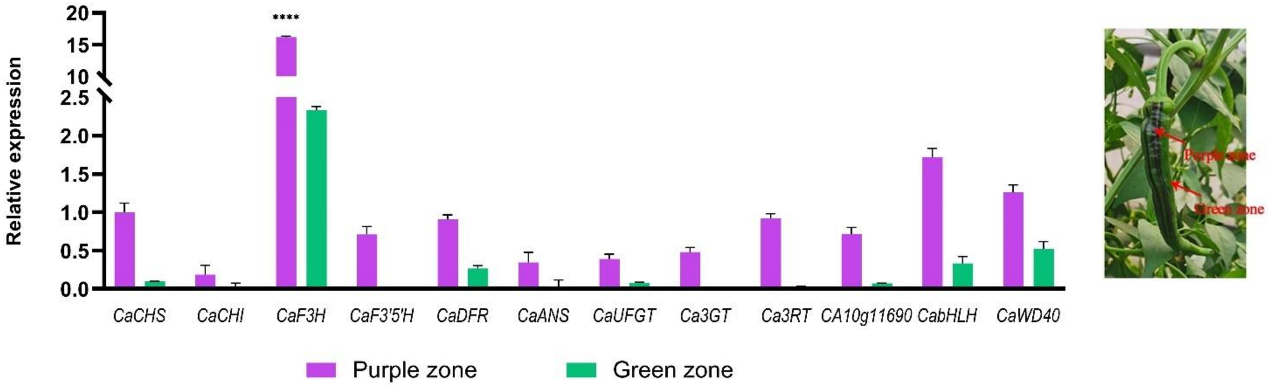

Supplementary Figure 2. qRT-PCR expression of anthocyanin biosynthesis structural genes and MBW transcription factor in purple zone compared with green zone in the exocarp of Chen12-4 at 15 DPA.

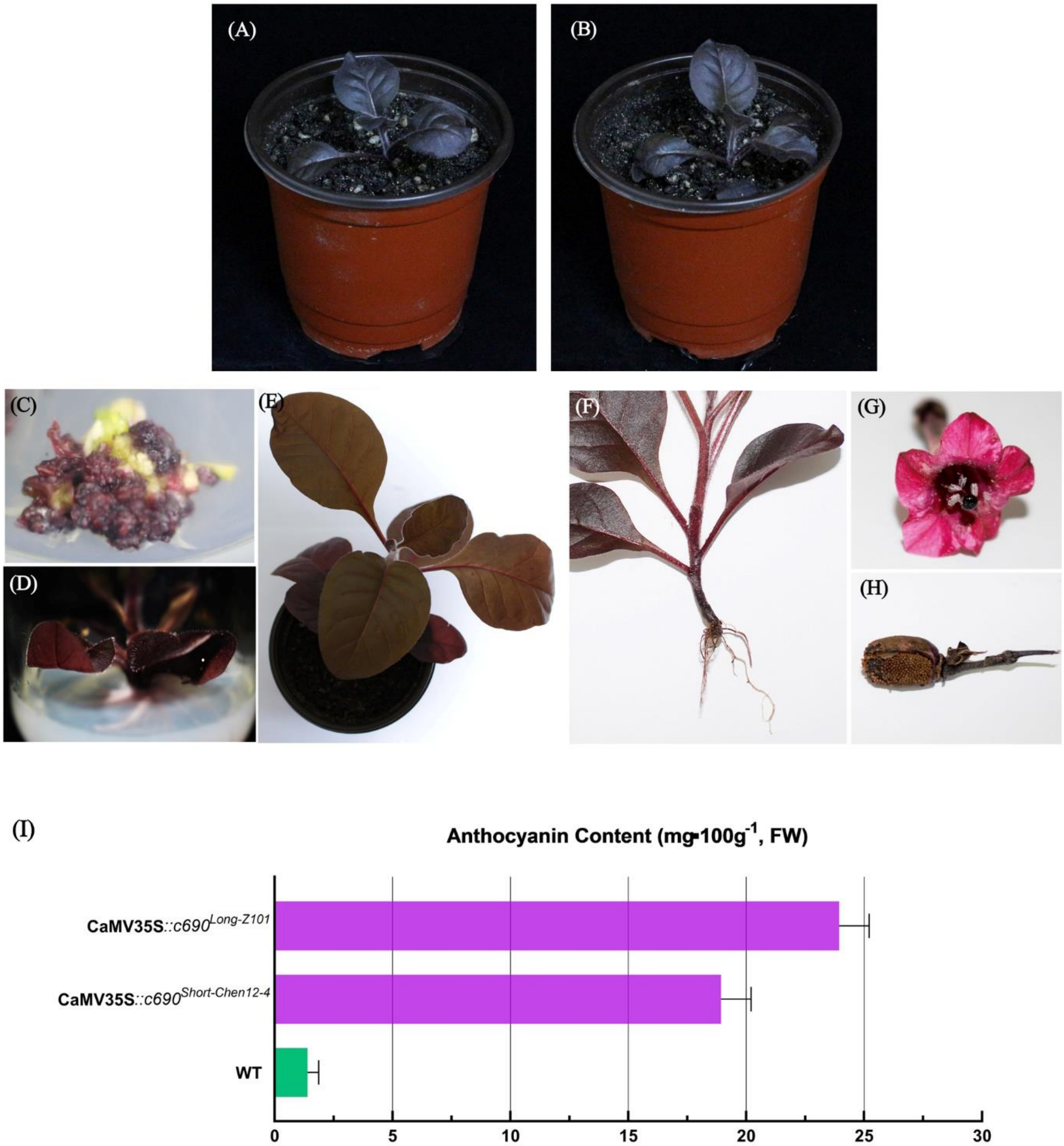

Samples	Control	CaMV35S::*c690*$^{Long\text{-}Z101}$	CaMV35S::*c690*$^{Short\text{-}Chen12\text{-}4}$
Anthocyanin Content (mg▪100g^{-1}, FW)	1.39±0.48	23.94±1.28	18.93±1.27

Supplementary Figure 3. Phenotype and anthocyanin contents of *c690*$^{Long\text{-}Z101}$ and *c690*$^{Short\text{-}Chen12\text{-}4}$ transgenic tobacco T0 lines under control of the *CaMV35S*. **(A)** The *CaMV35S::c690*$^{Long\text{-}Z101}$ transgenic tobacco T0-line, **(B)** The *CaMV35S::c690*$^{Short\text{-}Chen12\text{-}4}$ transgenic tobacco T0-line, **(C)** callus tissue, **(D)** transformant in culture medium, **(E)** transgenic tobacco plant, **(F)** transgenic tobacco plant with purple root, **(G)** the flower of transgenic tobacco plant, **(H)** the seeds of transgenic tobacco plant. **(I)** Anthocyanin contents of transgenic tobacco T0-lines and the control tobacco lines.

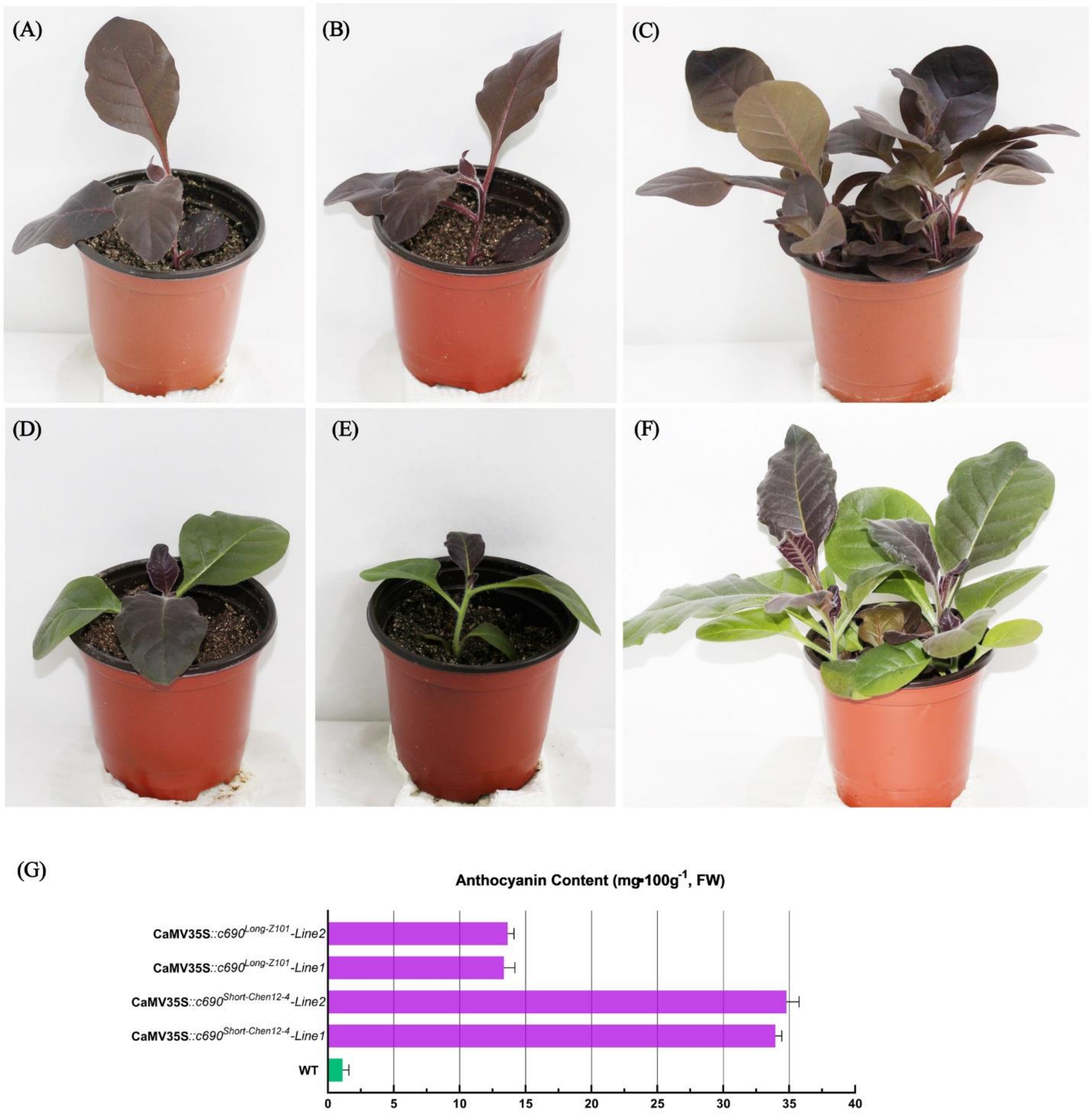

<table>
<tr><th>Samples</th><th>Control</th><th>CaMV35S::c690$^{Long-Z101}$-Line1</th><th>CaMV35S::c690$^{Short-Chen12-4}$-Line1</th></tr>
<tr><td rowspan="3">Anthocyanin Content (mg•100g^{-1}, FW)</td><td rowspan="3">1.11±0.48</td><td>13.36±0.83</td><td>33.95±0.48</td></tr>
<tr><td>CaMV35S::c690$^{Long-Z101}$-Line2</td><td>CaMV35S::c690$^{Short-Chen12-4}$-Line2</td></tr>
<tr><td>13.64±0.83</td><td>34.79±0.96</td></tr>
</table>

Supplementary Figure 4. Phenotype and anthocyanin content of different T1 generation transgenic tobacco lines. **(A)-(C)** The *CaMV35S::c690*$^{Long-Z101}$ transgenic tobacco T1-lines, **(D)-(F)** The *CaMV35S::c690*$^{Short-Chen12-4}$ transgenic tobacco T1-lines, **(G)** Anthocyanin contents of transgenic tobacco T1-lines and the control tobacco lines.

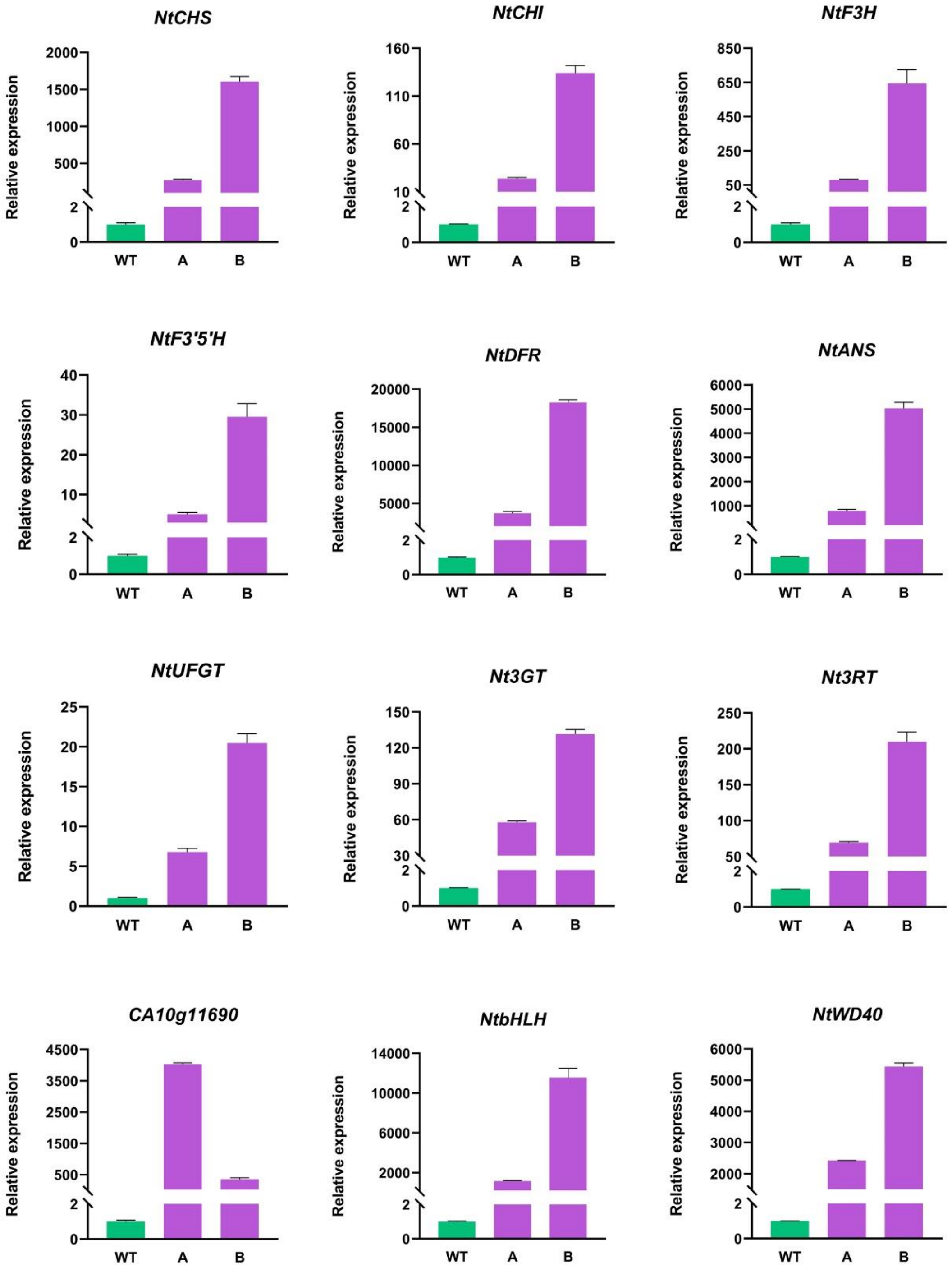

Supplementary Figure 5. qRT-PCR analysis of structural genes and regulatory gene in anthocyanin biosynthetic pathway in transgenic tobacco T0-lines. **WT**, control tobacco line; **A**, The *CaMV35S::c690*$^{Long\text{-}Z101}$ transgenic tobacco T0-lines; **B**, The *CaMV35S::c690*$^{Short\text{-}Chen12\text{-}4}$ transgenic tobacco T0-lines.

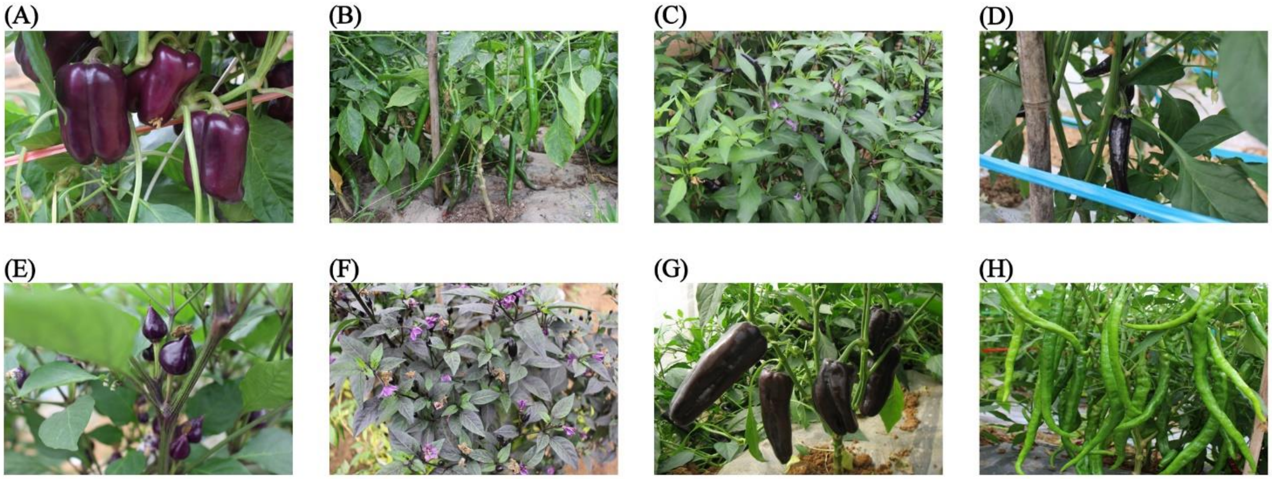

Supplementary Figure 6. Pepper accessions with different phenotype and genotype. **(A)** ZFJ, *CaAN3* genotype, fruit-specific purple color. **(B)** Chen12-3-5, non-functional *CaPs* allele, sister line of Chen12-4 with normal green fruit; Chen12-3 for short. **(C)** M38, *CaAN2* genotype, purple in various tissues; **(D)** LJBN002-X, *CaAN3* genotype, fruit-specific purple color; LJBN002 for short. **(E)** THIP30-X-3-1, *CaAN2* and other genotypes, purple color in various tissues; THIP for short. **(F)** BZ01-X1-X1-1, *CaAN2* and other genotypes, purple color in various tissues; BZ for short. **(G)** ZiLong-F_8-1, *CaAN3* genotype, fruit-specific purple color; ZL for short. **(H)** S18-M, normal green-fruit, not carrying any functional allele involving purple color; S18 for short. The genotype of these pepper lines has been identified using the molecular markers A_SCAR of *CaAN2* and HRM 10-63 of *CaAN3*, which were developed by Jung (2019).

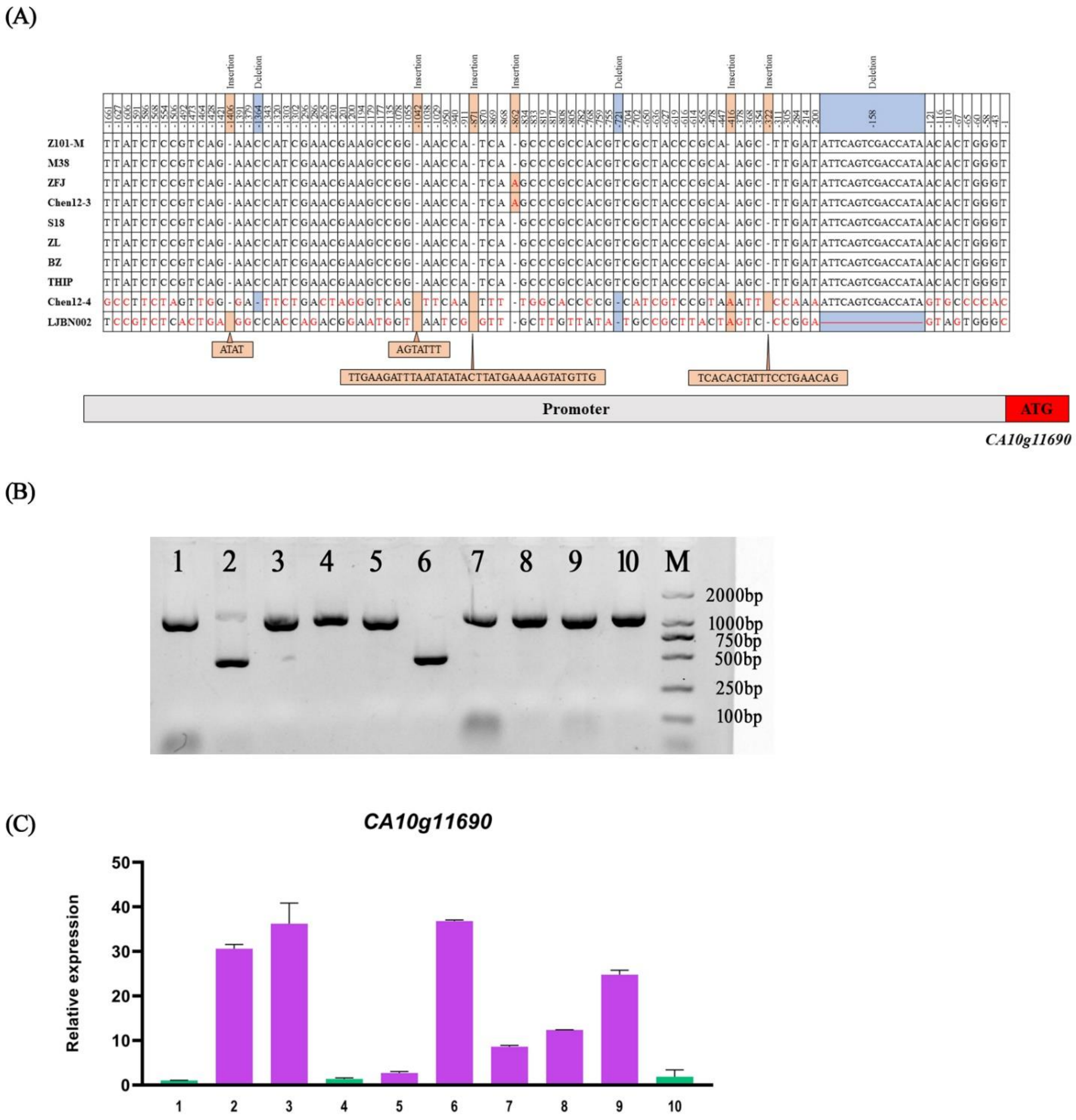

Supplementary Figure 7. Analysis the promoter region of *CA10g11690* in pepper with different genotype and phenotype. **(A)** Sequence variation analysis in the promoter region of *CA10g11690* in different pepper accessions. **(B)** Genotyping of structural variation in the promoter region of *CA10g11690* in different accessions. **(C)** Expression of *CA10g11690* in different accessions. 1-10: Z101-M, Chen12-4, ZFJ, Chen12-3, M38, LJBN002, THIP, BZ, ZL and S18.

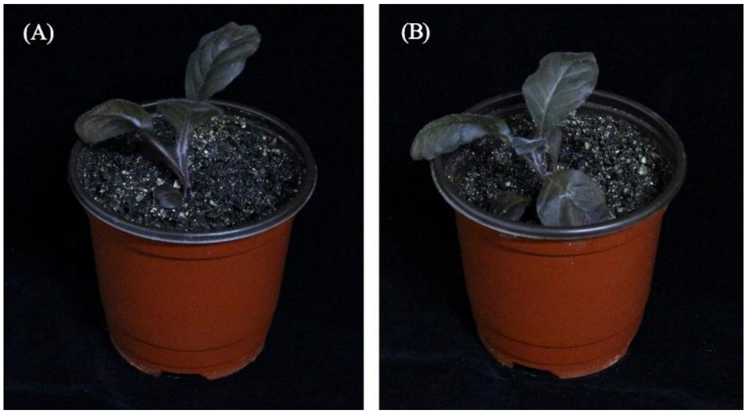

Supplementary Figure 8. Phenotype of the transgenic tobacco lines. **(A)** The *CaMV35S::c690*$^{Long\text{-}ZFJ}$ transgenic tobacco line. **(B)** The *CaMV35S::c690*$^{Short\text{-}ZFJ}$ transgenic tobacco line

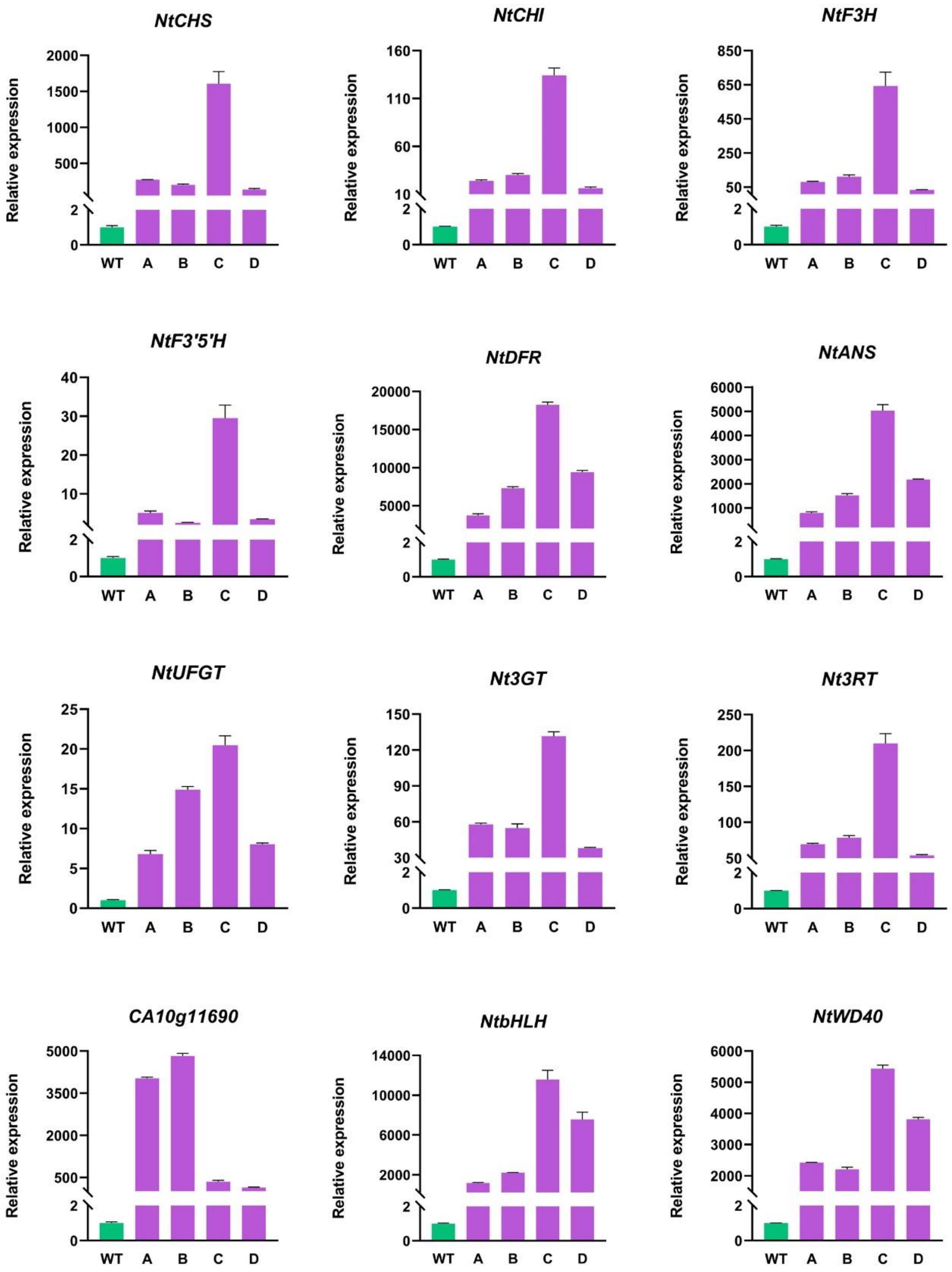

Supplementary Figure 9. qRT-PCR analysis of structural genes and regulatory gene in anthocyanin biosynthetic pathway in different transgenic tobacco T0-lines. **WT**, control tobacco line; **A**, The *CaMV35S::c690*$^{Long\text{-}Z101}$ transgenic tobacco T0-lines; **B**, The *CaMV35S::c690*$^{Long\text{-}ZFJ}$ transgenic tobacco T0-lines; **C**, The *CaMV35S::c690*$^{Short\text{-}Chen12\text{-}4}$ transgenic tobacco T0-lines; **D**, The *CaMV35S::c690*$^{Short\text{-}ZFJ}$ transgenic tobacco T0-lines

Supplementary Table 1. Primers and markers used in the present study.

Primer names	Primer sequence 5'→3'	Purpose	Restriction enzyme	SNP position (CM334 Ver1.55)	Reference
M02F	AGTCCCTCACAATACCGACA	mapping	TaqI	12,146,566	
M02R	GGCCTTCCTTCTGTTTAAAGTCT				
M03F	GCAAACGGAATATGTGACCCT	mapping	TaqI	24,714,179	
M03R	TTTCACCGTTAGAGCTTCACT				
M04F	TCGCCAAGAAGTCGCAAAAT	mapping	TaqI	35,979,777	
M04R	CGGGTCGTAGGTGCCTATAC				
M05F	AGAGACAACATGCCCCAAAA	mapping	TaqI	48,848,179	
M05R	TCAGCCCACTCTCTCATCTC				
M06F	ATGGCGCCCGTTACTCTATT	mapping	TaqI	61,954,759	
M06R	GGAACAGCGTACAGTCTAACC				
M07F	CATCTCTTGCACCAATCCATGA	mapping	TaqI	71,204,775	
M07R	AAGTGAGGTGCGTCATTTCG				
M08F	GTGGCCTTGGTTTGTTCCTT	mapping	TaqI	81,068,860	
M08R	TGTCGTACCTTCATTGCACG				
M09F	ACTAATGCACGCAAACCCAG	mapping	TaqI	99,002,901	
M09R	TGTGTTTTGAGTTCCTGAGTGT				
M10F	TTGTGACCATAAGCCCCGTT	mapping	TaqI	113,760,213	
M10R	AAGGATGGGCTGCGATAAGT				
M11F	TTTGCTTTCTGACGCCACAA	mapping	TaqI	125,480,118	
M11R	TATGTAGGCGGGTGTTGAGC				
M12F	AGCCTCTGGACGGTATCATG	mapping	TaqI	137,631,003	
M12R	GCGAGTAGATTTGTTCATGATCG				
M13F	CATCTATGCGGGGTAAGGGA	mapping	TaqI	151,201,437	
M13R	AGCTGGTTGGGAAGGTATGT				
M14F	TGTGTCCCATGCAGAGACTT	mapping	TaqI	159,226,939	
M14R	CCCAACTACAGTCCTTGCCT				
M15F	GGCGTAATGACTGTGGAGAA	mapping	TaqI	174,751,143	
M15R	TTTCCTACCACACCAACCCT				
M16F	AGACACTATCCATACTGCTCCA	mapping	TaqI	188,371,164	
M16R	TTCCGTGTATGCTGAGGTCC				
M17F	AGTGGGTTTTGAGGGGATGA	mapping	TaqI	198,884,784	
M17R	TGTTGGTACCGGTAGAATATTGG				
M18F	TGGTTCTTGTTTGATCTCCCC	mapping	TaqI	209,241,036	
M18R	AATGGACCGGATTTAGCGGA				
M19F	AACAGCTCACCCGATCTCAT	mapping	TaqI	219,626,523	
M19R	ACAGCATCAAACTCAAGGCA				
M20F	GGGATCACTTTCATGTCCTGC	mapping	TaqI	230,830,255	
M20R	AAGATGCGTTACTTGAGTGGT				
M182-08F	ACTGTGCATATGGTTACGTGAC	fine mapping	TaqI	181,820,331	
M182-08R	AACCGTTGCTCAATCTTCCC				
M182-01AF	TGGCGAGGGAATGCATATTG	fine mapping	Csp6I	182,113,171	
M182-01AR	ACAATTCCTGACCAGCCGTA				
M16-182AF	AGACCTGCCAGATCTGAAGA	fine mapping	TaqI	182,145,711	
M16-182AR	TGCCCTTTGTTCATAAGTTTTGA				
M182-06F	TGTGACCGCAATCAAGACAC	fine mapping	Csp6I	182,301,501	
M182-06R	AGGTTAAGGGTGTTCAGCCA				
M-CA650-03F	ACACTGACCACTGACCATGT	fine mapping	Csp6I	182,958,624	
M-CA650-03R	CCACACCAAACTTCAGGGATG				
M-CA660-04F	ATCCCCAATTACCACCCCAG	fine mapping	HpaII	183,428,486	
M-CA660-04R	TGTGGGGCGAGTTTATGGAG				
CAPS690-01F	TGGATTCGTCGAACTCACTCAA	fine mapping	TaqI	183,613,393	
CAPS690-01R	ACGCAGTGAAGGGTATGGTC				
M16-184AF	AATTTGGGGTGTGGTTTGCA	fine mapping	TaqI	183,709,825	
M16-184AR	GCAATTGTTGGGTTTGGTGC				
M-CA690-XbaF	ACCCTCACAAGCATTGGACT	fine mapping	XbaI	184,002,197	

M-CA690-XbaR	ACTACTTTTCAGATCTGGGGTGA				
M-CA690-01F	GAGATTGGTTCGTTGATGGC	fine mapping	TaqI	184,358,410	
M-CA690-01R	AAACCCGTTTGCACAAGAAAA				
M-CA690-02F	ATCAAACCTGGTCATTCGCG	fine mapping	BcuI	184,365,148	
M-CA690-02R	GCTCTGTTTAGGGGTTGCAC				
M-CA710-03F	GTGTGCTTGCCTTTACATTAAGT	fine mapping	TaqI	184,843,586	
M-CA710-03R	AAATGTTACACGTACGAAGCAC				
M15-10F	ACCAGAGCATTATCTAGTGCAAGT	fine mapping	TaqI	184,966,141	
M15-10R	CGGGGTTTTCCTCATCAGGTT				
q650F	ACGTACTAAGACCTCGCCCT	qRT-PCR of *CA10g11650*			
q650R	TGTCGCGTTCGTCTTTGTCT				
q660F	GGAAGAACAACGAACGATGTGAA	qRT-PCR of *CA10g11660*			
q660R	TAATGGCTTTGCCCTTGTTGT				
q690F	CAATGAAATTGAAGAAGCAATAGCA	qRT-PCR of *CA10g11690*			
q690R	TTTCATTGCAATTTTTCTCAGAGC				
q710F	TCGTGGTGCAACAACAAAAGT	qRT-PCR of *CA10g11710*			
q710R	TCGACGACGTTGTTTCGACT				
qCaUBI3F	GTCCATCTGCTCTCTGTTG	qRT-PCR			
qCaUBI3R	CACCCCAAGCACAATAAGAC				
qCaCHS-F	GTGGAACCGTTATCCGACTAGCAA	qRT-PCR			Jung, 2019
qCaCHS-R	CCTCCTGGTTCTACACCACC				
qCaCHI-F	ACGCTGATCATCAAGCAGTG	qRT-PCR			Jung, 2019
qCaCHI-R	GTGGAACCGTTATCCGACTAGCAA				
qCaF3H-F	CCTCCTGGTTCTACACCACC	qRT-PCR			Jung, 2019
qCaF3H-R	ACGCTGATCATCAAGCAGTG				
qCaF3'5'H-F	GATGGGGTGGCCGGTGATTG	qRT-PCR			Zhang et al., 2015
qCaF3'5'H-R	GCCACCACAACGCGCTCG				
qCaDFR-F	CAAGGCAGAGGGAAGATTCA	qRT-PCR			Jung, 2019
qCaDFR-R	CAAATGCCCACAACCAGAACTAGC				
qCaANS-F	CCTGAGAGTCATGTACATGGAGG	qRT-PCR			Jung, 2019
qCaANS-R	CAAGGCAGAGGGAAGATTCA				
qCaUFGT-F	CAAATGCCCACAACCAGAACTAGC	qRT-PCR			Jung, 2019
qCaUFGT-R	CCTGAGAGTCATGTACATGGAGG				
qCabHLH-F	CAATGGAGCTATAAAGACTAGGAA	qRT-PCR			Zhang et al., 2015
qCabHLH-R	GGAAAAGAGAAAGAAACACACATG				
qCaWD40-F	TTCCAACAAGTTGCAGCTGC	qRT-PCR			Liu et al., 2020
qCaWD40-R	AGCTCCAACCCCTTTTCCAC				
qCa3GT-F	CGGATCCCCAAACCAAGAAAACATCAAG	qRT-PCR			Liu et al., 2020
qCa3GT-R	CGGATCCCGAAATTAGTTGGAGTTG				
qCa3RT-F	GGCTGATGAGTTAGGGATCAAG	qRT-PCR			
qCa3RT-R	GGAACTCTAGCAGGACAAGTAAG				
qNtCHS-F	TGACACCCACTTGGATAGTTTAG	qRT-PCR			Liu et al., 2019
qNtCHS-R	CGACCTCTGGAATTGGATCAG				
qNtCHI-F	CTTTTCTCGCCGCTAAATG	qRT-PCR			Liu et al., 2019
qNtCHI-R	TTTCTGCCACCTTCTCTG				
qNtF3H-F	CAAGGCATGTGTGGATATGG	qRT-PCR			Liu et al., 2019
qNtF3H-R	TGTGTCGTTTCAGTCCAAGG				
qNtF3'H-F	AGGCTCAACACTTCTCGT	qRT-PCR			Liu et al., 2019
qNtF3'H-R	CATCAACTTTGGGCTTCT				
qNtF3'5'H-F	CGCACTACCATACTTAGGAGCCAT	qRT-PCR			Liu et al., 2019
qNtF3'5'H-R	CAGCATCAGGAGTAGAAGCAACAG				
qNtDFR-F	AACCAACAGTCAGGGGAATG	qRT-PCR			Liu et al., 2019
qNtDFR-R	TTGGACATCGACAGTTCCAG				
qNtANS-F	TGGCGTTGAAGCTCATACTG	qRT-PCR			Liu et al., 2019
qNtANS-R	GGAATTAGGCACACACTTTGC				
qNtUFGT-F	GAGTGCATTGGATGCCTTTT	qRT-PCR			Liu et al., 2019
qNtUFGT-R	CCAGCTCCATTAGGTCCTTG				
qNtAN2-F	GAAGAAAGGTGCATGGACTG	qRT-PCR			Liu et al., 2019
qNtAN2-R	TCTGCAGCTCTTTCTGCATC				

qNtbHLH-F	ACCATTCTCGAACACCGAAG	qRT-PCR			Lim et al., 2017
qNtbHLH-R	TGCTAGGGCACAATGTGAAG				
qNtWD40-F	ATGCGATTGCGTGGGCTCCACA	qRT-PCR			
qNtWD40-R	CAAGCAATCCAAAAGGGTTGCA				
qNtActin-F	GGTTGGATCTTGCTGGTCGT	qRT-PCR			Zhong et al., 2020
qNtActin-R	GCAGTCTCCAACTCTTGCTCA				
cds-690F	GTGAGAGTGTGTGCGCATATATAGA	CDS analysis			
cds-690R	GACCGTCAACTTCTCAAGACATTAC				
gDNA-690F	GTGGCACCTTGTTCCTGCTA	full-length gene analysis			
gDNA-690R	TAGAGCTTTTGACCCCGGCT				
Pro-690F	CACGGGGAAGTTGGATCTTTTG	promoter analysis			
Pro-690R	TTAGAGCTTTTGACCCCGGCT				
TRV2-V690F	CTAGATGAGGAGAAGAGCCC TACTCGCCTTCTGAGGAAGGTA	pTRV2-specific primers			
TRV2-V690R	TGTGCTCGACGACAAGACCC AGGTGTTCTAGTTCCAGTTGCT				
OE-690F	GTGAGAGTGTGTGCGCATATATAGA	pMV2-specific primers			
OE-690R	GACCGTCAACTTCTCAAGACATTAC				

Supplementary File 1. Nucleotide alignment of gDNA sequences of *CA10g11690* in two parents. g690-Chen12-4, gDNA of *CA10g11690* in Chen12-4. g690-Z101, gDNA sequence of *CA10g11690* in Z101-M.

```
g690-Chen12-4   ATGAATACTCCAATAATCTGTACAACATCGTTGCAAGTAAGGAAAGGTGCATGGAATGAA :   60
g690-Z101       ATGAATACTCCAATAATCTGTACAACATCGTTGCAAGTAAGGAAAGGTGCATGGAATGAA :   60
Consensus       ATGAATACTCCAATAATCTGTACAACATCGTTGCAAGTAAGGAAAGGTGCATGGAATGAA

g690-Chen12-4   GAAGAAGATTTTCTTTTGAGAAAATGCATTGAAAAATATGGTGAAGGAAAGTGGCACCTT :  120
g690-Z101       GAAGAAGATTTTCTTTTGAGAAAATGCATTGAAAAATATGGTGAAGGAAAGTGGCACCTT :  120
Consensus       GAAGAAGATTTTCTTTTGAGAAAATGCATTGAAAAATATGGTGAAGGAAAGTGGCACCTT

g690-Chen12-4   GTTCCTGCTAGAGCTGGTAAACTAACCACTACTACTTTCTCCGTCTCATTTTAACGAGTT :  180
g690-Z101       GTTCCTGCTAGAGCTGGTAAACTAACCACTACTACTTTCTCCGTCTCATTTTAACGAGTT :  180
Consensus       GTTCCTGCTAGAGCTGGTAAACTAACCACTACTACTTTCTCCGTCTCATTTTAACGAGTT

g690-Chen12-4   TCAAAAGTCTTTATTTCTTTATGTGCAGGTCTAAATAGATGTCGGAAGAGCTGCAGACTT :  240
g690-Z101       TCAAAAGTCTTTATTTCTTTATGTGCAGGTCTAAATAGATGTCGGAAGAGCTGCAGACTT :  240
Consensus       TCAAAAGTCTTTATTTCTTTATGTGCAGGTCTAAATAGATGTCGGAAGAGCTGCAGACTT

g690-Chen12-4   CGGTGGTTGAATTATCTGAGGCCACATATCAAGAGAGGTGACTTCGATCCAGATGAAGTG :  300
g690-Z101       CGGTGGTTGAATTATCTGAGGCCACATATCAAGAGAGGTGACTTCGATCCAGATGAAGTG :  300
Consensus       CGGTGGTTGAATTATCTGAGGCCACATATCAAGAGAGGTGACTTCGATCCAGATGAAGTG

g690-Chen12-4   GATCTCATTTTGAGGCTTCATAAGCTCTTAGGAAACAGGCAATTTTATGTTTTAGATTCA :  360
g690-Z101       GATCTCATTTTGAGGCTTCATAAGCTCTTAGGAAACAGGCAATTTTATGTTTTAGATTCA :  360
Consensus       GATCTCATTTTGAGGCTTCATAAGCTCTTAGGAAACAGGCAATTTTATGTTTTAGATTCA

g690-Chen12-4   CCTAAATTTGCGGGATCATCTCATTTGAAAGTTAATGATATTAGAGATCTAGAGTACAAT :  420
g690-Z101       CCTAAATTTGAGGCATCATCTCATTTGAAAGTTAATGATATTAGAGATCTAGAGTACAAT :  420
Consensus       CCTAAATTTG GG ATCATCTCATTTGAAAGTTAATGATATTAGAGATCTAGAGTACAAT

g690-Chen12-4   TCTTATTACTATATAATTATGCCTCAACATGCTGGCCAATTGGTGCACTGTGCGCAGGCA :  480
g690-Z101       TCTTATTACTATATAATTATGCCTCAACATGCTGGCCAATTGGTGCACTGTGCGCAGGCA :  480
Consensus       TCTTATTACTATATAATTATGCCTCAACATGCTGGCCAATTGGTGCACTGTGCGCAGGCA

g690-Chen12-4   TTTATAATTCTGTTATACATGAGTAGAAGCACATAAAAATATTTCATTGCTACTTTTTTT :  540
g690-Z101       TTTATAATTCTGTTATACATGAGTAGAAGCACATAAAAATATTTCATTGCTACTTTTTTT :  540
Consensus       TTTATAATTCTGTTATACATGAGTAGAAGCACATAAAAATATTTCATTGCTACTTTTTTT

g690-Chen12-4   TCATGGCACTTTAGACCGAATTTGCACTTTAATTTCTCTCAAATTTACACTTTAATTGCT :  600
g690-Z101       GCATG------------------------------------------------------- :  545
Consensus        CATG

g690-Chen12-4   TCAATTTGGGAATTTCTATGACTTTGACCTAATTTTTAGGGATTTTGGGTTTATCTTCTC :  660
g690-Z101       ------------------------------------------------------------ :    -
Consensus

g690-Chen12-4   CTTAGAGAACATTTGATTGCTGGGCTCATTTTATTGCGTGAAATTTTTTATTCTTCCTTG :  720
g690-Z101       ------------------------------------------------------------ :    -
Consensus

g690-Chen12-4   TAATTCTGCCGATTACAGTCGTTATGTTGGGAAAAATATTGTAATGTGTTATATTGACAA :  780
g690-Z101       ------------------------------------------------------------ :    -
Consensus

g690-Chen12-4   GAGAAAGTTGAGGAACATTAGCGTTACTGATTAGAGTAACTGTAGTAGTTGGGCTAACTG :  840
g690-Z101       ------------------------------------------------------------ :    -
Consensus
```

```
g690-Chen12-4   CAGTAGATTTTAGCATTTTTAAGGGTGTTCTTGTTTATATTGATGTGCAGATTAATTGGT :  900
g690-Z101       ------------------------------------------------------------ :    -
Consensus

g690-Chen12-4   GTTCCATTGGTTGATTTGTATTGTTATTATTTTCTTATTTTACAATTTAGAACCTGATAA :  960
g690-Z101       ------------------------------------------------------------ :    -
Consensus

g690-Chen12-4   GAATCATTTCGTTCATCATTTCACGATTCACTTATATTAGCAATAAAGATGTTCTTACAT : 1020
g690-Z101       ------------------------------------------------------------ :    -
Consensus

g690-Chen12-4   GTTGAAGTTGTTGTTCTTCACTATATATCTCCAGACACGCCTTTGAACAAGCAGGGAGAG : 1080
g690-Z101       ------------------------------------------------------------ :    -
Consensus

g690-Chen12-4   ACCTAGGCTACATATTTACCCAAGGAAAAGACATTGATATTGAGAAGATTAATCAATATA : 1140
g690-Z101       ------------------------------------------------------------ :    -
Consensus

g690-Chen12-4   AGTACTTTCTTTCTCTTTTTCAGCTACATGAAGGCACTGCTTAAGGTGAACAGGTGTGTG : 1200
g690-Z101       ------------------------------------------------------------ :    -
Consensus

g690-Chen12-4   AACTAAAGAATTTATTTATTTCCCTTTTCTTTTCTGTCACCAGCGCTGAAAATCCTATCT : 1260
g690-Z101       ------------------------------------------------------------ :    -
Consensus

g690-Chen12-4   ACGTGCTTGTTCATTTTTCTTTGGTTTTCCAACTTTATTCAAGTTGCTTCACATTTCTTG : 1320
g690-Z101       ------------------------------------------------------------ :    -
Consensus

g690-Chen12-4   TGAATAAAGAGCGATCATTTTGAGATAGAAGAAGTCATTTGAAGCGTATTATCTTGCAGG : 1380
g690-Z101       ------------------------------------------------------------ :    -
Consensus

g690-Chen12-4   GAGATCAATTAAACAGATATAACAATATGGGTAGCAGGACCTTGAAACTTGGAATGTAAG : 1440
g690-Z101       ------------------------------------------------------------ :    -
Consensus

g690-Chen12-4   TTTAATGACGTAAGGCGGGAGAATGAGGTAGTAGTGAGGCTAGAAGCACAGGAGGTAGAG : 1500
g690-Z101       ------------------------------------------------------------ :    -
Consensus

g690-Chen12-4   AAAAGGGATAAGTTCAAGTATCTCGGGTCCGTGATCCAGAGTAACGGTGAGATTGACGAG : 1560
g690-Z101       ------------------------------------------------------------ :    -
Consensus

g690-Chen12-4   GATGTCTCGCACCGTATTGGGGCGGGATGGATGAAGTGGAAACTTGCATCGGGGGTGCTG : 1620
g690-Z101       ------------------------------------------------------------ :    -
Consensus

g690-Chen12-4   TGTGATAAGAAGGTGCCGCCCAAGCTTAAAGGTAAATTCTATAGGGTGGTAGTCCGTCCG : 1680
g690-Z101       ------------------------------------------------------------ :    -
Consensus

g690-Chen12-4   GCCTTGCTGTATGGAGTGGAGTGTTGGCCAGTTAAGAACTCCCACATCCAAAGAATGAAG : 1740
g690-Z101       ------------------------------------------------------------ :    -
Consensus

g690-Chen12-4   TGGCAGAAATGCGGATGTTGCGCTGGATGTGTGGACTGACCCGAAGGGATAGAGCTCGG : 1800
g690-Z101       ------------------------------------------------------------ :    -
Consensus
```

```
g690-Chen12-4   AATGAGACTATCCGGGAGAAGGTTGGTGTGACTTCAGTGGAGTGTAAGATGCGGGAAGCA : 1860
g690-Z101       ------------------------------------------------------------ :    -
Consensus

g690-Chen12-4   CGATTGAGATGGTTCGGACACGTGAAGAGGAGGGGCATGGATGCCCCGGTCCGTAGGTGT : 1920
g690-Z101       ------------------------------------------------------------ :    -
Consensus

g690-Chen12-4   GAGAGGCTAGCGTTGGATGGTTTTAGACGGGGTAGGGGTAGACCGAAGAAGTACTGGGGT : 1980
g690-Z101       ------------------------------------------------------------ :    -
Consensus

g690-Chen12-4   GAGGTGATTAGGCGGGACATGGAACAGTTACAGCTTACCGAGGACATGACCCTAGATAGG : 2040
g690-Z101       ------------------------------------------------------------ :    -
Consensus

g690-Chen12-4   AAGGTCTGGAAGACGCGAATTACGGCAGAGGATTAGGGCCAGTTCGGGTCGCTAATGTAG : 2100
g690-Z101       ------------------------------------------------------------ :    -
Consensus

g690-Chen12-4   GGAAGTAATTGGTGGGGGTGTATTCCTGTTATGATTCCGTATTCAATGTTCCGTGTTCCG : 2160
g690-Z101       ------------------------------------------------------------ :    -
Consensus

g690-Chen12-4   TGTTCCATGTTTGTTATGAATCTGTGTGCTTTCCTCTGCTTTATATTCCTGCATTCCTGC : 2220
g690-Z101       ------------------------------------------------------------ :    -
Consensus

g690-Chen12-4   TTTACTCTGTTTTATATTCCTTATGGCTGCAGTATCTATGTTATGTCATCTGCTTCTGTG : 2280
g690-Z101       ------------------------------------------------------------ :    -
Consensus

g690-Chen12-4   CTGTACTATGTGTTTGTGTGATATCTCGTATCTCGTAACTCGTAACTTGAGCCGGGGGTC : 2340
g690-Z101       ------------------------------------------------------------ :    -
Consensus

g690-Chen12-4   TTTCGGAAACAGCCTTTCTACTTCATCAGAGGTAGAGGTATGGACTGCGTACATCTTACC : 2400
g690-Z101       ------------------------------------------------------------ :    -
Consensus

g690-Chen12-4   CCCCCAGACCCCACTAAGTGGGAATACACTGGGTTTGTTGTTGTTGTTGTTGTTGTTACT : 2460
g690-Z101       ------------------------------------------------------------ :    -
Consensus

g690-Chen12-4   TTTTTTTCATGGCAGTATAATTTTAAATTCACGAGATCATGAAATTTTGACATCATATTA : 2520
g690-Z101       -----------GCAGTATAATTTTAAATTCACGAGATCATGAAGTTTTGACATCATATTA :  594
Consensus                  GCAGTATAATTTTAAATTCACGAGATCATGAA TTTTGACATCATATTA

g690-Chen12-4   ATTGAATACATGTGATCATTTCATTTAAAACTTACGCAAATTATGTTATATATGTAGATG : 2580
g690-Z101       ATTGAATACGTGTGATCATTTCATTTAAAACTTACGCAAATTATGTTATATATGTAGATG :  654
Consensus       ATTGAATAC TGTGATCATTTCATTTAAAACTTACGCAAATTATGTTATATATGTAGATG

g690-Chen12-4   GTCACTTATTGCTGGTAGACTTCCGGGAAGGACAGCGAACGATGTGAAGAATTTCTGGAA : 2640
g690-Z101       GTCACTTATTGCTGGTAGACTTCCGGGAAGGACAGCGAACGATGTGAAGAATTTCTGGAA :  714
Consensus       GTCACTTATTGCTGGTAGACTTCCGGGAAGGACAGCGAACGATGTGAAGAATTTCTGGAA

g690-Chen12-4   TACTCGCCTTCTGAGGAAGGTAAATATTGCTCCGATTAACAATAAGATCGGAGACAATAT : 2700
g690-Z101       TACTCGCCTTCTGAGGAAGGTAAATATTGCTCCGATTAACAATAAGATCGGAGACAATAT :  774
Consensus       TACTCGCCTTCTGAGGAAGGTAAATATTGCTCCGATTAACAATAAGATCGGAGACAATAT

g690-Chen12-4   TAATACTAAGAATGAGATAATAAGACCTCAACCTCGGAACTTCTCAAGTACCATGAAGAA : 2760
g690-Z101       TAATACTAAGAATGAGATAATAAGACCTCAACCTCGGAACTTCTCAAGTACCATGAAGAA :  834
Consensus       TAATACTAAGAATGAGATAATAAGACCTCAACCTCGGAACTTCTCAAGTACCATGAAGAA
```

```
g690-Chen12-4   TGTTTCTTGGTGCAACTACAAAAGTATCATAAATGAAGCAAATATACTGGAAAATTGCAA : 2820
g690-Z101       TGTTTCTTGGTGCAACAACAAAAGTATCATAAATGAAGCAAATATACTGGAAAATTGCAA :  894
Consensus       TGTTTCTTGGTGCAAC ACAAAAGTATCATAAATGAAGCAAATATACTGGAAAATTGCAA

g690-Chen12-4   TGAAATTGAAGAAGCAATAGCAACTGGAACTAGAACACCTTTATGCAAGAATATCAGCTC : 2880
g690-Z101       TGAAATTGAAGAAGCAATAGCAACTGGAACTAGAACACCTTTATGCAAGAATATCAGCTC :  954
Consensus       TGAAATTGAAGAAGCAATAGCAACTGGAACTAGAACACCTTTATGCAAGAATATCAGCTC

g690-Chen12-4   TGAGAAAAAATTGCAATGAAATTGATAAAACACCATGTTTTTTAAATGGTGGAGGCAACGC : 2940
g690-Z101       TGAGAAAAAATTGCAATGAAATTGATAAAACACCATGTTTTTTAAATGGTGGAGGCAACGC : 1014
Consensus       TGAGAAAAAATTGCAATGAAATTGATAAAACACCATGTTTTTTAAATGGTGGAGGCAACGC

g690-Chen12-4   CATGCAACAAGGACAAAGTGATGGTGGTTGGGATGAATTTTCTATGGATGACATATGGAA : 3000
g690-Z101       CAAGCAACAAGGACAAAGTGATGGTGGTTGGGATGAATTTTCTATGGATGACATATGGAA : 1074
Consensus       CA GCAACAAGGACAAAGTGATGGTGGTTGGGATGAATTTTCTATGGATGACATATGGAA

g690-Chen12-4   TCTACTTAATTAGCGGGTAATGTCTTGAGAAGTTGACGGTCTTGAACTTTATCAACCTCG : 3060
g690-Z101       TCTACTTAATTAGCGGGTAATGTCTTGAGAAGTTGACGGTCTTGAACTTTATCAACCTCG : 1134
Consensus       TCTACTTAATTAGCGGGTAATGTCTTGAGAAGTTGACGGTCTTGAACTTTATCAACCTCG

g690-Chen12-4   CTTGTCTTATGGACAAAACTTCAAATTTAACATCTTAATTGTTATCTTGATGATTTGCCA : 3120
g690-Z101       CTTGTCTTATGGACAAAACTTCAAATTTAACGTCTTAATTGTTATCTTGATGATTTGCCA : 1194
Consensus       CTTGTCTTATGGACAAAACTTCAAATTTAAC TCTTAATTGTTATCTTGATGATTTGCCA

g690-Chen12-4   ATGAAGCAAGCCGGGGTCAAAAGCTCTAA : 3149
g690-Z101       ATGAAGCAAGCCGGGGTCAAAAGCTCTAA : 1223
Consensus       ATGAAGCAAGCCGGGGTCAAAAGCTCTAA
```

Supplementary File 2. Nucleotide and amino acid sequences of *CA10g11690* in two parents and ZFJ.

>*c690*^{Long}-Z101 1,087bp
ATGAATACTCCAATAATCTGTACAACATCGTTGCAAGTAAGGAAAGGTGCATGGAATGAAGAAGAAGATTTTCTTTTGAGAAAATGCATT
GAAAAATATGGTGAAGGAAAGTGGCACCTTGTTCCTGCTAGAGCT**GGTAAACTAACCACTACTACTTTCTCCGTCTCATTTTAACGAGTT**
TCAAAAGTCTTTATTTCTTTATGTGCAGGTCTAAATAGATGTCGGAAGAGCTGCAGACTTCGGTGGTTGAATTATCTGAGGCCACATATC
AAGAGAGGTGACTTCGATCCAGATGAAGTGGATCTCATTTTGAGGCTTCATAAGCTCTTAGGAAACAGGCAATTTTATGTTTTAGATTCA
CCTAAATTTGAGGCATCATCTCATTTGAAAGTTAATGATATTAGAGATCTAGAGTACAATTCTTATTACTATATAATTATGCCTCAACAT
GCTGGCCAATTGGTGCACTGTGCGCAGGCATTTATAATTCTGTTATACATGAGTAGAAGCACATAAAAATATTTCATTGCTACTTTTTTT
GCATGGCAGTATAATTTTAAATTCACGAGATCATGAAGTTTTGACATCATATTAATTGAATACGTGTGATCATTTCATTTAAAACTTACG
CAAATTATGTTATATATGTAGATGGTCACTTATTGCTGGTAGACTTCCGGGAAGGACAGCGAACGATGTGAAGAATTTCTGGAATACTCG
CCTTCTGAGGAAGGTAAATATTGCTCCGATTAACAATAAGATCGGAGACAATATTAATACTAAGAATGAGATAATAAGACCTCAACCTCG
GAACTTCTCAAGTACCATGAAGAATGTTTCTTGGTGCAACAACAAAAGTATCATAAATGAAGCAAATATACTGGAAAATTGCAATGAAAT
TGAAGAAGCAATAGCAACTGGAACTAGAACACCTTTATGCAAGAATATCAGCTCTGAGAAAAATTGCAATGAAATTGATAAAACACCATG
TTTTTTAAATGGTGGAGGCAACGCCAAGCAACAAGGACAAAGTGATGGTGGTTGGGATGAATTTTCTATGGATGACATATGGAATCTACT
TAATTAG

>aa690^{Long}-Z101
MNTPIICTTSLQVRKGAWNEEEDFLLRKCIEKYGEGKWHLVPARAGKLTTTTFSVSF*

>*c690*^{Long}-ZFJ 1,015bp
ATGAATACTCCAATAATCTGTACAACATCGTTGCAAGTAAGGAAAGGTGCATGGAATGAAGAAGAAGATTTTCTTTTGAGAAAATGCATT
GAAAAATATGGTGAAGGAAAGTGGCACCTTGTTCCTGCTAGAGCTGGTCTAAATAGATGTCGGAAGAGCTGCAGACTTCGGTGGTTGAAT
TATCTGAGGCCACATATCAAGAGAGGTGACTTCGATCCAGATGAAGTGGATCTCATTTTGAGGCTTCATAAGCTCTTAGGAAACAGGCAA
TTTTATGTTTTAGATTCACCTAAATTTGAGGCATCATCTCATTTGAAAGTTAATGATATTAGAGATCTAGAGTACAATTCTTATTACTAT
ATAATTATGCCTCAACATGCTGGCCAATTGGTGCACTGTGCGCAGGCATTTATAATTCTGTTATACATGAGTAGAAGCACATAAAAATAT
TTCATTGCTACTTTTTTTGCATGGCAGTATAATTTTAAATTCACGAGATCATGAAGTTTTGACATCATATTAATTGAATACGTGTGATCA
TTTCATTTAAAACTTACGCAAATTATGTTATATATGTAGATGGTCACTTATTGCTGGTAGACTTCCGGGAAGGACAGCGAACGATGTGAA
GAATTTCTGGAATACTCGCCTTCTGAGGAAGGTAAATATTGCTCCGATTAACAATAAGATCGGAGACAATATTAATACTAAGAATGAGAT
AATAAGACCTCAACCTCGGAACTTCTCAAGTACCATGAAGAATGTTTCTTGGTGCAACAACAAAAGTATCATAAATGAAGCAAATATACT
GGAAAATTGCAATGAAATTGAAGAAGCAATAGCAACTGGAACTAGAACACCTTTATGCAAGAATATCAGCTCTGAGAAAAATTGCAATGA
AATTGATAAAACACCATGTTTTTTAAATGGTGGAGGCAACGCCAAGCAACAAGGACAAAGTGATGGTGGTTGGGATGAATTTTCTATGGA
TGACATATGGAATCTACTTAATTAG

>aa690^{Long}-ZFJ
MNTPIICTTSLQVRKGAWNEEEDFLLRKCIEKYGEGKWHLVPARAGLNRCRKSCRLRWLNYLRPHIKRGDFDPDEVDLILRLHKLLGNRQ
FYVLDSPKFEASSHLKVNDIRDLEYNSYYYIIMPQHAGQLVHCAQAFIILLYMSRST*

>*c690*^{Short}-Chen12-4 702bp
ATGAATACTCCAATAATCTGTACAACATCGTTGCAAGTAAGGAAAGGTGCATGGAATGAAGAAGAAGATTTTCTTTTGAGAAAATGCATT
GAAAAATATGGTGAAGGAAAGTGGCACCTTGTTCCTGCTAGAGCTGGTCTAAATAGATGTCGGAAGAGCTGCAGACTTCGGTGGTTGAAT
TATCTGAGGCCACATATCAAGAGAGGTGACTTCGATCCAGATGAAGTGGATCTCATTTTGAGGCTTCATAAGCTCTTAGGAAACAGATGG
TCACTTATTGCTGGTAGACTTCCGGGAAGGACAGCGAACGATGTGAAGAATTTCTGGAATACTCGCCTTCTGAGGAAGGTAAATATTGCT
CCGATTAACAATAAGATCGGAGACAATATTAATACTAAGAATGAGATAATAAGACCTCAACCTCGGAACTTCTCAAGTACCATGAAGAAT
GTTTCTTGGTGCAACTACAAAAGTATCATAAATGAAGCAAATATACTGGAAAATTGCAATGAAATTGAAGAAGCAATAGCAACTGGAACT
AGAACACCTTTATGCAAGAATATCAGCTCTGAGAAAAATTGCAATGAAATTGATAAAACACCATGTTTTTTAAATGGTGGAGGCAACGCC
ATGCAACAAGGACAAAGTGATGGTGGTTGGGATGAATTTTCTATGGATGACATATGGAATCTACTTAATTAG

>aa690^{Short}-Chen12-4
MNTPIICTTSLQVRKGAWNEEEDFLLRKCIEKYGEGKWHLVPARAGLNRCRKSCRLRWLNYLRPHIKRGDFDPDEVDLILRLHKLLGNRW
SLIAGRLPGRTANDVKNFWNTRLLRKVNIAPINNKIGDNINTKNEIIRPQPRNFSSTMKNVSWCNYKSIINEANILENCNEIEEAIATGT
RTPLCKNISSEKNCNEIDKTPCFLNGGGNAMQQGQSDGGWDEFSMDDIWNLLN*

>*c690*^{Short}-ZFJ 702bp
ATGAATACTCCAATAATCTGTACAACATCGTTGCAAGTAAGGAAAGGTGCATGGAATGAAGAAGAAGATTTTCTTTTGAGAAAATGCATT
GAAAAATATGGTGAAGGAAAGTGGCACCTTGTTCCTGCTAGAGCTGGTCTAAATAGATGTCGGAAGAGCTGCAGACTTCGGTGGTTGAAT
TATCTGAGGCCACATATCAAGAGAGGTGACTTCGATCCAGATGAAGTGGATCTCATTTTGAGGCTTCATAAGCTCTTAGGAAACAGATGG

TCACTTATTGCTGGTAGACTTCCGGGAAGGACAGCGAACGATGTGAAGAATTTCTGGAATACTCGCCTTCTGAGGAAGGTAAATATTGCT
CCGATTAACAATAAGATCGGAGACAATATTAATACTAAGAATGAGATAATAAGACCTCAACCTCGGAACTTCTCAAGTACCATGAAGAAT
GTTTCTTGGTGCAACAACAAAAGTATCATAAATGAAGCAAATATACTGGAAAATTGCAATGAAATTGAAGAAGCAATAGCAACTGGAACT
AGAACACCTTTATGCAAGAATATCAGCTCTGAGAAAAATTGCAATGAAATTGATAAAACACCATGTTTTTTAAATGGTGGAGGCAACGCC
AAGCAACAAGGACAAAGTGATGGTGGTTGGGATGAATTTTCTATGGATGACATATGGAATCTACTTAATTAG

>aa690^{Short}-ZFJ
MNTPIICTTSLQVRKGAWNEEEDFLLRKCIEKYGEGKWHLVPARAGLNRCRKSCRLRWLNYLRPHIKRGDFDPDEVDLILRLHKLLGNRW
SLIAGRLPGRTANDVKNFWNTRLLRKVNIAPINNKIGDNINTKNEIIRPQPRNFSSTMKNVSWCNNKSIINEANILENCNEIEEAIATGT
RTPLCKNISSEKNCNEIDKTPCFLNGGGNAKQQGQSDGGWDEFSMDDIWNLLN*

Supplementary File 3. The promoter region sequences of *CA10g11690* in Chen12-4, Z101-M and other pepper lines.

```
Promoter-Z101       GTCTCAAGTCTAAAGGGGCCAACTAATATAAAACGGAGGGAGTATTTAAGAGAAAATAAT :  60
Promoter-M38        GTCTCAAGTCTAAAGGGGCCAACTAATATAAAACGGAGGGAGTATTTAAGAGAAAATAAT :  60
Promoter-ZFJ        GTCTCAAGTCTAAAGGGGCCAACTAATATAAAACGGAGGGAGTATTTAAGAGAAAATAAT :  60
Promoter-Chen12-3   GTCTCAAGTCTAAAGGGGCCAACTAATATAAAACGGAGGGAGTATTTAAGAGAAAATAAT :  60
Promoter-S18        GTCTCAAGTCTAAAGGGGCCAACTAATATAAAACGGAGGGAGTATTTAAGAGAAAATAAT :  60
Promoter-ZL         GTCTCAAGTCTAAAGGGGCCAACTAATATAAAACGGAGGGAGTATTTAAGAGAAAATAAT :  60
Promoter-BZ         GTCTCAAGTCTAAAGGGGCCAACTAATATAAAACGGAGGGAGTATTTAAGAGAAAATAAT :  60
Promoter-THIP       GTCTCAAGTCTAAAGGGGCCAACTAATATAAAACGGAGGGAGTATTTAAGAGAAAATAAT :  60
Promoter-Chen12-4   GTCTCAAGTCTAAAGGGGCCAACTAATATAAAACGGAGGGAGTATTTAAGAGAAAATAAT :  60
Promoter-LJBN002    GTCTCAAGTCTAAAGGGGCCAACTAATATAAAACGGAGGGAGTATTTAAGAGAAAATAAT :  60
Consensus           GTCTCAAGTCTAAAGGGGCCAACTAATATAAAACGGAGGGAGTATTTAAGAGAAAATAAT

Promoter-Z101       ATGAGTAGAAGATACTTCCTCCGTTTCAAATTATTGGTCTCAAATTTTCTAATTTGATTT : 120
Promoter-M38        ATGAGTAGAAGATACTTCCTCCGTTTCAAATTATTGGTCTCAAATTTTCTAATTTGATTT : 120
Promoter-ZFJ        ATGAGTAGAAGATACTTCCTCCGTTTCAAATTATTGGTCTCAAATTTTCTAATTTGATTT : 120
Promoter-Chen12-3   ATGAGTAGAAGATACTTCCTCCGTTTCAAATTATTGGTCTCAAATTTTCTAATTTGATTT : 120
Promoter-S18        ATGAGTAGAAGATACTTCCTCCGTTTCAAATTATTGGTCTCAAATTTTCTAATTTGATTT : 120
Promoter-ZL         ATGAGTAGAAGATACTTCCTCCGTTTCAAATTATTGGTCTCAAATTTTCTAATTTGATTT : 120
Promoter-BZ         ATGAGTAGAAGATACTTCCTCCGTTTCAAATTATTGGTCTCAAATTTTCTAATTTGATTT : 120
Promoter-THIP       ATGAGTAGAAGATACTTCCTCCGTTTCAAATTATTGGTCTCAAATTTTCTAATTTGATTT : 120
Promoter-Chen12-4   ATGAGGAGAAGATACTTCCTCCGTTTCAAATTATTGGTCCCAAATTTTCTAATTTGATTT : 120
Promoter-LJBN002    ATGAGTAGAAGATACTTCCTCCGTTTCAAATTATTGGTCCCAAATTTTCTAATTTGATTT : 120
Consensus           ATGAG AGAAGATACTTCCTCCGTTTCAAATTATTGGTC CAAATTTTCTAATTTGATTT

Promoter-Z101       ATCATTTTACTTGTCTTTTTCCATTAATCAAGAAGAAATAATTTTTTTTTCCACGTTTTAT : 180
Promoter-M38        ATCATTTTACTTGTCTTTTTCCATTAATCAAGAAGAAATAATTTTTTTTTCCACGTTTTAT : 180
Promoter-ZFJ        ATCATTTTACTTGTCTTTTTCCATTAATCAAGAAGAAATAATTTTTTTTTCCACGTTTTAT : 180
Promoter-Chen12-3   ATCATTTTACTTGTCTTTTTCCATTAATCAAGAAGAAATAATTTTTTTTTCCACGTTTTAT : 180
Promoter-S18        ATCATTTTACTTGTCTTTTTCCATTAATCAAGAAGAAATAATTTTTTTTTCCACGTTTTAT : 180
Promoter-ZL         ATCATTTTACTTGTCTTTTTCCATTAATCAAGAAGAAATAATTTTTTTTTCCACGTTTTAT : 180
Promoter-BZ         ATCATTTTACTTGTCTTTTTCCATTAATCAAGAAGAAATAATTTTTTTTTCCACGTTTTAT : 180
Promoter-THIP       ATCATTTTACTTGTCTTTTTCCATTAATCAAGAAGAAATAATTTTTTTTTCCACGTTTTAT : 180
Promoter-Chen12-4   CTCATTTTACTTGTCTTTTTTCATTAATCAAGAAGAAACAATTTTTTTTTCCATGTTTTAT : 180
Promoter-LJBN002    CTCATTTTACTTGTCGTTTTTCATTAATCAAGAAGAAACAATTTTTTTTTCCATGTTTTAT : 180
Consensus            TCATTTTACTTGTC TTTT CATTAATCAAGAAGAAA AATTTTTTTTTCCA GTTTTAT

Promoter-Z101       CTTTTGCATTAGTTACTTTTTCTTTAAATTAAAATGTAAACATTATTTAATAGGGGTAGT : 240
Promoter-M38        CTTTTGCATTAGTTACTTTTTCTTTAAATTAAAATGTAAACATTATTTAATAGGGGTAGT : 240
Promoter-ZFJ        CTTTTGCATTAGTTACTTTTTCTTTAAATTAAAATGTAAACATTATTTAATAGGGGTAGT : 240
Promoter-Chen12-3   CTTTTGCATTAGTTACTTTTTCTTTAAATTAAAATGTAAACATTATTTAATAGGGGTAGT : 240
Promoter-S18        CTTTTGCATTAGTTACTTTTTCTTTAAATTAAAATGTAAACATTATTTAATAGGGGTAGT : 240
Promoter-ZL         CTTTTGCATTAGTTACTTTTTCTTTAAATTAAAATGTAAACATTATTTAATAGGGGTAGT : 240
Promoter-BZ         CTTTTGCATTAGTTACTTTTTCTTTAAATTAAAATGTAAACATTATTTAATAGGGGTAGT : 240
Promoter-THIP       CTTTTGCATTAGTTACTTTTTCTTTAAATTAAAATGTAAACATTATTTAATAGGGGTAGT : 240
Promoter-Chen12-4   CTTTTGCATTAGTTACTTTTTCTTTAAATTAAAATGTAAAAATTATTTAATAGGGGTAGT : 240
Promoter-LJBN002    CTTTTGCATTAGTTACTTTTTCTTTAAATTAAAATGTAAACATTATTTAATAGGAGTAGT : 240
Consensus           CTTTTGCATTAGTTACTTTTTCTTTAAATTAAAATGTAAA ATTATTTAATAGG GTAGT

Promoter-Z101       ATGGTAAATTAGCTATGTTATTCATTATTTTTCTTAATCAATGTGCCATCTTAATTTGAG : 300
Promoter-M38        ATGGTAAATTAGCTATGTTATTCATTATTTTTCTTAATCAATGTGCCATCTTAATTTGAG : 300
Promoter-ZFJ        ATGGTAAATTAGCTATGTTATTCATTATTTTTCTTAATCAATGTGCCATCTTAATTTGAG : 300
Promoter-Chen12-3   ATGGTAAATTAGCTATGTTATTCATTATTTTTCTTAATCAATGTGCCATCTTAATTTGAG : 300
Promoter-S18        ATGGTAAATTAGCTATGTTATTCATTATTTTTCTTAATCAATGTGCCATCTTAATTTGAG : 300
Promoter-ZL         ATGGTAAATTAGCTATGTTATTCATTATTTTTCTTAATCAATGTGCCATCTTAATTTGAG : 300
Promoter-BZ         ATGGTAAATTAGCTATGTTATTCATTATTTTTCTTAATCAATGTGCCATCTTAATTTGAG : 300
Promoter-THIP       ATGGTAAATTAGCTATGTTATTCATTATTTTTCTTAATCAATGTGCCATCTTAATTTGAG : 300
Promoter-Chen12-4   ATGGTAAATTAGCTATGTTATTTATTATTTTTCTTAATCAATGTGCCATCTTAATTTGGG : 300
Promoter-LJBN002    ATGGTAAATTAGCCATGTTATTTATTATTTTTCTTAATCAATGTGCCATCTTAATTTGGG : 300
Consensus
```

```
                      ATGGTAAATTAGC ATGTTATT ATTATTTTTCTTAATCAATGTGCCATCTTAATTTG G

Promoter-Z101         ACATGGGAGTAATACTCAACG----ATATATATATTTAAATGCATTAAAAGATTCGATAC : 356
Promoter-M38          ACATGGGAGTAATACTCAACG----ATATATATATTTAAATGCATTAAAAGATTCGATAC : 356
Promoter-ZFJ          ACATGGGAGTAATACTCAACG----ATATATATATTTAAATGCATTAAAAGATTCGATAC : 356
Promoter-Chen12-3     ACATGGGAGTAATACTCAACG----ATATATATATTTAAATGCATTAAAAGATTCGATAC : 356
Promoter-S18          ACATGGGAGTAATACTCAACG----ATATATATATTTAAATGCATTAAAAGATTCGATAC : 356
Promoter-ZL           ACATGGGAGTAATACTCAACG----ATATATATATTTAAATGCATTAAAAGATTCGATAC : 356
Promoter-BZ           ACATGGGAGTAATACTCAACG----ATATATATATTTAAATGCATTAAAAGATTCGATAC : 356
Promoter-THIP         ACATGGGAGTAATACTCAACG----ATATATATATTTAAATGCATTAAAAGATTCGATAC : 356
Promoter-Chen12-4     ACATGGGAGTAATACTCAACG----ATATATATATTTAAGTGCATTAAAAGATTCGATAC : 356
Promoter-LJBN002      ACATGAGAGTAATACTCAACGATATATATATATATTTAAGTGCATTAAAAGGTTCGATAC : 360
Consensus             ACATG GAGTAATACTCAACG     ATATATATATTTAA TGCATTAAAAG TTCGATAC

Promoter-Z101         TTTTTTCAAATGCCTATCATTTAGGGGCGGATCAGATTCATCTTTCATCTAGAGGATTCA : 416
Promoter-M38          TTTTTTCAAATGCCTATCATTTAGGGGCGGATCAGATTCATCTTTCATCTAGAGGATTCA : 416
Promoter-ZFJ          TTTTTTCAAATGCCTATCATTTAGGGGCGGATCAGATTCATCTTTCATCTAGAGGATTCA : 416
Promoter-Chen12-3     TTTTTTCAAATGCCTATCATTTAGGGGCGGATCAGATTCATCTTTCATCTAGAGGATTCA : 416
Promoter-S18          TTTTTTCAAATGCCTATCATTTAGGGGCGGATCAGATTCATCTTTCATCTAGAGGATTCA : 416
Promoter-ZL           TTTTTTCAAATGCCTATCATTTAGGGGCGGATCAGATTCATCTTTCATCTAGAGGATTCA : 416
Promoter-BZ           TTTTTTCAAATGCCTATCATTTAGGGGCGGATCAGATTCATCTTTCATCTAGAGGATTCA : 416
Promoter-THIP         TTTTTTCAAATGCCTATCATTTAGGGGCGGATCAGATTCATCTTTCATCTAGAGGATTCA : 416
Promoter-Chen12-4     TTTTTT-AAATGCCTATCATTTAGGGGTGGATCAGATTCATCTTTCATCTTGAGGATTCA : 415
Promoter-LJBN002      TTTTTTCAAATGCCTATCATTTAGGGGCGGATCAGATTCATCTTTCATCTAGAGGATTCA : 420
Consensus             TTTTTT AAATGCCTATCATTTAGGGG GGATCAGATTCATCTTTCATCT GAGGATTCA

Promoter-Z101         TCCGAATTCTTTTCGACGAAAAATAATACTATTTTTATATAATTGAAAATAATTTTTATG : 476
Promoter-M38          TCCGAATTCTTTTCGACGAAAAATAATACTATTTTTATATAATTGAAAATAATTTTTATG : 476
Promoter-ZFJ          TCCGAATTCTTTTCGACGAAAAATAATACTATTTTTATATAATTGAAAATAATTTTTATG : 476
Promoter-Chen12-3     TCCGAATTCTTTTCGACGAAAAATAATACTATTTTTATATAATTGAAAATAATTTTTATG : 476
Promoter-S18          TCCGAATTCTTTTCGACGAAAAATAATACTATTTTTATATAATTGAAAATAATTTTTATG : 476
Promoter-ZL           TCCGAATTCTTTTCGACGAAAAATAATACTATTTTTATATAATTGAAAATAATTTTTATG : 476
Promoter-BZ           TCCGAATTCTTTTCGACGAAAAATAATACTATTTTTATATAATTGAAAATAATTTTTATG : 476
Promoter-THIP         TCCGAATTCTTTTCGACGAAAAATAATACTATTTTTATATAATTGAAAATAATTTTTATG : 476
Promoter-Chen12-4     TCCGAATCTTTTTCGACGAAAAATAATACTATTTTTATATAATTGCAAATAATTTTTATG : 475
Promoter-LJBN002      TCCGAATCCTTTTCAACGAAAAATGATACTATTTTTATATAATTGAAAATAATTTTTATG : 480
Consensus             TCCGAAT  TTTTC ACGAAAAAT ATACTATTTTTATATAATTG AAATAATTTTTATG

Promoter-Z101         TATAGTTCGTATGTTTACTTCTGAATCTCCTTAATGAAAATTCTGCCTCGACCACTATAA : 536
Promoter-M38          TATAGTTCGTATGTTTACTTCTGAATCTCCTTAATGAAAATTCTGCCTCGACCACTATAA : 536
Promoter-ZFJ          TATAGTTCGTATGTTTACTTCTGAATCTCCTTAATGAAAATTCTGCCTCGACCACTATAA : 536
Promoter-Chen12-3     TATAGTTCGTATGTTTACTTCTGAATCTCCTTAATGAAAATTCTGCCTCGACCACTATAA : 536
Promoter-S18          TATAGTTCGTATGTTTACTTCTGAATCTCCTTAATGAAAATTCTGCCTCGACCACTATAA : 536
Promoter-ZL           TATAGTTCGTATGTTTACTTCTGAATCTCCTTAATGAAAATTCTGCCTCGACCACTATAA : 536
Promoter-BZ           TATAGTTCGTATGTTTACTTCTGAATCTCCTTAATGAAAATTCTGCCTCGACCACTATAA : 536
Promoter-THIP         TATAGTTCGTATGTTTACTTCTGAATCTCCTTAATGAAAATTCTGCCTCGACCACTATAA : 536
Promoter-Chen12-4     TATAGTTCGTATGTTTACTTTTGAATCTCCTTAATGAAAATTCTGCCTCAGCCACTGTAA : 535
Promoter-LJBN002      TATAGTTCGTATGTTTACTTCTGAATCTCCTTAATGAAAATTCTGCCTCGGCCACTATAA : 540
Consensus             TATAGTTCGTATGTTTACTT TGAATCTCCTTAATGAAAATTCTGCCTC  CCACT TAA

Promoter-Z101         AATTTTGTATCGTCTGAAAGAAATTTTTCAAAACTAAAATTAGACAAAATCTTACCTAAG : 596
Promoter-M38          AATTTTGTATCGTCTGAAAGAAATTTTTCAAAACTAAAATTAGACAAAATCTTACCTAAG : 596
Promoter-ZFJ          AATTTTGTATCGTCTGAAAGAAATTTTTCAAAACTAAAATTAGACAAAATCTTACCTAAG : 596
Promoter-Chen12-3     AATTTTGTATCGTCTGAAAGAAATTTTTCAAAACTAAAATTAGACAAAATCTTACCTAAG : 596
Promoter-S18          AATTTTGTATCGTCTGAAAGAAATTTTTCAAAACTAAAATTAGACAAAATCTTACCTAAG : 596
Promoter-ZL           AATTTTGTATCGTCTGAAAGAAATTTTTCAAAACTAAAATTAGACAAAATCTTACCTAAG : 596
Promoter-BZ           AATTTTGTATCGTCTGAAAGAAATTTTTCAAAACTAAAATTAGACAAAATCTTACCTAAG : 596
Promoter-THIP         AATTTTGTATCGTCTGAAAGAAATTTTTCAAAACTAAAATTAGACAAAATCTTACCTAAG : 596
Promoter-Chen12-4     AATTTTGTATCGTTTGAAAGAAATTTTTCAAAACTAAAATTAGACAAAATCTTACCTAAG : 595
Promoter-LJBN002      AATTTTGTATCATTTGAAAGAAATTTTTCAAAACTAAAATTAGACAAAATCTTACGTAAG : 600
Consensus             AATTTTGTATC T TGAAAGAAATTTTTCAAAACTAAAATTAGACAAAATCTTAC TAAG

Promoter-Z101         ATCAATAACCCCTCACAAGTGATTTAATCGGTTAACTACATTACCAATTGTTGAATAATA : 656
Promoter-M38          ATCAATAACCCCTCACAAGTGATTTAATCGGTTAACTACATTACCAATTGTTGAATAATA : 656
Promoter-ZFJ          ATCAATAACCCCTCACAAGTGATTTAATCGGTTAACTACATTACCAATTGTTGAATAATA : 656
```

```
Promoter-Chen12-3   ATCAATAACCCCTCACAAGTGATTTAATCGGTTAACTACATTACCAATTGTTGAATAATA :  656
Promoter-S18        ATCAATAACCCCTCACAAGTGATTTAATCGGTTAACTACATTACCAATTGTTGAATAATA :  656
Promoter-ZL         ATCAATAACCCCTCACAAGTGATTTAATCGGTTAACTACATTACCAATTGTTGAATAATA :  656
Promoter-BZ         ATCAATAACCCCTCACAAGTGATTTAATCGGTTAACTACATTACCAATTGTTGAATAATA :  656
Promoter-THIP       ATCAATAACCCCTCACAAGTGATTTAATCGGTTAACTACATTACCAATTGTTGAATAATA :  656
Promoter-Chen12-4   ATCAATAACCCCTCACAAGTGATTTAATCGGTTAACTACATTACCAATTGTTAAATAATA :  655
Promoter-LJBN002    ATCAATAACCCCTCACAAGTGATTTAATCGGTTAACTACATTACCAATTGTTGAATAATA :  660
Consensus           ATCAATAACCCCTCACAAGTGATTTAATCGGTTAACTACATTACCAATTGTT AATAATA

Promoter-Z101       ATCAATAAAAATACTGTAAAATTATTAT-------TTTCATTTTAAAAAATTGAAAATAT :  709
Promoter-M38        ATCAATAAAAATACTGTAAAATTATTAT-------TTTCATTTTAAAAAATTGAAAATAT :  709
Promoter-ZFJ        ATCAATAAAAATACTGTAAAATTATTAT-------TTTCATTTTAAAAAATTGAAAATAT :  709
Promoter-Chen12-3   ATCAATAAAAATACTGTAAAATTATTAT-------TTTCATTTTAAAAAATTGAAAATAT :  709
Promoter-S18        ATCAATAAAAATACTGTAAAATTATTAT-------TTTCATTTTAAAAAATTGAAAATAT :  709
Promoter-ZL         ATCAATAAAAATACTGTAAAATTATTAT-------TTTCATTTTAAAAAATTGAAAATAT :  709
Promoter-BZ         ATCAATAAAAATACTGTAAAATTATTAT-------TTTCATTTTAAAAAATTGAAAATAT :  709
Promoter-THIP       ATCAATAAAAATACTGTAAAATTATTAT-------TTTCATTTTAAAAAATTGAAAATAT :  709
Promoter-Chen12-4   ATCAATAAAAATACTGTAAAATTATTATAGTATTTTTTCTTTTTAAAATATTGAAAATAT :  715
Promoter-LJBN002    ATCAATAAAAATACTTTAAAATTATTATAGTATTTTTTCATTTTAAAAAATTGAAAATAT :  720
Consensus           ATCAATAAAAATACT TAAAATTATTAT       TTTC TTTTAAAA ATTGAAAATAT

Promoter-Z101       ATTCCAAATGCATATAGATATATGTGTGAATATTTTTAAAAGAAGCTTAGACCTCTAATT :  769
Promoter-M38        ATTCCAAATGCATATAGATATATGTGTGAATATTTTTAAAAGAAGCTTAGACCTCTAATT :  769
Promoter-ZFJ        ATTCCAAATGCATATAGATATATGTGTGAATATTTTTAAAAGAAGCTTAGACCTCTAATT :  769
Promoter-Chen12-3   ATTCCAAATGCATATAGATATATGTGTGAATATTTTTAAAAGAAGCTTAGACCTCTAATT :  769
Promoter-S18        ATTCCAAATGCATATAGATATATGTGTGAATATTTTTAAAAGAAGCTTAGACCTCTAATT :  769
Promoter-ZL         ATTCCAAATGCATATAGATATATGTGTGAATATTTTTAAAAGAAGCTTAGACCTCTAATT :  769
Promoter-BZ         ATTCCAAATGCATATAGATATATGTGTGAATATTTTTAAAAGAAGCTTAGACCTCTAATT :  769
Promoter-THIP       ATTCCAAATGCATATAGATATATGTGTGAATATTTTTAAAAGAAGCTTAGACCTCTAATT :  769
Promoter-Chen12-4   ATTCCAAATGCATATAGATATATGTGTGAATATTTTTAAAAGAAGCTTAGACCTCTAATT :  775
Promoter-LJBN002    ATTCCAAATGCATATAGATATATGTGTGAATATTTTTAAAAGAAGCTTAGACCTCTAATT :  780
Consensus           ATTCCAAATGCATATAGATATATGTGTGAATATTTTTAAAAGAAGCTTAGACCTCTAATT

Promoter-Z101       ATAGTTTCGCCTTATGCCTCCAATTATGTTAAGCTGAGCTAGTTAGAGGAACACAGTCAC :  829
Promoter-M38        ATAGTTTCGCCTTATGCCTCCAATTATGTTAAGCTGAGCTAGTTAGAGGAACACAGTCAC :  829
Promoter-ZFJ        ATAGTTTCGCCTTATGCCTCCAATTATGTTAAGCTGAGCTAGTTAGAGGAACACAGTCAC :  829
Promoter-Chen12-3   ATAGTTTCGCCTTATGCCTCCAATTATGTTAAGCTGAGCTAGTTAGAGGAACACAGTCAC :  829
Promoter-S18        ATAGTTTCGCCTTATGCCTCCAATTATGTTAAGCTGAGCTAGTTAGAGGAACACAGTCAC :  829
Promoter-ZL         ATAGTTTCGCCTTATGCCTCCAATTATGTTAAGCTGAGCTAGTTAGAGGAACACAGTCAC :  829
Promoter-BZ         ATAGTTTCGCCTTATGCCTCCAATTATGTTAAGCTGAGCTAGTTAGAGGAACACAGTCAC :  829
Promoter-THIP       ATAGTTTCGCCTTATGCCTCCAATTATGTTAAGCTGAGCTAGTTAGAGGAACACAGTCAC :  829
Promoter-Chen12-4   ATAGTTTCGCCTTATGCATCCAATTATGTTAAGCTGAGCTAGTTAGAGGAACACAGTCAC :  835
Promoter-LJBN002    ATAGTTTTGCCTTATGCCTCCAATTATGTTAAGCTGAGCTAGTTAGGGGAACACAGTCAC :  840
Consensus           ATAGTTT GCCTTATGC TCCAATTATGTTAAGCTGAGCTAGTTAG GGAACACAGTCAC
Promoter-Z101       TGAAATAAGAAATTTTGTTAAGAATG---------------------------------- :  855
Promoter-M38        TGAAATAAGAAATTTTGTTAAGAATG---------------------------------- :  855
Promoter-ZFJ        TGAAATAAGAAATTTTGTTAAGAATG---------------------------------- :  856
Promoter-Chen12-3   TGAAATAAGAAATTTTGTTAAGAATG---------------------------------- :  856
Promoter-S18        TGAAATAAGAAATTTTGTTAAGAATG---------------------------------- :  855
Promoter-ZL         TGAAATAAGAAATTTTGTTAAGAATG---------------------------------- :  855
Promoter-BZ         TGAAATAAGAAATTTTGTTAAGAATG---------------------------------- :  855
Promoter-THIP       TGAAATAAGAAATTTTGTTAAGAATG---------------------------------- :  855
Promoter-Chen12-4   TGAAATAAGAAATTTTGTTAAGAATGTTGAAGATTTAATATATACTTATGAAAAGTATGT :  895
Promoter-LJBN002    TGAAATAAGAAATTTTGTTAAGAATGTTCAAGATTTAATATATACTTATGAAAAGTATGT :  900
Consensus           TGAAATAAGAAATTTTGTTAAGAATG

Promoter-Z101       -TTCATCAAT-GTTTTTCGGTACATGAATTCATATTTAGGCACAATAACTTCAACACTAG :  913
Promoter-M38        -TTCATCAAT-GTTTTTCGGTACATGAATTCATATTTAGGCACAATAACTTCAACACTAG :  913
Promoter-ZFJ        GTTCATCAATAGTTTTTCGGTACATGAATTCATATTTAGGCACAATAACTTCAACACTAG :  915
Promoter-Chen12-3   GTTCATCAATAGTTTTTCGGTACATGAATTCATATTTAGGCACAATAACTTCAACACTAG :  915
Promoter-S18        -TTCATCAAT-GTTTTTCGGTACATGAATTCATATTTAGGCACAATAACTTCAACACTAG :  913
Promoter-ZL         -TTCATCAAT-GTTTTTCGGTACATGAATTCATATTTAGGCACAATAACTTCAACACTAG :  913
Promoter-BZ         -TTCATCAAT-GTTTTTCGGTACATGAATTCATATTTAGGCACAATAACTTCAACACTAG :  913
Promoter-THIP       -TTCATCAAT-GTTTTTCGGTACATGAATTCATATTTAGGCACAATAACTTCAACACTAG :  913
```

```
Promoter-Chen12-4   TGTTTTCAAT-GTTTTTCGGTACATGAATTCATATTTAGTGACAATAACTTCAAGACTAG :  954
Promoter-LJBN002    TGTTTTCAAT-GTTTTTCGGTACATGAATTCATATTTAGGCACAATAACTTCAATATTAG :  959
Consensus             T  GTCAAT GTTTTTCGGTACATGAATTCATATTTAG  ACAATAACTTCAA A TAG
Promoter-Z101       TTCAAGTTCAAAACAAGAACACTATGAAGTACAAAAACACCTTCAACACAAGATCAAAGT :  973
Promoter-M38        TTCAAGTTCAAAACAAGAACACTATGAAGTACAAAAACACCTTCAACACAAGATCAAAGT :  973
Promoter-ZFJ        TTCAAGTTCAAAACAAGAACACTATGAAGTACAAAAACACCTTCAACACAAGATCAAAGT :  975
Promoter-Chen12-3   TTCAAGTTCAAAACAAGAACACTATGAAGTACAAAAACACCTTCAACACAAGATCAAAGT :  975
Promoter-S18        TTCAAGTTCAAAACAAGAACACTATGAAGTACAAAAACACCTTCAACACAAGATCAAAGT :  973
Promoter-ZL         TTCAAGTTCAAAACAAGAACACTATGAAGTACAAAAACACCTTCAACACAAGATCAAAGT :  973
Promoter-BZ         TTCAAGTTCAAAACAAGAACACTATGAAGTACAAAAACACCTTCAACACAAGATCAAAGT :  973
Promoter-THIP       TTCAAGTTCAAAACAAGAACACTATGAAGTACAAAAACACCTTCAACACAAGATCAAAGT :  973
Promoter-Chen12-4   TTCAAATTCAAAACAAGAACACTATGAAGTACAAAAACACCTTCACCACAAGATCAAAGT : 1014
Promoter-LJBN002    TTCAAGTTTAAAACAAGAACACTATGAAGTATAAAAACACCTTCAACACAAGATTAAAAT : 1019
Consensus           TTCAA TT AAAACAAGAACACTATGAAGTA AAAAACACCTTCA CACAAGAT AAA T

Promoter-Z101       GATCTTTCTAAGAAGTGTGTTTTATTTTTTTTTAGAAATTAAGATGAAACAGAAATATAA : 1033
Promoter-M38        GATCTTTCTAAGAAGTGTGTTTTATTTTTTTTTAGAAATTAAGATGAAACAGAAATATAA : 1033
Promoter-ZFJ        GATCTTTCTAAGAAGTGTGTTTTATTTTTTTTTAGAAATTAAGATGAAACAGAAATATAA : 1035
Promoter-Chen12-3   GATCTTTCTAAGAAGTGTGTTTTATTTTTTTTTAGAAATTAAGATGAAACAGAAATATAA : 1035
Promoter-S18        GATCTTTCTAAGAAGTGTGTTTTATTTTTTTTTAGAAATTAAGATGAAACAGAAATATAA : 1033
Promoter-ZL         GATCTTTCTAAGAAGTGTGTTTTATTTTTTTTTAGAAATTAAGATGAAACAGAAATATAA : 1033
Promoter-BZ         GATCTTTCTAAGAAGTGTGTTTTATTTTTTTTTAGAAATTAAGATGAAACAGAAATATAA : 1033
Promoter-THIP       GATCTTTCTAAGAAGTGTGTTTTATTTTTTTTTAGAAATTAAGATGAAACAGAAATATAA : 1033
Promoter-Chen12-4   GATCTTTCTAAGAAGTGTGTTTTATTTTTTTT-AGAAATTAAGATGAAACAAAAATATAA : 1073
Promoter-LJBN002    GATCTTTCTAAGAAGTGTGTTTTATTTTTTTT-AGAAATTAAGATGAAATAGAAATATAA : 1078
Consensus           GATCTTTCTAAGAAGTGTGTTTTATTTTTTTT AGAAATTAAGATGAAA A AAATATAA

Promoter-Z101       AGTCAAGTCCTCCGACTTCACGGAGTGTCCTTAAAGAATAATTCCCCTCACTGTACCTGA : 1093
Promoter-M38        AGTCAAGTCCTCCGACTTCACGGAGTGTCCTTAAAGAATAATTCCCCTCACTGTACCTGA : 1093
Promoter-ZFJ        AGTCAAGTCCTCCGACTTCACGGAGTGTCCTTAAAGAATAATTCCCCTCACTGTACCTGA : 1095
Promoter-Chen12-3   AGTCAAGTCCTCCGACTTCACGGAGTGTCCTTAAAGAATAATTCCCCTCACTGTACCTGA : 1095
Promoter-S18        AGTCAAGTCCTCCGACTTCACGGAGTGTCCTTAAAGAATAATTCCCCTCACTGTACCTGA : 1093
Promoter-ZL         AGTCAAGTCCTCCGACTTCACGGAGTGTCCTTAAAGAATAATTCCCCTCACTGTACCTGA : 1093
Promoter-BZ         AGTCAAGTCCTCCGACTTCACGGAGTGTCCTTAAAGAATAATTCCCCTCACTGTACCTGA : 1093
Promoter-THIP       AGTCAAGTCCTCCGACTTCACGGAGTGTCCTTAAAGAATAATTCCCCTCACTGTACCTGA : 1093
Promoter-Chen12-4   AGTCAAGTCCTCCGACTTCACGGAGTGTCCTTAAAGAATAATTTCCCTCACTGTACCCGA : 1133
Promoter-LJBN002    AGTCAAGTCCTCCGACTTCACGGAGTGTCCTTAAAGAATAATTCCCCTCACTGTACCCGA : 1138
Consensus           AGTCAAGTCCTCCGACTTCACGGAGTGTCCTTAAAGAATAATT CCCTCACTGTACC GA
Promoter-Z101       GGTTTTAGAATCTTCCTCCCAGGATAAAATGACTTTCAATTCAACTATAGTGGTACCTCA : 1153
Promoter-M38        GGTTTTAGAATCTTCCTCCCAGGATAAAATGACTTTCAATTCAACTATAGTGGTACCTCA : 1153
Promoter-ZFJ        GGTTTTAGAATCTTCCTCCCAGGATAAAATGACTTTCAATTCAACTATAGTGGTACCTCA : 1155
Promoter-Chen12-3   GGTTTTAGAATCTTCCTCCCAGGATAAAATGACTTTCAATTCAACTATAGTGGTACCTCA : 1155
Promoter-S18        GGTTTTAGAATCTTCCTCCCAGGATAAAATGACTTTCAATTCAACTATAGTGGTACCTCA : 1153
Promoter-ZL         GGTTTTAGAATCTTCCTCCCAGGATAAAATGACTTTCAATTCAACTATAGTGGTACCTCA : 1153
Promoter-BZ         GGTTTTAGAATCTTCCTCCCAGGATAAAATGACTTTCAATTCAACTATAGTGGTACCTCA : 1153
Promoter-THIP       GGTTTTAGAATCTTCCTCCCAGGATAAAATGACTTTCAATTCAACTATAGTGGTACCTCA : 1153
Promoter-Chen12-4   GGTTTTGGAATCTTTCTCCCAGGATAAAATGACTTTCAATTCAACTATAGTGGTACCTCA : 1193
Promoter-LJBN002    GGTTTTGGAATCTTCCTTCTAGGATAAAATGACTTTCAATTCAACTATAGTGGTACCTCA : 1198
Consensus           GGTTTT GAATCTT CT C AGGATAAAATGACTTTCAATTCAACTATAGTGGTACCTCA
Promoter-Z101       AATAATTGGATTCGTCGAACTCACTCAATGATTTGATTGATCACAAGGAATGTTTTGTAG : 1213
Promoter-M38        AATAATTGGATTCGTCGAACTCACTCAATGATTTGATTGATCACAAGGAATGTTTTGTAG : 1213
Promoter-ZFJ        AATAATTGGATTCGTCGAACTCACTCAATGATTTGATTGATCACAAGGAATGTTTTGTAG : 1215
Promoter-Chen12-3   AATAATTGGATTCGTCGAACTCACTCAATGATTTGATTGATCACAAGGAATGTTTTGTAG : 1215
Promoter-S18        AATAATTGGATTCGTCGAACTCACTCAATGATTTGATTGATCACAAGGAATGTTTTGTAG : 1213
Promoter-ZL         AATAATTGGATTCGTCGAACTCACTCAATGATTTGATTGATCACAAGGAATGTTTTGTAG : 1213
Promoter-BZ         AATAATTGGATTCGTCGAACTCACTCAATGATTTGATTGATCACAAGGAATGTTTTGTAG : 1213
Promoter-THIP       AATAATTGGATTCGTCGAACTCACTCAATGATTTGATTGATCACAAGGAATGTTTTGTAG : 1213
Promoter-Chen12-4   AATAATTGGATTCGTCGAACTCACTCAATGATTTGATTGATCACAAGGAATGTTTTGTAG : 1253
Promoter-LJBN002    AATAATTGAATTCGTCGAACTCACTCAATGATTTGATTGATCACAAGGAATGTTTTGTAG : 1258
Consensus           AATAATTG ATTCGTCGAACTCACTCAATGATTTGATTGATCACAAGGAATGTTTTGTAG

Promoter-Z101       ACAAGAAGAGTTTTTATTTTAGAAAATTTTTCATTCCAAAACCTGTGGATAAATGCAGGT : 1273
Promoter-M38        ACAAGAAGAGTTTTTATTTTAGAAAATTTTTCATTCCAAAACCTGTGGATAAATGCAGGT : 1273
Promoter-ZFJ        ACAAGAAGAGTTTTTATTTTAGAAAATTTTTCATTCCAAAACCTGTGGATAAATGCAGGT : 1275
Promoter-Chen12-3   ACAAGAAGAGTTTTTATTTTAGAAAATTTTTCATTCCAAAACCTGTGGATAAATGCAGGT : 1275
```

```
Promoter-S18          ACAAGAAGAGTTTTTATTTTAGAAAATTTTTCATTCCAAAACCTGTGGATAAATGCAGGT : 1273
Promoter-ZL           ACAAGAAGAGTTTTTATTTTAGAAAATTTTTCATTCCAAAACCTGTGGATAAATGCAGGT : 1273
Promoter-BZ           ACAAGAAGAGTTTTTATTTTAGAAAATTTTTCATTCCAAAACCTGTGGATAAATGCAGGT : 1273
Promoter-THIP         ACAAGAAGAGTTTTTATTTTAGAAAATTTTTCATTCCAAAACCTGTGGATAAATGCAGGT : 1273
Promoter-Chen12-4     ACAAGAAGAGTTTTTATTTTAGAAAATTTTTCATTTCAAAACCTGTGGATAAATGCAGGT : 1313
Promoter-LJBN002      ACAAGAAGAGTTTTTATTTTAGAAAATTTTTCATTCCAAAACCTGTGGATAAATGCAGGT : 1318
Consensus             ACAAGAAGAGTTTTTATTTTAGAAAATTTTTCATT CAAAACCTGTGGATAAATGCAGGT

Promoter-Z101         TTATATAGCCATCAAGTACCTCTTCCGAAAGGTGGCA-TGGTTCATCCGAAAAGGTGTAT : 1332
Promoter-M38          TTATATAGCCATCAAGTACCTCTTCCGAAAGGTGGCA-TGGTTCATCCGAAAAGGTGTAT : 1332
Promoter-ZFJ          TTATATAGCCATCAAGTACCTCTTCCGAAAGGTGGCA-TGGTTCATCCGAAAAGGTGTAT : 1334
Promoter-Chen12-3     TTATATAGCCATCAAGTACCTCTTCCGAAAGGTGGCA-TGGTTCATCCGAAAAGGTGTAT : 1334
Promoter-S18          TTATATAGCCATCAAGTACCTCTTCCGAAAGGTGGCA-TGGTTCATCCGAAAAGGTGTAT : 1332
Promoter-ZL           TTATATAGCCATCAAGTACCTCTTCCGAAAGGTGGCA-TGGTTCATCCGAAAAGGTGTAT : 1332
Promoter-BZ           TTATATAGCCATCAAGTACCTCTTCCGAAAGGTGGCA-TGGTTCATCCGAAAAGGTGTAT : 1332
Promoter-THIP         TTATATAGCCATCAAGTACCTCTTCCGAAAGGTGGCA-TGGTTCATCCGAAAAGGTGTAT : 1332
Promoter-Chen12-4     TTATATAGCCATCAAGTACCTCTTCCGAAAGGTGGCAATGGTTCATCCGAAAAGGTGTAT : 1373
Promoter-LJBN002      TTATATTGCCATCAAGTACCTCTTCCGAAAGGTGGCAATGGTTCATCCGAAAAGGTGTAT : 1378
Consensus             TTATAT GCCATCAAGTACCTCTTCCGAAAGGTGGCA TGGTTCATCCGAAAAGGTGTAT

Promoter-Z101         CCTTTGGAAGAGTCATATCTATTCATGCGAAACATTGTGTCTTTTTCTGAGCAGATACAA : 1392
Promoter-M38          CCTTTGGAAGAGTCATATCTATTCATGCGAAACATTGTGTCTTTTTCTGAGCAGATACAA : 1392
Promoter-ZFJ          CCTTTGGAAGAGTCATATCTATTCATGCGAAACATTGTGTCTTTTTCTGAGCAGATACAA : 1394
Promoter-Chen12-3     CCTTTGGAAGAGTCATATCTATTCATGCGAAACATTGTGTCTTTTTCTGAGCAGATACAA : 1394
Promoter-S18          CCTTTGGAAGAGTCATATCTATTCATGCGAAACATTGTGTCTTTTTCTGAGCAGATACAA : 1392
Promoter-ZL           CCTTTGGAAGAGTCATATCTATTCATGCGAAACATTGTGTCTTTTTCTGAGCAGATACAA : 1392
Promoter-BZ           CCTTTGGAAGAGTCATATCTATTCATGCGAAACATTGTGTCTTTTTCTGAGCAGATACAA : 1392
Promoter-THIP         CCTTTGGAAGAGTCATATCTATTCATGCGAAACATTGTGTCTTTTTCTGAGCAGATACAA : 1392
Promoter-Chen12-4     CCTTTGGAAGAGTCATATCTATTCATTCGAAACATTGTGTTTTTTTCTGAGCAGATACAA : 1433
Promoter-LJBN002      CCTTTGGAAGAGTCATGTCTATTCATTCGAAACATTGTGTCTTTTTCTGAGCAGATACAA : 1438
Consensus             CCTTTGGAAGAGTCAT TCTATTCAT CGAAACATTGTGT TTTTTCTGAGCAGATACAA

Promoter-Z101         CTATCTGAACAG--------------------TCACACTATTTTCAAAATAATATGTCTT : 1432
Promoter-M38          CTATCTGAACAG--------------------TCACACTATTTTCAAAATAATATGTCTT : 1432
Promoter-ZFJ          CTATCTGAACAG--------------------TCACACTATTTTCAAAATAATATGTCTT : 1434
Promoter-Chen12-3     CTATCTGAACAG--------------------TCACACTATTTTCAAAATAATATGTCTT : 1434
Promoter-S18          CTATCTGAACAG--------------------TCACACTATTTTCAAAATAATATGTCTT : 1432
Promoter-ZL           CTATCTGAACAG--------------------TCACACTATTTTCAAAATAATATGTCTT : 1432
Promoter-BZ           CTATCTGAACAG--------------------TCACACTATTTTCAAAATAATATGTCTT : 1432
Promoter-THIP         CTATCTGAACAG--------------------TCACACTATTTTCAAAATAATATGTCTT : 1432
Promoter-Chen12-4     CTATCTGAACAGTCACACTATTTCCTGAACAGTCACACTATTTCCAAAACAATATGTCTT : 1493
Promoter-LJBN002      CTATCTGAACAG--------------------TCACACTATTTCCAAAACAATATGTCTT : 1478
Consensus             CTATCTGAACAG                        TCACACTATTT CAAAA AATATGTCTT

Promoter-Z101         TTCCTCAAAGGTTATGTCCCTCAATTTTTCATTCACACCAATTGAACCCAACAAAGTAAT : 1492
Promoter-M38          TTCCTCAAAGGTTATGTCCCTCAATTTTTCATTCACACCAATTGAACCCAACAAAGTAAT : 1492
Promoter-ZFJ          TTCCTCAAAGGTTATGTCCCTCAATTTTTCATTCACACCAATTGAACCCAACAAAGTAAT : 1494
Promoter-Chen12-3     TTCCTCAAAGGTTATGTCCCTCAATTTTTCATTCACACCAATTGAACCCAACAAAGTAAT : 1494
Promoter-S18          TTCCTCAAAGGTTATGTCCCTCAATTTTTCATTCACACCAATTGAACCCAACAAAGTAAT : 1492
Promoter-ZL           TTCCTCAAAGGTTATGTCCCTCAATTTTTCATTCACACCAATTGAACCCAACAAAGTAAT : 1492
Promoter-BZ           TTCCTCAAAGGTTATGTCCCTCAATTTTTCATTCACACCAATTGAACCCAACAAAGTAAT : 1492
Promoter-THIP         TTCCTCAAAGGTTATGTCCCTCAATTTTTCATTCACACCAATTGAACCCAACAAAGTAAT : 1492
Promoter-Chen12-4     TTCCTCAAAGATTATGTCCCTCAATTTTTCATTCACACCAATTGAACCCAACAAAGTAAT : 1553
Promoter-LJBN002      TTCCTCAAAGGTTATGTCCCTCAATTTTTCATTCACACCAATTGAACCCAACAAAGTAAT : 1538
Consensus             TTCCTCAAAG TTATGTCCCTCAATTTTTCATTCACACCAATTGAACCCAACAAAGTAAT

Promoter-Z101         CTTTGACCTATATACTTCGTACAATTTTCTATATTCACAATATAAATCTTTAATGAAGGA : 1552
Promoter-M38          CTTTGACCTATATACTTCGTACAATTTTCTATATTCACAATATAAATCTTTAATGAAGGA : 1552
Promoter-ZFJ          CTTTGACCTATATACTTCGTACAATTTTCTATATTCACAATATAAATCTTTAATGAAGGA : 1554
Promoter-Chen12-3     CTTTGACCTATATACTTCGTACAATTTTCTATATTCACAATATAAATCTTTAATGAAGGA : 1554
Promoter-S18          CTTTGACCTATATACTTCGTACAATTTTCTATATTCACAATATAAATCTTTAATGAAGGA : 1552
Promoter-ZL           CTTTGACCTATATACTTCGTACAATTTTCTATATTCACAATATAAATCTTTAATGAAGGA : 1552
Promoter-BZ           CTTTGACCTATATACTTCGTACAATTTTCTATATTCACAATATAAATCTTTAATGAAGGA : 1552
Promoter-THIP         CTTTGACCTATATACTTCGTACAATTTTCTATATTCACAATATAAATCTTTAATGAAGGA : 1552
```

```
Promoter-Chen12-4    CTTTGACCTATATACTTCGTACAATTTTCTATATACACAATATAAATCTTTAATGAAGGA : 1613
Promoter-LJBN002     CTTTGACCTATATACTTCGTGCAATTTTCTATATACACAATATAAATCTTTAATGAAGGA : 1596
Consensus            CTTTGACCTATATACTTCGT CAATTTTCTATAT CACAATATAAATCTTTAATGAAGGA

Promoter-Z101        TATTCAGTCGACCATACCCTTCACTGCGTACGGCTATGCCATTGCCAGAAGGGACACACG : 1612
Promoter-M38         TATTCAGTCGACCATACCCTTCACTGCGTACGGCTATGCCATTGCCAGAAGGGACACACG : 1612
Promoter-ZFJ         TATTCAGTCGACCATACCCTTCACTGCGTACGGCTATGCCATTGCCAGAAGGGACACACG : 1614
Promoter-Chen12-3    TATTCAGTCGACCATACCCTTCACTGCGTACGGCTATGCCATTGCCAGAAGGGACACACG : 1614
Promoter-S18         TATTCAGTCGACCATACCCTTCACTGCGTACGGCTATGCCATTGCCAGAAGGGACACACG : 1612
Promoter-ZL          TATTCAGTCGACCATACCCTTCACTGCGTACGGCTATGCCATTGCCAGAAGGGACACACG : 1612
Promoter-BZ          TATTCAGTCGACCATACCCTTCACTGCGTACGGCTATGCCATTGCCAGAAGGGACACACG : 1612
Promoter-THIP        TATTCAGTCGACCATACCCTTCACTGCGTACGGCTATGCCATTGCCAGAAGGGACACACG : 1612
Promoter-Chen12-4    TATTCAGTCGACCATACCCTTCACTGCGTACGGCTATGCCATTGCCAGAAGGGGCACATG : 1673
Promoter-LJBN002     T---------------CCCTTCACTGCGTACGGCTATGCCATTGCCAGAAGGGGCACATG : 1643
Consensus            T                CCCTTCACTGCGTACGGCTATGCCATTGCCAGAAGGG CACA G

Promoter-Z101        GGGAAGTTGGATCTTTTGGTTGAAAATGAGGGTTTACCGGCCGGCCTCGTGAGAGTGTGT : 1672
Promoter-M38         GGGAAGTTGGATCTTTTGGTTGAAAATGAGGGTTTACCGGCCGGCCTCGTGAGAGTGTGT : 1672
Promoter-ZFJ         GGGAAGTTGGATCTTTTGGTTGAAAATGAGGGTTTACCGGCCGGCCTCGTGAGAGTGTGT : 1674
Promoter-Chen12-3    GGGAAGTTGGATCTTTTGGTTGAAAATGAGGGTTTACCGGCCGGCCTCGTGAGAGTGTGT : 1674
Promoter-S18         GGGAAGTTGGATCTTTTGGTTGAAAATGAGGGTTTACCGGCCGGCCTCGTGAGAGTGTGT : 1672
Promoter-ZL          GGGAAGTTGGATCTTTTGGTTGAAAATGAGGGTTTACCGGCCGGCCTCGTGAGAGTGTGT : 1672
Promoter-BZ          GGGAAGTTGGATCTTTTGGTTGAAAATGAGGGTTTACCGGCCGGCCTCGTGAGAGTGTGT : 1672
Promoter-THIP        GGGAAGTTGGATCTTTTGGTTGAAAATGAGGGTTTACCGGCCGGCCTCGTGAGAGTGTGT : 1672
Promoter-Chen12-4    GGGAGGTTGGATCTTTTGGTTGAAAATGAGGGTTTACCGGCCGGCCTCGCGAGACTCTGT : 1733
Promoter-LJBN002     GGGAAGTTGGATCTTTTGGTTGAAAATGAGGGTTTACCGGCCGGCCTGGTGAGAGTGTGT : 1703
Consensus            GGGA GTTGGATCTTTTGGTTGAAAATGAGGGTTTACCGGCCGGCCT G GAGA T TGT

Promoter-Z101        GCGCATATATAGATACTAAAATGTAGTAAAATATATGACTAAATTGGACTTGTTATGAAT : 1732
Promoter-M38         GCGCATATATAGATACTAAAATGTAGTAAAATATATGACTAAATTGGACTTGTTATGAAT : 1732
Promoter-ZFJ         GCGCATATATAGATACTAAAATGTAGTAAAATATATGACTAAATTGGACTTGTTATGAAT : 1734
Promoter-Chen12-3    GCGCATATATAGATACTAAAATGTAGTAAAATATATGACTAAATTGGACTTGTTATGAAT : 1734
Promoter-S18         GCGCATATATAGATACTAAAATGTAGTAAAATATATGACTAAATTGGACTTGTTATGAAT : 1732
Promoter-ZL          GCGCATATATAGATACTAAAATGTAGTAAAATATATGACTAAATTGGACTTGTTATGAAT : 1732
Promoter-BZ          GCGCATATATAGATACTAAAATGTAGTAAAATATATGACTAAATTGGACTTGTTATGAAT : 1732
Promoter-THIP        GCGCATATATAGATACTAAAATGTAGTAAAATATATGACTAAATTGGACTTGTTATGAAT : 1732
Promoter-Chen12-4    GCGCATATATAAATACTAAAATGTAGTAAAATATATGACTAAATTGGACTTGTCATGAAT : 1793
Promoter-LJBN002     GCGCATATATAGATACTAAAATGTAGTAAAATATATGACTAAATTGGACTTGTCATGAAT : 1763
Consensus            GCGCATATATA ATACTAAAATGTAGTAAAATATATGACTAAATTGGACTTGT ATGAAT
```

References

Jung S. (2019). Identification of genetic factors controlling anthocyanin biosynthesis in Capsicum annuum. Dissertation; Seoul National University.

Lim, S., Kim, D., Kim, J., Lee, J., Ha, S. (2017). A Radish Basic Helix-Loop-Helix Transcription Factor, RsTT8 Acts a Positive Regulator for Anthocyanin Biosynthesis. Front Plant Sci. 8, 1917. doi: 10.3389/fpls.2017.01917

Liu, H., Su, B., Zhang, H., Gong, J., Zhang, B., Liu, Y., et al. (2019). Identification and Functional Analysis of a Flavonol Synthase Gene from Grape Hyacinth. Molecules. 24(8):1579. doi: 10.3390/molecules24081579.

Zhang, Z., Li, D., Jin, J., Yin, Y., Zhang, H., Chai, W., et L. (2015). VIGS approach reveals the modulation of anthocyanin biosynthetic genes by CaMYB in chili pepper leaves. Front Plant Sci. 6:500. doi: 10.3389/fpls.2015.00500.

Zhong, C., Tang, Y., Pang, B., Li, X., Yang, Y., Deng, J., et al. (2020). The R2R3-MYB transcription factor GhMYB1a regulates flavonol and anthocyanin accumulation in Gerbera hybrida. Hortic Res. 7(1), 78. doi: 10.1038/s41438-020-0296-2.
