# Supplementary figures and images for: Identification of CaPs locus involving in purple stripe formation on unripe fruit, reveals allelic variation and alternative splicing of R2R3-MYB transcription factor in pepper (Capsicum annuum L.)
Source: Front Plant Sci. 2023 Mar 28;14:1140851. doi: 10.3389/fpls.2023.1140851 (PMC10089288; doi:10.3389/fpls.2023.1140851)

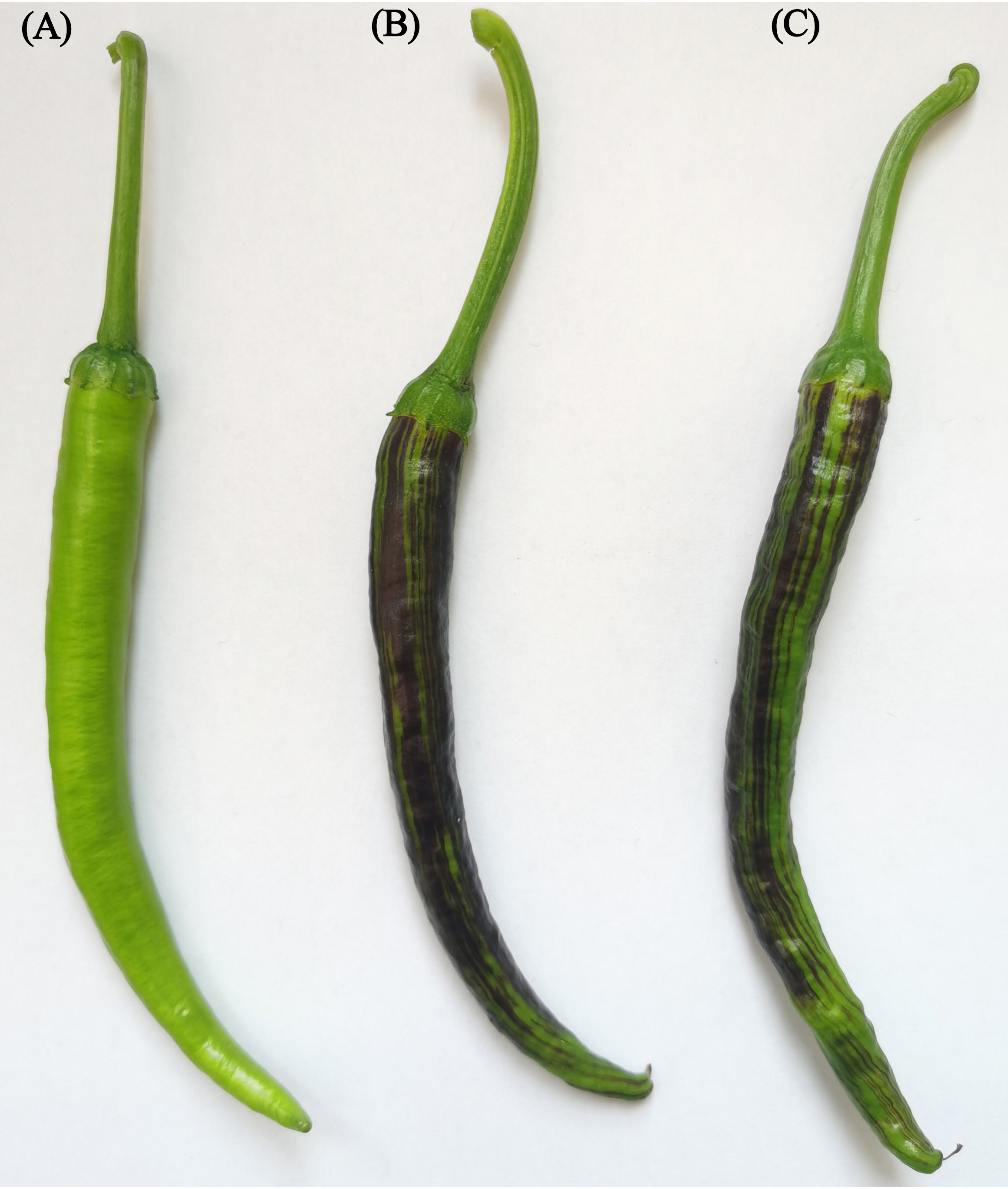

Supplement: Supplementary file 2 [file Image_1.jpeg]

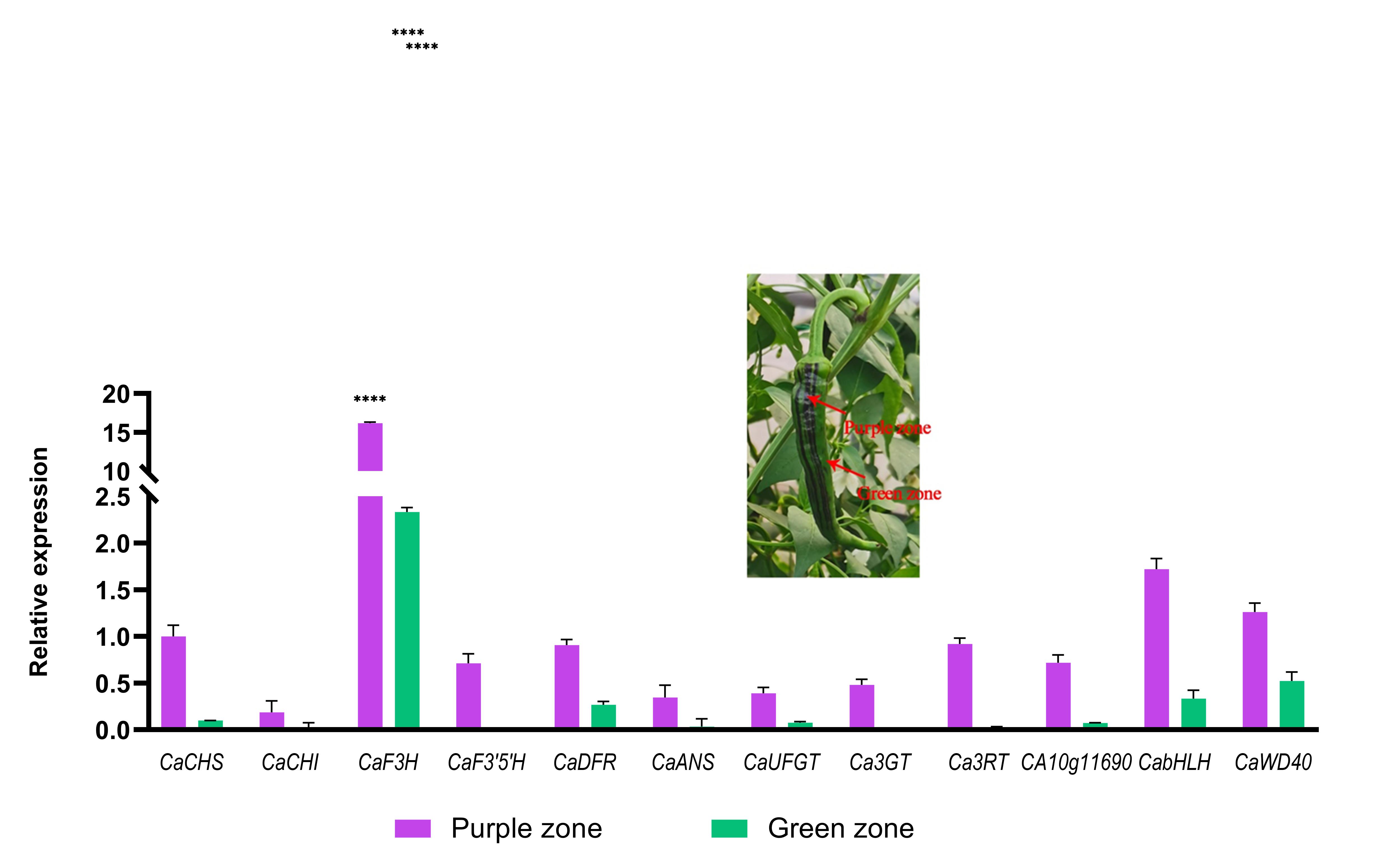

Supplement: Supplementary file 3 [file Image_2.jpeg]

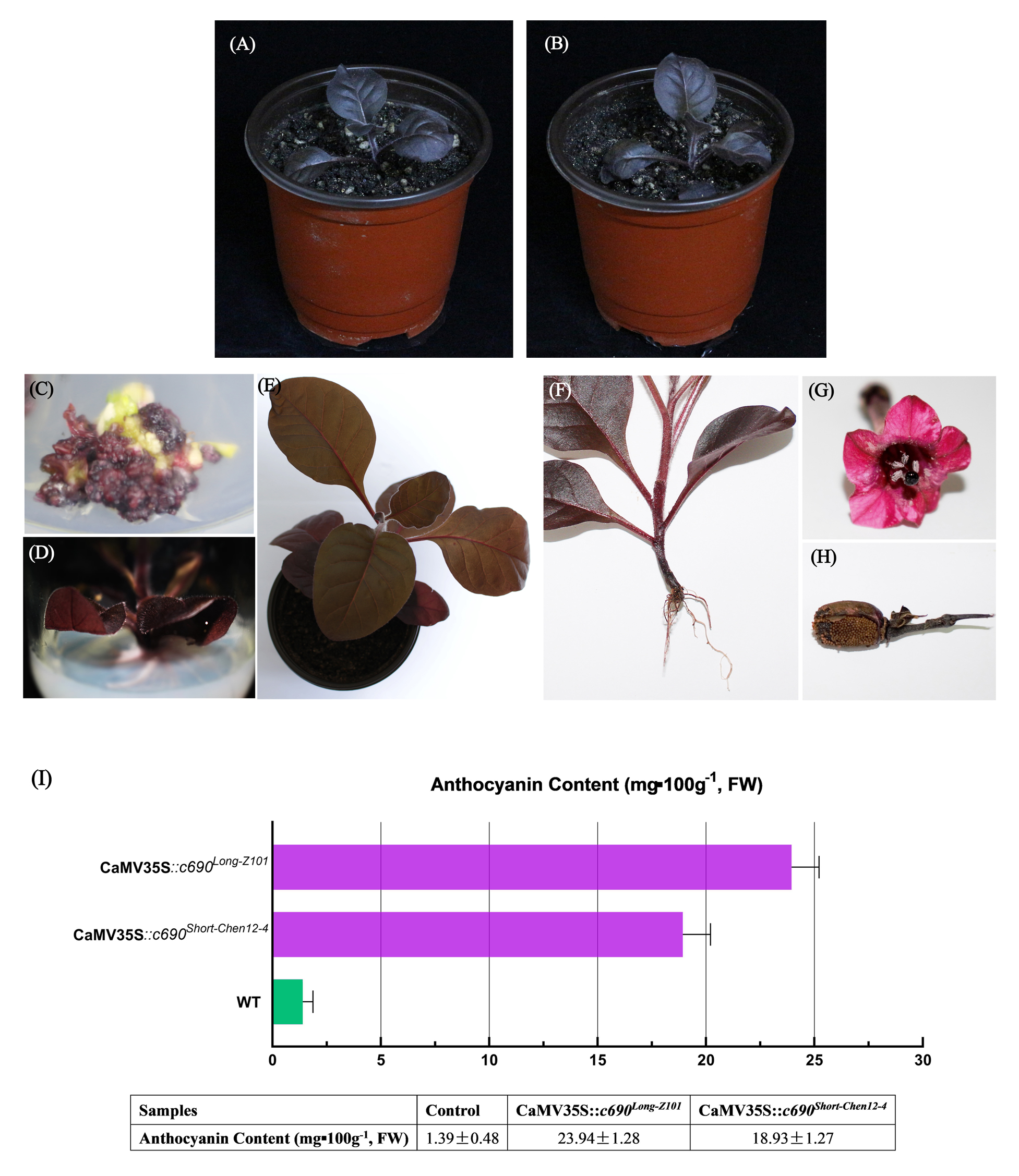

Supplement: Supplementary file 4 [file Image_3.jpeg]

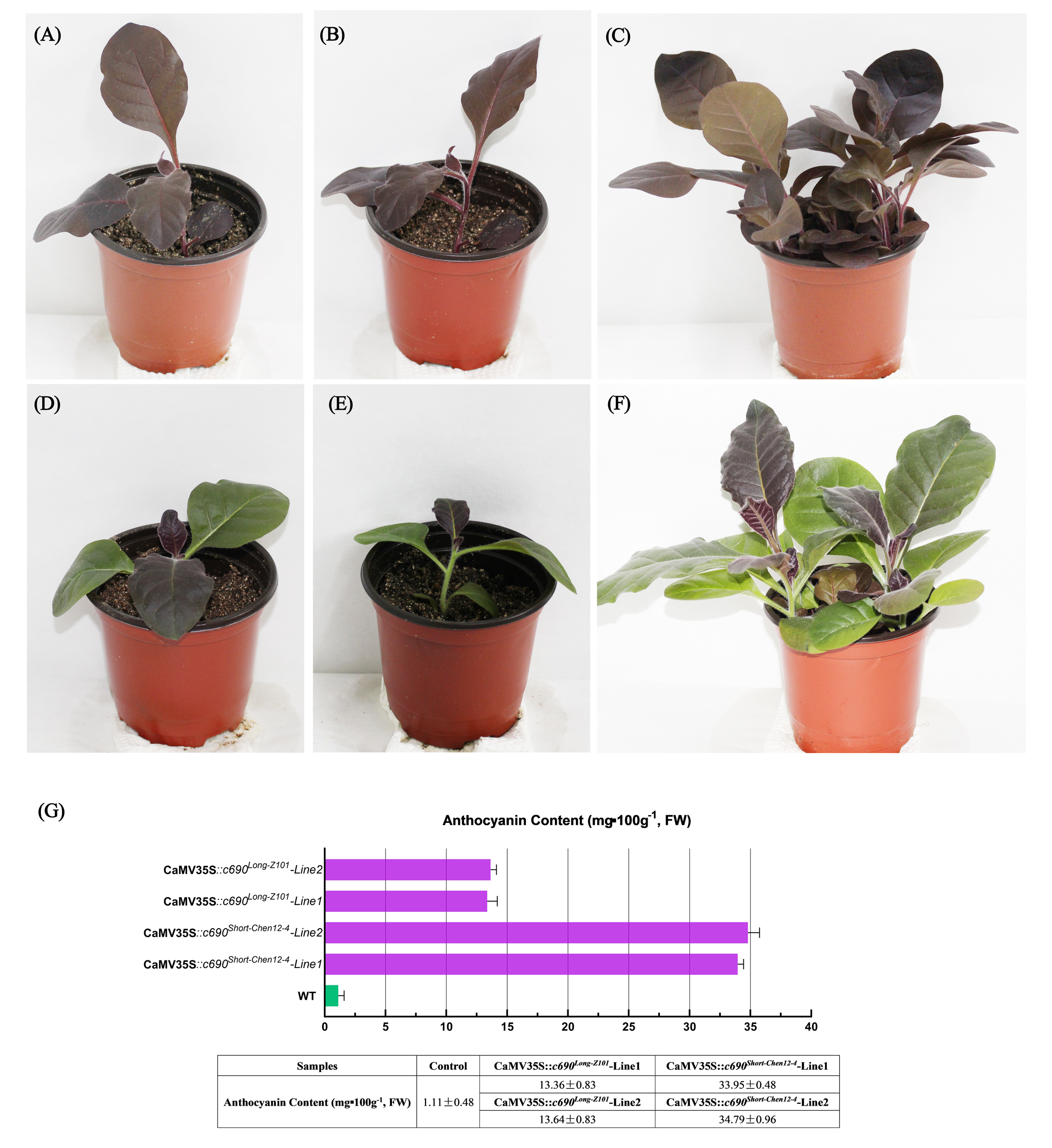

Supplement: Supplementary file 5 [file Image_4.jpeg]

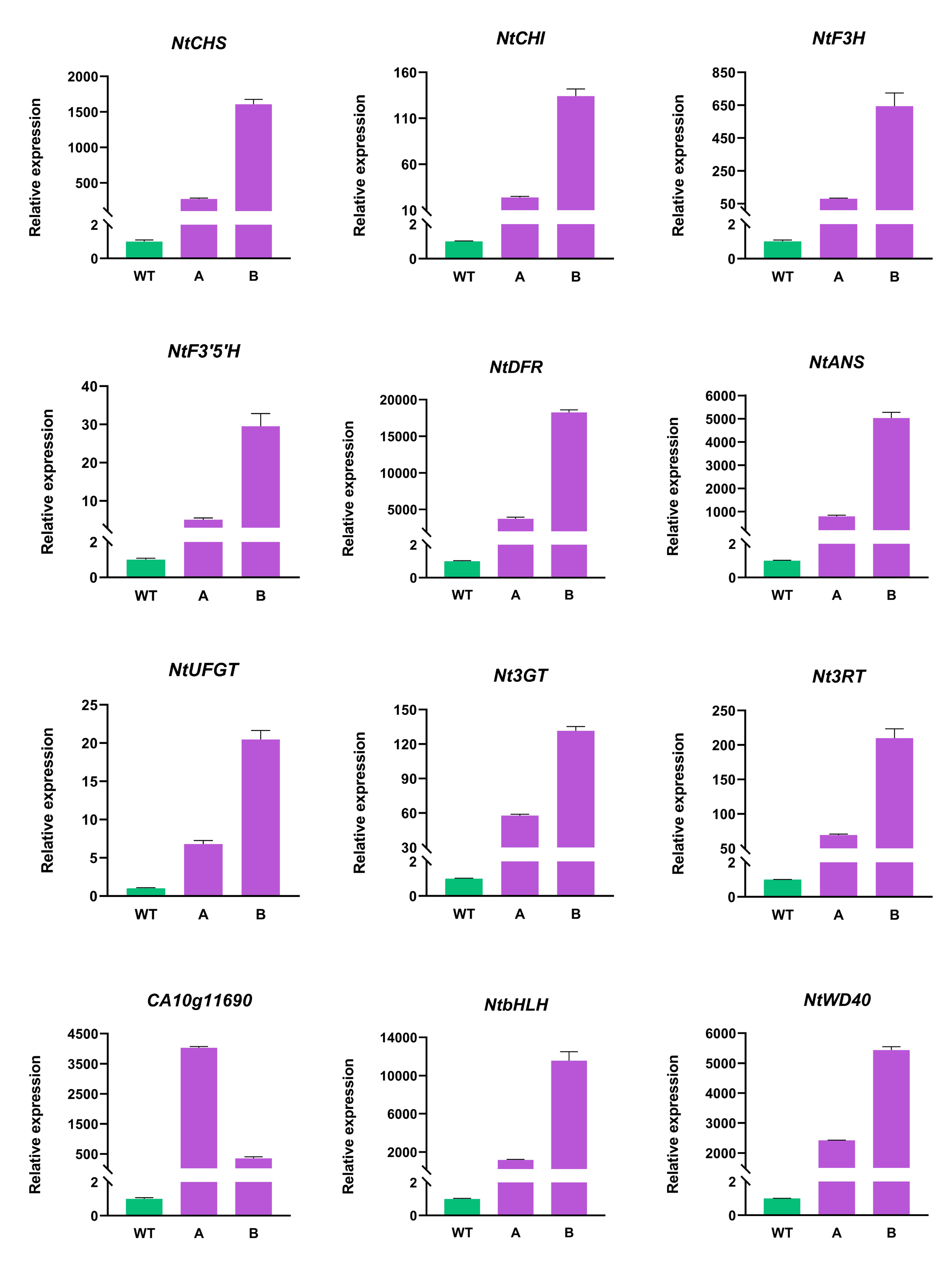

Supplement: Supplementary file 6 [file Image_5.jpeg]

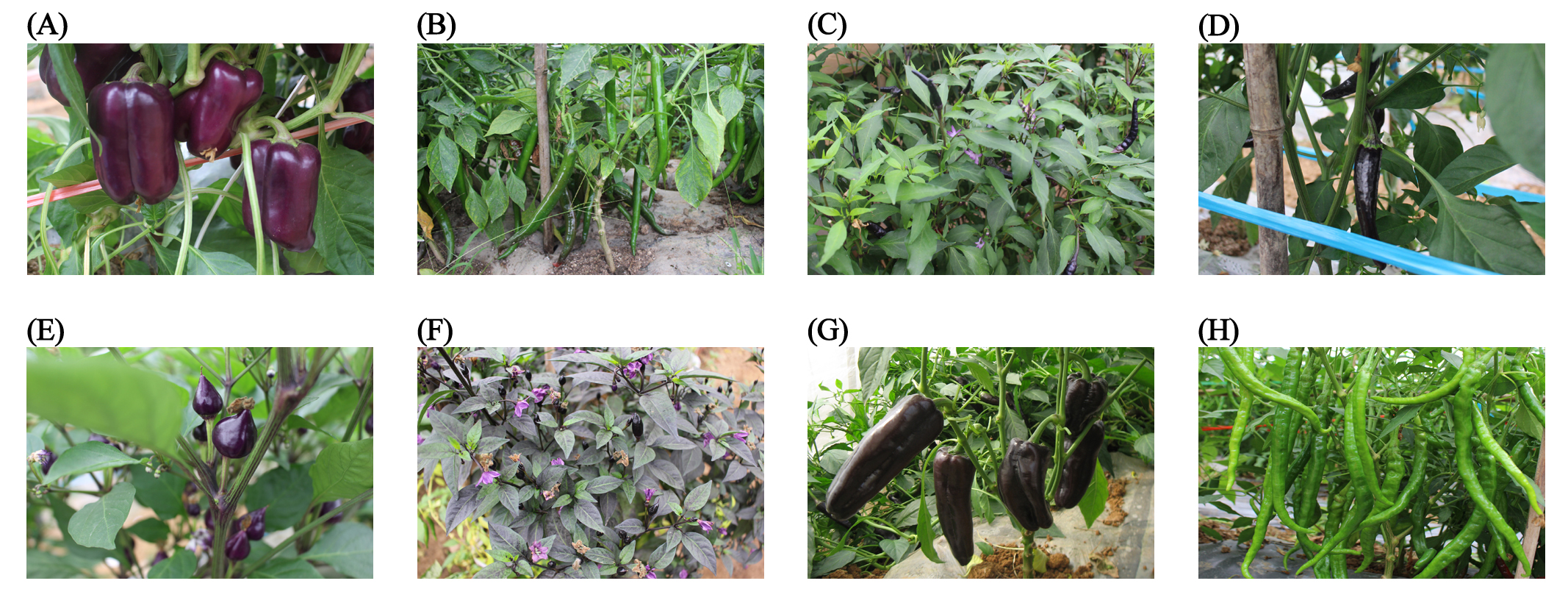

Supplement: Supplementary file 7 [file Image_6.jpeg]

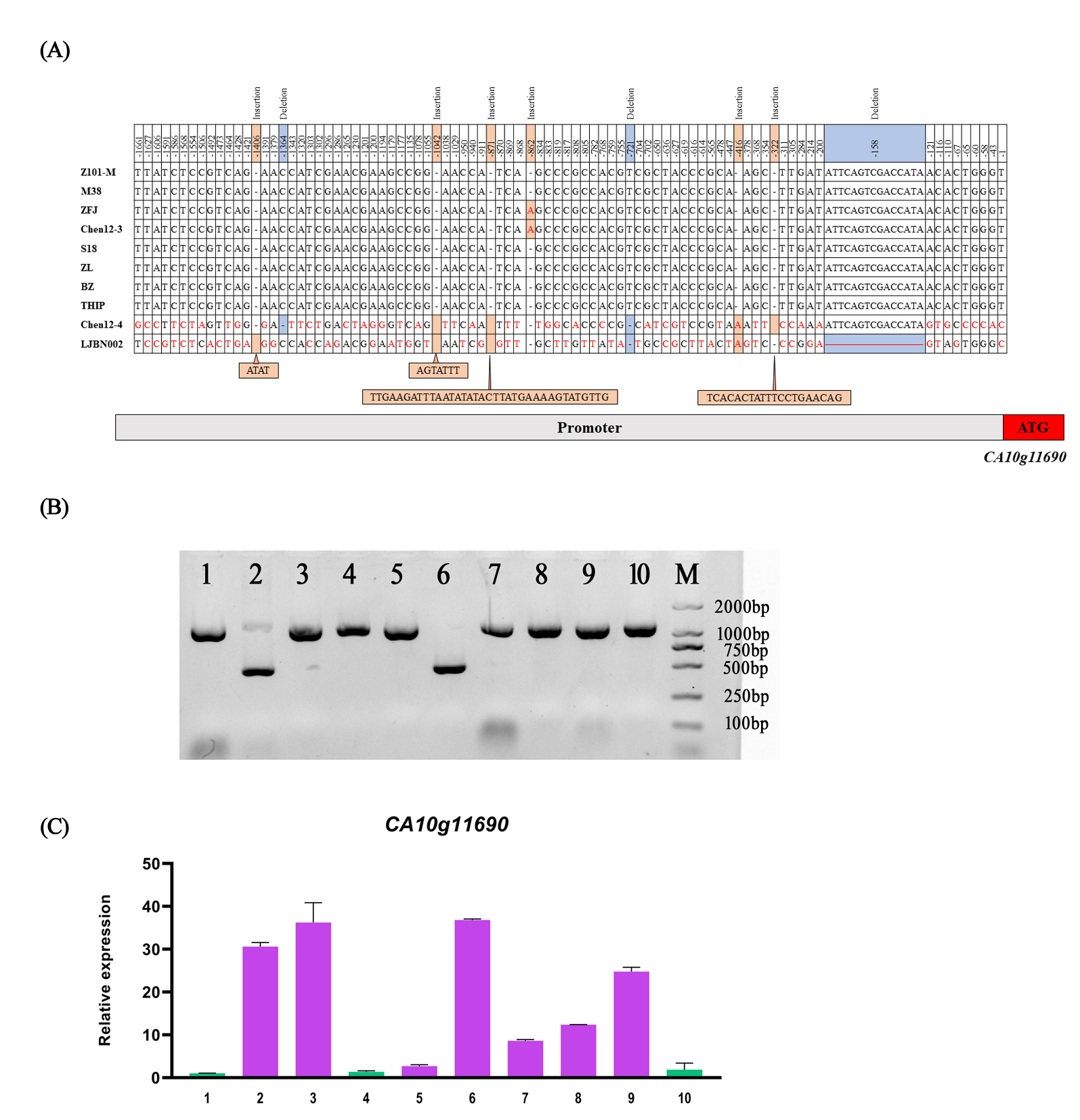

Supplement: Supplementary file 8 [file Image_7.jpeg]

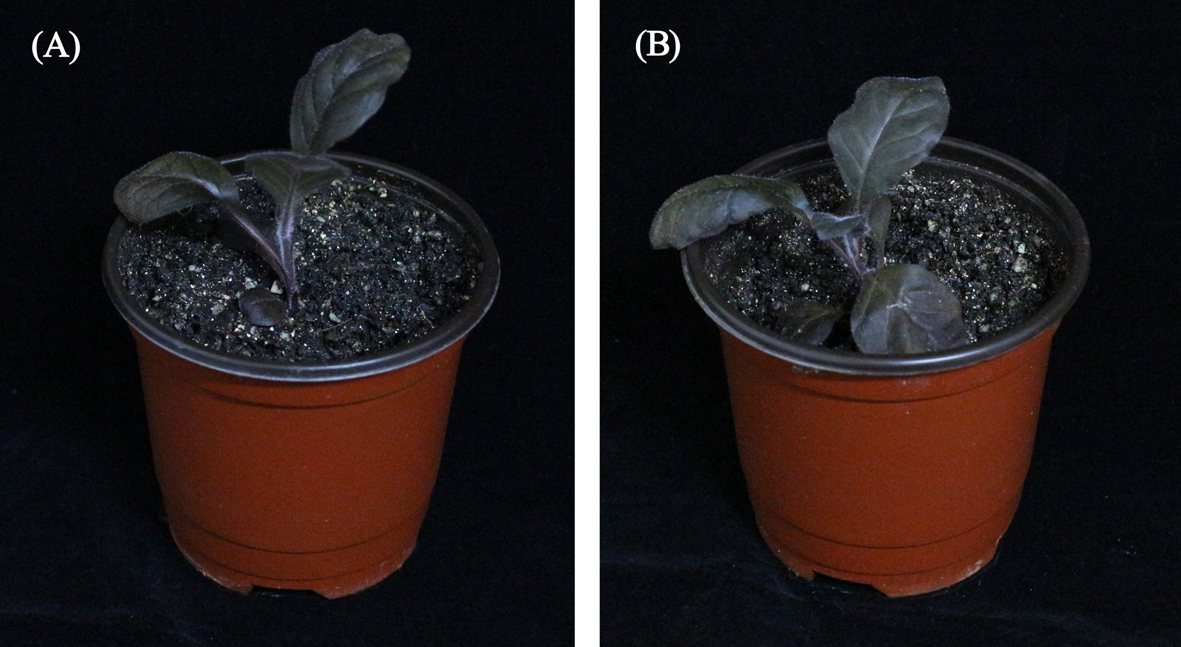

Supplement: Supplementary file 9 [file Image_8.jpeg]

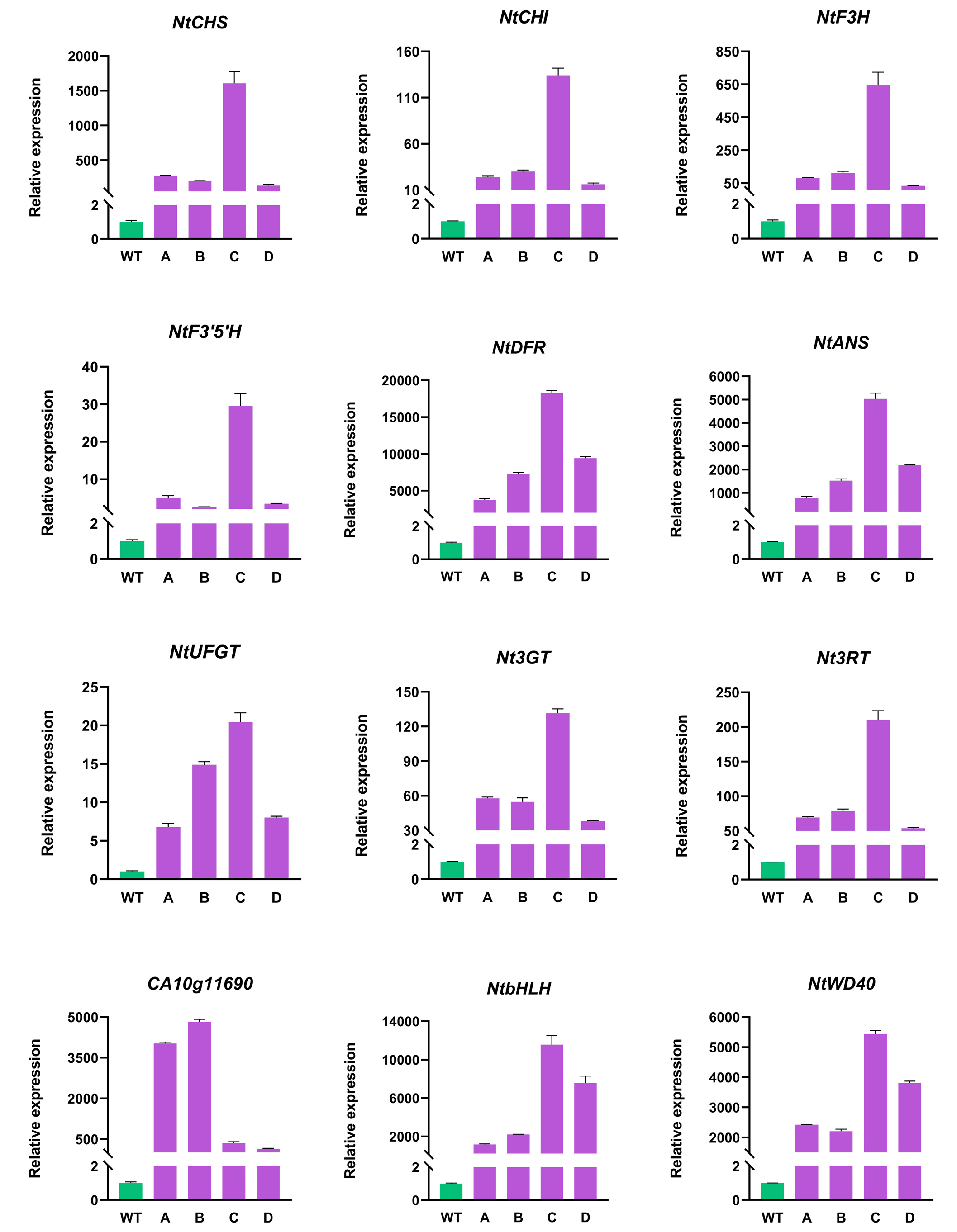

Supplement: Supplementary file 10 [file Image_9.jpeg]
